# Species-specific disordered regions and temperature dependence of bisecting GlcNAc-synthetic enzyme MGAT3

**DOI:** 10.1016/j.jbc.2026.113604

**Published:** 2026-09-23

**Authors:** Wanxue Bao, Shunji Natsuka, Anne Harduin-Lepers, Masamichi Nagae, Takahiro Yamasaki, Atsushi Iwasawa, Kyoko Matsuzawa, Yasuhiko Kizuka

**Affiliations:** 1 Institute for Glyco-core Research (iGCORE), Gifu University, Gifu, Japan; 2 Faculty of Science, Niigata University, Niigata, Japan; 3 Univ. Lille, Centre National de la Recherche Scientifique **(**CNRS), Unité Mixte de Recherche (UMR) 8576 - UGSF - Unité de Glycobiologie Structurale et Fonctionnelle, Lille, France; 4 Institute of Molecular Biomembrane and Glycobiology, Tohoku Medical and Pharmaceutical University, Sendai, Japan; 5 Faculty of Applied Biological Sciences, Gifu University, Gifu, Japan

**Keywords:** glycobiology, glycosyltransferase, *N-*glycan, bisecting GlcNAc, MGAT3 (GnT-III), non-conserved regions, catalytic activity, thermal stability

## Abstract

MGAT3 (GnT-III) is a glycosyltransferase that catalyzes the transfer of *N*-acetylglucosamine (GlcNAc) to the central β-mannose in *N*-glycans, generating bisecting GlcNAc. These bisecting GlcNAc-bearing *N*-glycans are implicated in cancer and Alzheimer’s disease and negatively regulate *N*-glycan maturation. Although the biological significance of bisecting GlcNAc has been increasingly elucidated in mammals, the structure and regulation of MGAT3 remain unclear. We recently found disordered Loop and Tail regions in human MGAT3 that affect its catalytic activity, suggesting the unique structure and mechanism of action of MGAT3. In this study, we extend our analysis to MGAT3 orthologs from various species, including mammals, reptiles, amphibians, and fish. Sequence comparisons and predicted structure analyses showed that the catalytic domain is conserved, whereas the Loop and Tail regions are highly divergent among species. Enzymatic analyses revealed that MGAT3 orthologs exhibit distinct activities and optimal temperature ranges. In particular, *Xenopus* MGAT3 displayed lower thermostability than human MGAT3 and showed almost no activity under standard enzyme assay conditions, only being active at low temperature. Deep *N*-glycomic analysis revealed that bisecting GlcNAc-containing *N*-glycans were abundantly present in *Xenopus* brain, suggesting that amphibian MGAT3 generates bisecting GlcNAc under native conditions at low temperature. We further found that zebrafish (*Danio rerio*) MGAT3 enzymes also have distinct temperature dependence compared with mammalian enzymes. These findings indicate that MGAT3 orthologs possess species-specific structures and temperature dependence, providing insight into the functional diversity and adaptive changes of MGAT3 during vertebrate evolution.

*N*-glycosylation, a major protein modification, occurs across eukaryotes (1) and plays essential roles in multiple biological processes by modulating protein function and maintaining protein quality control (2). *N*-glycosylation begins with the transfer of a preassembled oligosaccharide (Glc3Man9GlcNAc2) to the asparagine residue within the N-X-S/T sequon (X≠P) of nascent polypeptides in the endoplasmic reticulum (3). Subsequent processing and maturation of *N*-glycans occur in the Golgi apparatus through the coordinated actions of various glycosidases and glycosyltransferases (3). The resultant diverse *N*-glycan structures are essential for numerous biological processes, and abnormalities in the *N*-glycan maturation pathway are related to a wide spectrum of diseases, such as diabetes (4) and Alzheimer’s disease (5).

In vertebrates, *N*-glycans have variable numbers of branches synthesized by specific *N*-acetylglucosamine (GlcNAc) transferases (MGAT1–5, also designated as GnT-I–V) (6, 7). The core biantennary structure of *N*-glycan is highly conserved, while a species-specific branch is also present (8). Among the enzymes for *N*-glycan branching, our present study focuses on MGAT3 (GnT-III) which catalyses the transfer of GlcNAc to the β-mannose residue *via* β1,4-linkage, thereby generating the “bisecting GlcNAc” structure (9) (Fig. 1*A*). MGAT3 is abundantly expressed in the brain and kidney of rodents (10, 11) and regulates the functions of substrate glycoproteins implicated in the onset and progression of several diseases. For example, in Alzheimer’s disease model mice, genetic deletion of *Mgat3* impairs the *in vivo* activity of BACE1, leading to reduced accumulation of the pathogenic amyloid-β peptide in the brain and improved Alzheimer’s disease pathology (12, 13). In the kidney, proteins involved in angiotensin-II metabolism were found to be modified by MGAT3, and more severe renal damage was observed when angiotensin-II-related tubulointerstitial dysfunction was induced in *Mgat3* knockout (KO) mice than in wild-type (WT) mice (14). Moreover, bisecting GlcNAc was reported to be a general suppressor of *N*-glycan maturation in cells, and the presence of bisecting GlcNAc on *N*-glycans inhibits the formation of additional branches (15, 16) and terminal glycan epitopes (17), suggesting that MGAT3 plays a crucial role in shaping the *N*-glycan profiles of proteins and cells. Collectively, these findings highlight the biological importance of bisecting GlcNAc.

**Figure 1:**
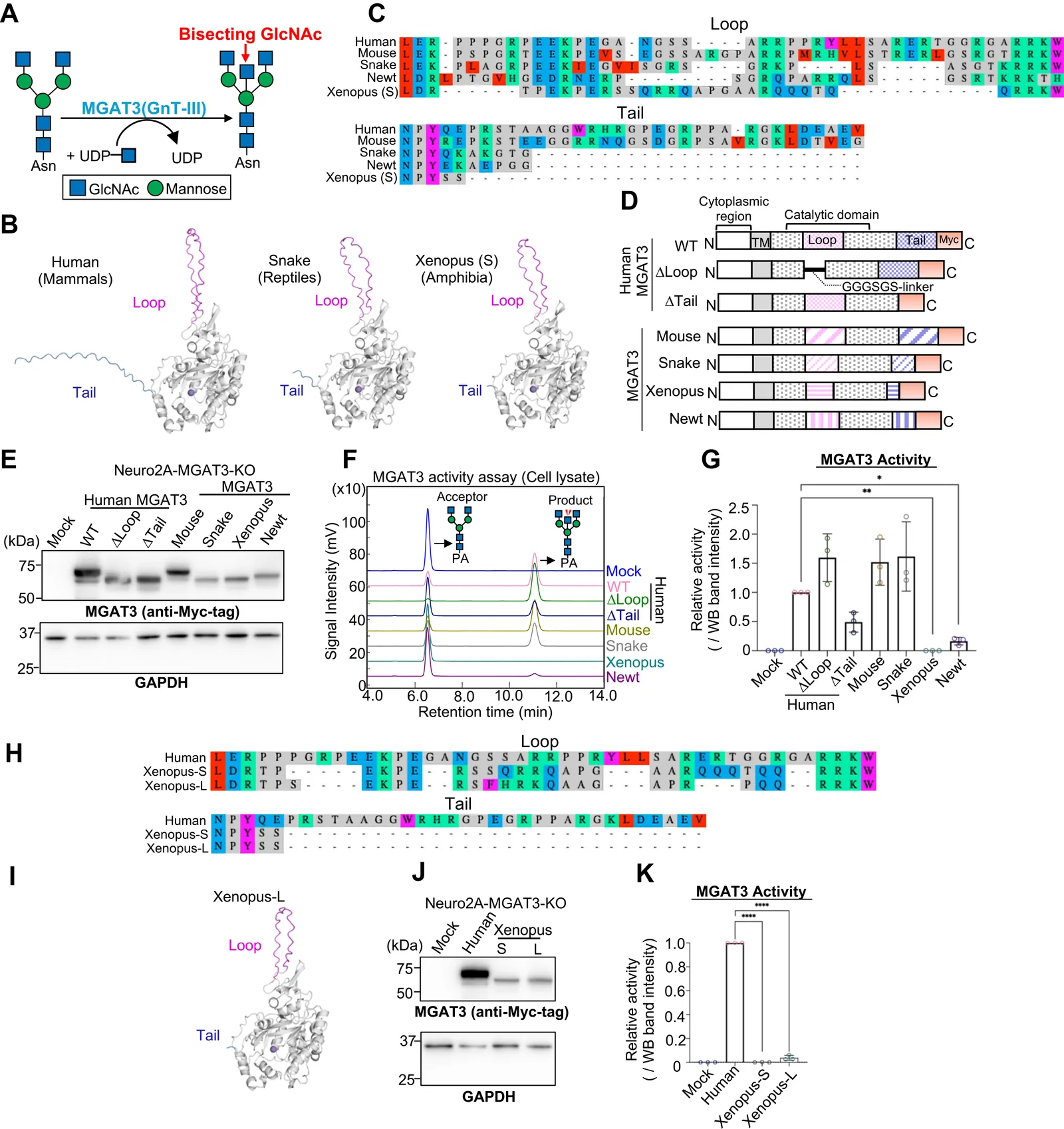
***In vitro* activity of MGAT3 enzymes derived from various vertebrate species**. *A*, schematic diagram of bisecting GlcNAc formation by MGAT3. *B*, the 3D structures of MGAT3s from human, snake, and *Xenopus* were predicted by AlphaFold2. Light gray, transmembrane and catalytic domains; magenta, Loop; blue, Tail; the purple sphere, the catalytic center (E320) predicted in a previous study (22). *C*, Alignment of the Loop and Tail sequences derived from several species. The hyphens (−) indicate gaps. *D*, plasmid constructs used for cellular experiments. *E*, the Myc-tagged human MGAT3 (WT), Loop and Tail deletion mutants (ΔLoop and ΔTail), and the Myc-tagged mouse, snake, *Xenopus*, and newt MGAT3 were expressed in Neuro2A-MGAT3-KO cells. The empty vector (pcDNA6-mycHisA) was transfected into Neuro2A-MGAT3-KO cells as a control (*Mock*). Proteins in the cell lysates were blotted with anti-Myc-tag (*upper*) and anti-GAPDH (*lower*) antibodies. *F*, cell lysates prepared as described in (*E*) were reacted with the fluorescently labeled oligosaccharide acceptor substrate, GnGnbi-PA. After the reaction, the unreacted substrate and products with bisecting GlcNAc were quantified by reversed phase HPLC. *G*, activity of MGAT3s was calculated according to the peak areas in (*F*) and divided by the corresponding signal intensity of MGAT3 in (*E*). The activity relative to that of human MGAT3-WT is shown (n = 3, mean ± SD, ∗*p* < 0.05, *∗∗p* < 0.005, one-way ANOVA with Dunnett’s test). *H*, alignment of the Loop and Tail sequences among human MGAT3 and *Xenopus* MGAT3-S and -L. *I*, The 3D structure of *Xenopus* MGAT3-L. *J*, the Myc-tagged human MGAT3 and *Xenopus* MGAT3-S and -L were expressed in Neuro2A-MGAT3-KO cells. The empty vector was transfected into Neuro2A-MGAT3-KO cells as a control (*Mock*). Proteins in the cell lysates were blotted with anti-Myc-tag (*upper*) and anti-GAPDH (*lower*) antibodies. *K*, Activity of MGAT3s was calculated according to the peak areas shown in Figure S3 and divided by the corresponding signal intensity of MGAT3 in (*J*). The activity relative to that of human MGAT3-WT is shown (n = 3, mean ± SD, ∗∗∗∗*p* < 0.0001, one-way ANOVA with Dunnett’s test).

Despite the biological significance of bisecting GlcNAc, the mechanisms governing MGAT3 activity remain largely unclear. To address this question, it is crucial to clarify the unsolved structure and domain organization of MGAT3. Our recent biochemical and structural studies on other mammalian glycosyltransferases revealed that many enzymes involved in *N*-glycan biosynthesis possess unique non-catalytic domains or regions for modulating their functions, such as the recognition of protein substrates (18), the regulation of catalytic activity (19, 20), and homo-oligomerization (21). Although the 3D structure of MGAT3 has not yet been experimentally determined, the above findings prompted us to investigate the structure–function relationship of MGAT3 to understand the regulation mechanism of MGAT3 activity in more detail.

We recently compared the AlphaFold-predicted structure of human MGAT3 with the crystal structures of MGAT1 and MGAT2 and proposed the catalytic center of MGAT3 (E320) (22). Furthermore, we found that human MGAT3 contains two unique and likely disordered segments in the predicted structure (23): a long loop spanning L125 to W168 in the middle region (designated as “Loop”), and a C-terminal tail ranging from R506 to V533 (designated as “Tail”) (Fig. 1*B*). By examining deletion mutants lacking either the Loop or Tail, we revealed that the Loop suppresses the catalytic activity of MGAT3 and is required for its usual shedding, whereas the Tail is required for proper folding of MGAT3 (23). These findings suggest that regions outside of the catalytic domain are critical for regulating MGAT3 functions. However, the detailed mechanisms by which these two disordered regions regulate MGAT3 activity are unclear. In addition, little is known about the extent to which the activity and structure of MGAT3 are conserved among non-mammalian vertebrates. Our recent findings on another *N*-glycan branching enzyme MGAT4 family revealed that the differences in their structures and activities among species contribute to the biosynthesis of species-specific patterns of *N*-glycan branching (8). Therefore, comparison of the activities and sequences, including the Loop and Tail, of MGAT3 from various species could provide new insights into how the Loop and Tail regions regulate MGAT3 activity and how bisecting GlcNAc is biosynthesized in various species.

In this study, we performed multiple sequence alignments of MGAT3 orthologs from diverse vertebrate species and found that the Loop and Tail regions are particularly divergent. Notably, our enzymatic analysis demonstrated that amphibian and teleost MGAT3 have largely distinct temperature dependence and Loop and Tail sequences from mammalian MGAT3. These findings suggest that species-dependent diversification of the sequence and structure of MGAT3 has contributed to the adaptive changes in temperature-dependent activity during vertebrate evolution.

## Results

### The non-conserved regions and species-dependent activity of MGAT3

First, to explore the overall similarity of MGAT3 among various species, we compared the amino acid sequences and AlphaFold-predicted structures of MGAT3 orthologs from representative vertebrate species. The predicted structures of human, snake (*Thamnophis sirtalis*), and *Xenopus* (*Xenopus laevis*) MGAT3 revealed marked differences in the lengths and predicted structures of the Loop and Tail regions compared with other regions (Fig. 1*B*). Sequence alignment confirmed that both the Loop and Tail regions are poorly conserved among species, with marked variations in amino acid sequence (Figs. 1*B* and S1). In particular, Tail length differed markedly among the species examined compared with the long Tail in mammalian MGAT3 (Figs. 1, *B* and *C* and S1, *B* and C). To examine the activity of MGAT3 orthologs, human, mouse (*Mus musculus*), snake, *Xenopus*, and newt (*Pleurodeles waltl*) MGAT3, together with human MGAT3 mutants lacking either the Loop (ΔLoop) or Tail (ΔTail), were expressed in Neuro2A-MGAT3-KO cells (Fig. 1, *D* and *E*). The *in vitro* MGAT3 activity toward an *N*-glycan substrate in the cell lysates was measured according to an established method (7) using a fluorescence (PA, pyridylamine)-labeled oligosaccharide substrate and high-performance liquid chromatography (HPLC; Fig. 1, *F* and *G*). Consistent with our previous study, deletion of the Loop region increased human MGAT3 activity, whereas deletion of the Tail region reduced MGAT3 activity compared with the WT enzyme (Fig. 1*G*), indicating that these non-conserved regions differentially modulate catalytic function (23). Among the orthologs of MGAT3, the mouse and snake enzymes exhibited activity comparable to that of the human enzyme, whereas *Xenopus* and newt MGAT3 showed no or little activity under the same assay conditions (Fig. 1*G*).

*X. laevis* possesses two MGAT3 homologs: MGAT3-S and MGAT3-L, due to allotetraploidization of the genome (24, 25). Our structural prediction and sequence alignment showed high structural and sequence conservation between *Xenopus* MGAT3-S and -L, even though several small differences existed in the Loop region (Figs. 1, *H*, and *I* and S2). We further compared the *in vitro* activity of the S and L forms of *Xenopus* MGAT3 expressed in Neuro2A-MGAT3-KO cells and found no activity and trace levels of activity, respectively, under the same conditions as human MGAT3 (Figs. 1, *J* and *K*, and S3). These results suggest divergence of the Loop and Tail sequences and MGAT3 activity during vertebrate evolution.

### *Xenopus* MGAT3 exhibits high *in vivo* activity

To examine whether *Xenopus* MGAT3 is inactive or active under certain conditions, we next examined the *in cellulo* biosynthetic activity of snake, *Xenopus*, and newt MGAT3 expressed in Neuro2A-MGAT3-KO cells. The cell lysates were blotted with E4-phytohemagglutinin (E4-PHA), which preferentially recognizes bisecting GlcNAc (26). Unexpectedly, intense signals corresponding to E4-PHA were detected in *Xenopus* MGAT3-expressing cells (Fig. S4), suggesting that *Xenopus* MGAT3 actively synthesizes bisecting GlcNAc *in cellulo*.

MGAT3 is highly expressed in the brain and kidney of rodents (10, 11); therefore, we examined *in vitro* MGAT3 activity and the level of bisecting GlcNAc in *X. laevis* brain, compared with those in the brain and kidney of mice. Lysates of membrane fractions of *X. laevis* brain, and the brain and kidney of WT and *Mgat3* KO mice, were reacted with a PA-labeled oligosaccharide substrate, and the product with bisecting GlcNAc was quantified by HPLC (Figs. 2*A* and S5*A*). Compared with the brain and kidney from WT mice, *X. laevis* brain barely exhibited MGAT3 activity toward the *N*-glycan substrate under the same conditions as mouse enzymes (Fig. 2*A*). However, the same lysates of membrane fractions of *X. laevis* brain displayed intense signals in E4-PHA blotting (Fig. 2*B*). Since the E4-PHA blotting signals were largely reduced in *Mgat3* KO mice, our data indicate the presence of *N*-glycans with bisecting GlcNAc in *Xenopus* brain. To further confirm the presence of bisecting GlcNAc, we performed *N*-glycomics analysis of a *X. laevis* brain. *N*-glycans were released from the *X. laevis* brain, labeled with PA, and analyzed by Liquid Chromatography-Tandem Mass Spectrometry (LC-MS/MS, Figs. S6 and S7). Many bisecting GlcNAc-containing structures with varying degrees of branching, galactosylation, and fucosylation were identified (Figs. 2*C*, S8 and Table S1). Quantitative classification of these *N*-glycans showed that the bisecting GlcNAc-bearing complex and hybrid glycans represented 25.5% and 7.3% of the total *N*-glycans, respectively (Fig. 2*D*), similar to mouse brain (27). Our data demonstrate that bisecting GlcNAc-modified *N*-glycans are abundantly present in *X. laevis* brain, despite the low *in vitro* activity detected toward the oligosaccharide substrate under the above assay conditions.

**Figure 2:**
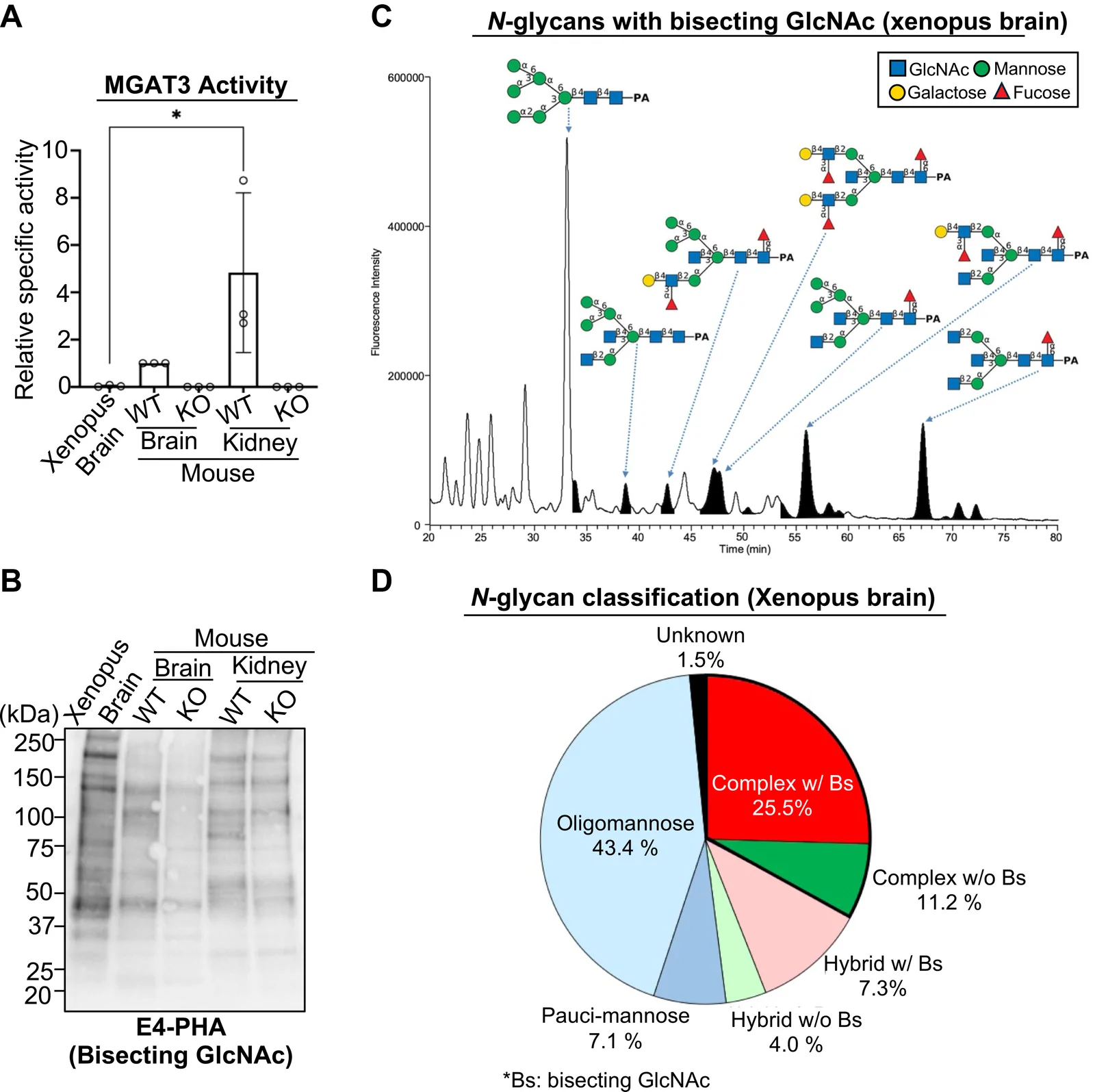
**Presence of bisecting GlcNAc-containing *N*-glycans in the *Xenopus laevis* brain.***A*, the MGAT3 activity of mouse and *Xenopus* tissues was calculated according to the peak areas shown in Figure S5*A* (n = 3, mean ± SD, *∗p* = 0.01, one-way ANOVA with Dunnett’s test). The activity relative to that of the WT mouse brain is shown. *B*, proteins in the membrane fraction of *Xenopus* brain (WT, male), mouse brain (WT: female, 16 w; *Mgat3* KO: male, 11 w), and mouse kidney (WT: male, 13 w; *Mgat3* KO: female, 13 w) were blotted with E4-PHA. *C*, reversed-phase chromatogram of fraction 1 in DEAE separation (Fig. S6). Several major glycan structures are illustrated. *Black-shaded peaks* indicate the glycans containing bisecting GlcNAc. The data of *N*-glycomics analysis of a *Xenopus laevis* brain is shown. *D, N*-glycan classification in the *Xenopus* brain based on the acquired *N*-glycomics data. The relative abundance of each *N*-glycan is shown in Table S1.

### Thermostability difference between human and *Xenopus* MGAT3

The discrepancy between the *in vitro* and *in vivo* activities of *Xenopus* MGAT3 indicated that specialized conditions were necessary for its catalytic action. As an ectotherm, *X. laevis* does not maintain a constant body temperature but its preferred temperature is 22 °C–23 °C (28). Therefore, we investigated the *in vitro* activity of *Xenopus* MGAT3 at different temperatures. Human, snake, and newt MGAT3 and *Xenopus* MGAT3-S and -L were expressed in Neuro2A-MGAT3-KO cells (Fig. S9*A*), and an activity assay was conducted using cell lysates at several temperatures (20 °C, 25 °C, 30 °C, and 37 °C) (Fig. S9, *B*–*E*). Unlike human MGAT3, which exhibited stable activity under the different reaction temperatures, the *in vitro* activity of *Xenopus* MGAT3-S and -L peaked at 20 °C and 25 °C, respectively (Figs. 3*A*, and S9, *F*, and *G*), showing the difference in temperature dependence between *Xenopus* and human MGAT3.

**Figure 3:**
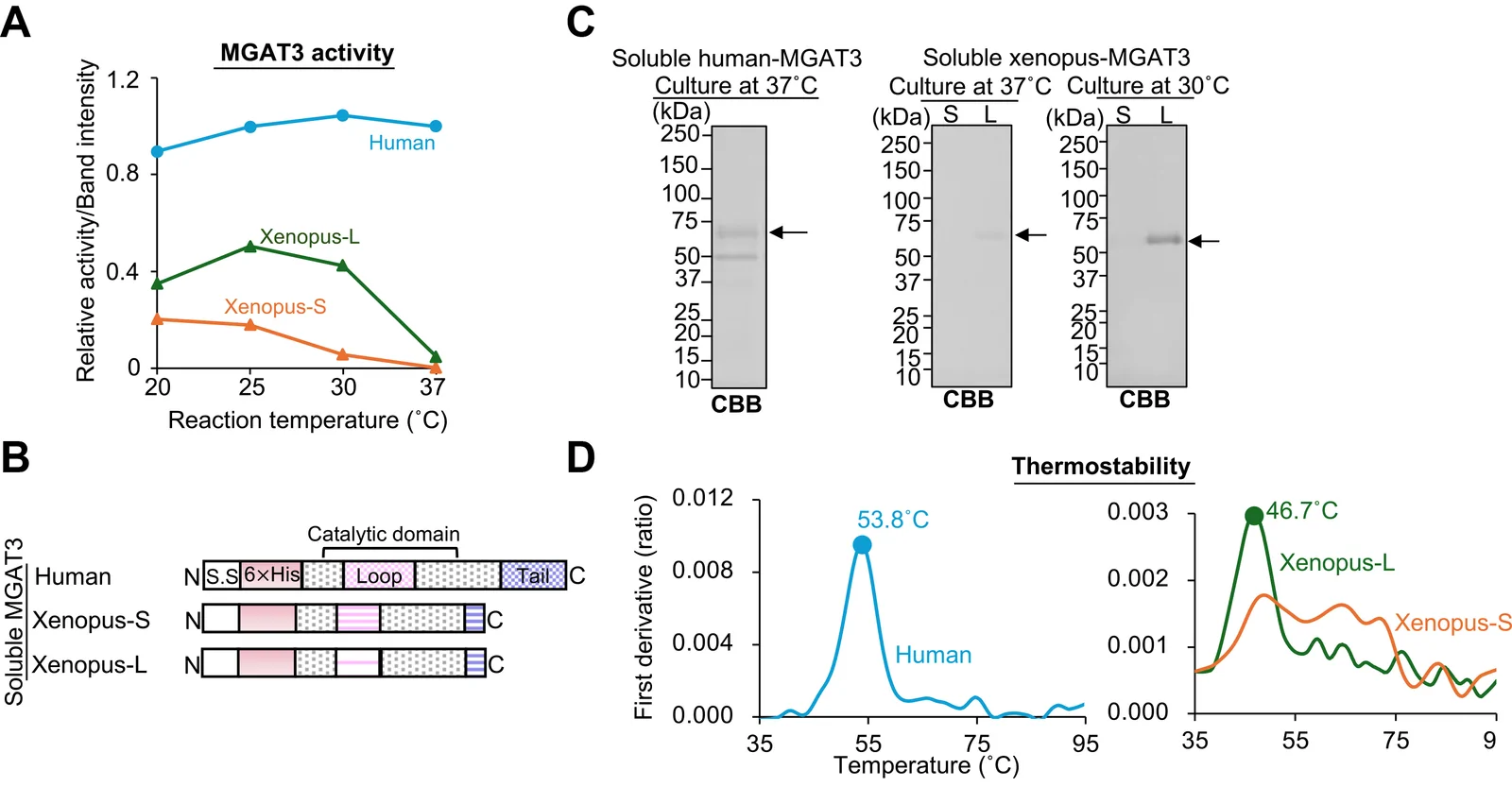
**Thermal stability of human and *Xenopus* MGAT3**. *A*, the activity of human MGAT3 and *Xenopus* MGAT3-S and -L at several temperatures was calculated according to the peak areas shown in Figure S9B–S9E and divided by the corresponding signal intensity of MGAT3 shown in Figure S9*A*. The activity relative to that of human MGAT3 at 37 °C is shown. *B*, plasmid constructs used for purification of soluble MGAT3. *C*, soluble human MGAT3 was expressed in HEK293T cells cultured at 37 °C, and soluble *Xenopus* MGAT3-S and -L were expressed in HEK293T cells cultured at 37 °C or 30 °C. Soluble MGAT3 enzymes were purified from the medium using a Ni^2+^ column, and the purified proteins were subjected to SDS-PAGE and CBB staining. *D*, the inflection temperatures of human MGAT3, and *Xenopus* MGAT3-S and -L were measured by differential scanning fluorometry. The profiles of the first derivative (ratio) of 350 nm/330 nm are shown.

The activity assay results suggest that *Xenopus* enzymes are less stable at high temperature than human MGAT3. To investigate the thermal stability of *Xenopus* MGAT3s, we expressed and purified soluble His-tagged human MGAT3-WT and *Xenopus* MGAT3-S and -L, from the culture media (Figure 3, Band C). When cells were cultured at 37 °C, the yield of *Xenopus* MGAT3s from the culture media was low (Fig. 3*C*, middle panel). When cells were cultured at 30 °C, the yield of *Xenopus* MGAT3-S was still very low, but the yield of *Xenopus* MGAT3-L was higher (Fig. 3*C*, right column). Although the low yield of *Xenopus* enzyme at 37 °C might be due to suppressed secretion or accelerated degradation, one of the possible reasons is that *Xenopus* MGAT3s are less stable at high temperature than the human enzyme.

A thermal stability assay was then performed by differential scanning fluorometry. Although it was difficult to obtain clear data for *Xenopus* MGAT3-S because of the low yield, our data demonstrated the lower inflection temperature of *Xenopus* MGAT3-L (46.7 °C) than human MGAT3 (53.8 °C) (Figs. 3*D* and S10), clearly indicating the lower thermal stability of *Xenopus* MGAT3-L than human MGAT3.

### pH and divalent cation dependence of human and *Xenopus* MGAT3

In addition to temperature dependence, we examined whether the optimal pH and divalent cation dependence differ between human and *Xenopus* MGAT3. The activity of the purified soluble human MGAT3 and *Xenopus* MGAT3-L enzymes was measured in reaction buffers at various pH values or with various divalent cations (Fig. S11). Human MGAT3 exhibited the highest activity at pH 6.3 (Fig. S11, *A* and *C*), which is consistent with previous studies on rat MGAT3 (29). Unlike humans, *Xenopus* MGAT3 showed the highest activity at pH 6.9 (Fig. S11, *A* and *D*). Since the optimal pH of chicken MGAT3 was reported to be 7.0 (30), the optimal pH of MGAT3 could be variable among species. We also found that the activities of both human and *Xenopus* MGAT3 required Mn^2+^ (Fig. S11, *B*, *E*, and *F*), but tolerance to other metals differed between human and *Xenopus* enzymes. Human MGAT3 was highly dependent on Mn^2+^ and exhibited only scarce activity in the presence of Mg^2+^, Ca^2+^, or Zn^2+^ (Fig. S11, *B* and *E*). In contrast, *Xenopus* MGAT3 retained its activity in the presence of Mg^2+^ or Ca^2+^ to approximately half of that observed in the presence of Mn^2+^ (Fig. S11, *B* and *F*). Our data indicate that human and *Xenopus* MGAT3 differ not only in temperature but also in pH and divalent cation dependence.

### Swapping the loop or tail regions does not change temperature dependence

Based on our sequence and structure comparisons (Fig. 1), we hypothesized that the non-conserved Loop and Tail regions might contribute to the different temperature dependence and thermostabilities of *Xenopus* and human MGAT3. To test this possibility, we constructed plasmids of chimeric MGAT3s in which the Loop or Tail regions were swapped between human and *Xenopus* enzymes or the Loop region was deleted from *Xenopus* MGAT3 (Fig. 4*A*). These chimeric enzymes and mutants were expressed in Neuro2A-MGAT3-KO cells (Fig. 4, *B* and *D*), and MGAT3 activity in the cell lysates was measured at 25 °C and 37 °C (Fig. S12). Neither the human MGAT3 Loop nor Tail region rescued the *in vitro* activity of *Xenopus* MGAT3-S and -L at 37 °C (Fig. 4, *C* and *E*). Chimeric *Xenopus* MGAT3s bearing the human Loop or Tail region showed similar activity profiles and temperature dependence to *Xenopus* WT enzymes (Fig. 4, *C* and *E*), and the chimeric human MGAT3s bearing *Xenopus* Loop or Tail regions also showed similar activities to human MGAT3 WT (Fig. 4, *C* and *E*). Our data indicated that swapping the Loop or Tail region alone did not change species-specific temperature dependence, although these regions may modulate the enzymatic activity. Notably, the Loop deletion mutant of *Xenopus* MGAT3 (ΔLoop) showed increased activity compared with that of *Xenopus* MGAT3-WT, particularly for *Xenopus* MGAT3-S (Fig. 4*C*). This suggests that the Loop region in *Xenopus* MGAT3-S contributes to the weak activity of this enzyme, consistent with our previous finding for human MGAT3 (23).

**Figure 4:**
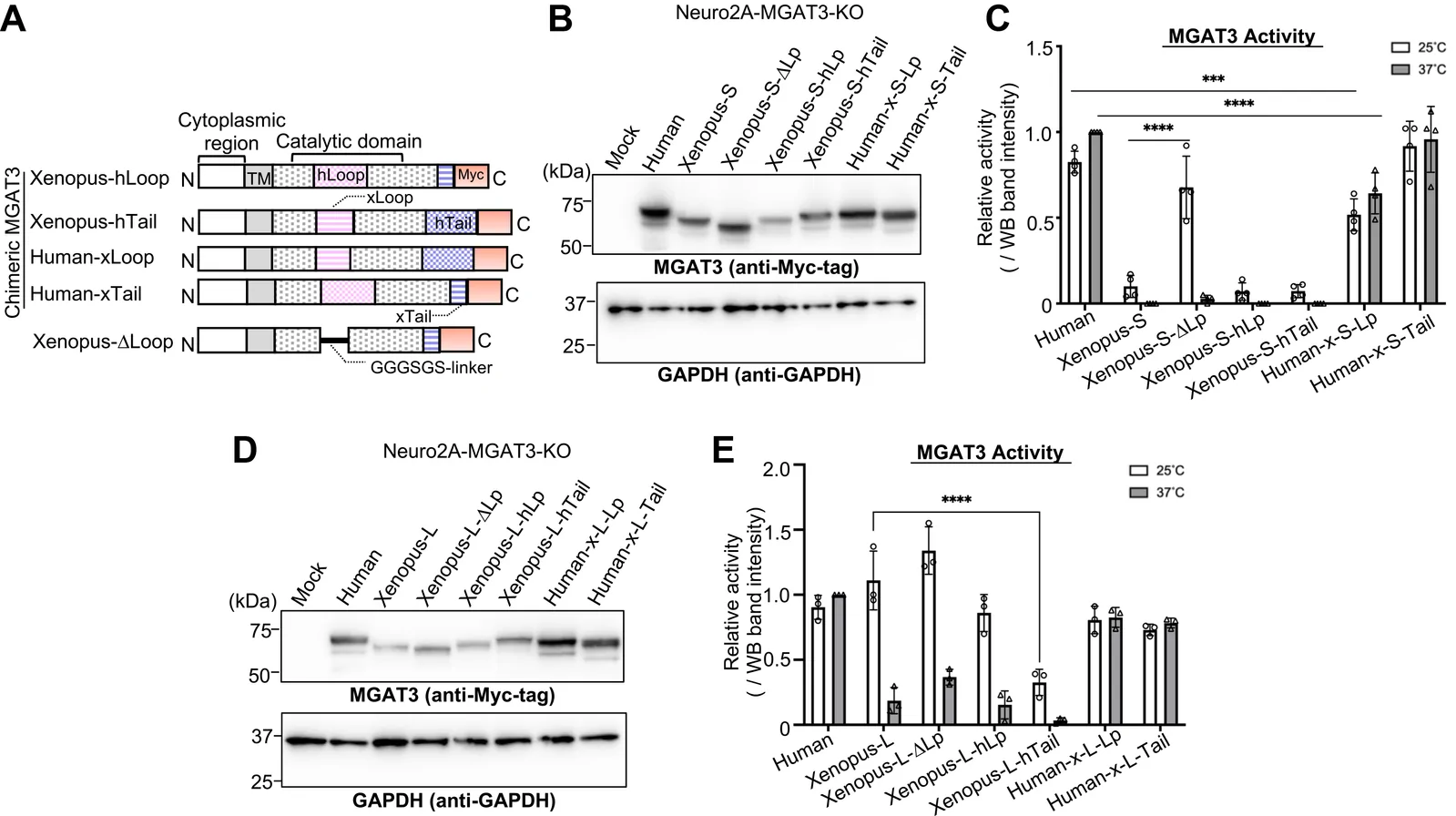
**Enzyme activity of mutants in which human and *Xenopus* MGAT3 had been swapped**. *A*, plasmid constructs used for the expression of chimeric MGAT3s. *B*, the Myc-tagged human MGAT3, *Xenopus* MGAT3-S and loop deletion mutant (xMGAT3-S-ΔLp), *Xenopus* MGAT3-S substituted with the human Loop (xMGAT3-S-hLP) or Tail (xMGAT3-S-hTail) region, and human-MGAT3 substituted with the *Xenopus*-S Loop (hMGAT3-x-S-Lp) or Tail (hMGAT3-x-S-Tail) region were expressed in Neuro2A-MGAT3-KO cells. The empty vector (pcDNA6-mycHisA) was transfected into Neuro2A-MGAT3-KO cells as a control (*Mock*). Proteins in the cell lysates were blotted with anti-Myc-tag (*upper*) and anti-GAPDH (*lower*) antibodies. *C*, the activity of MGAT3s was calculated according to the peak areas shown in Figure S12, *A* and *B* and divided by the corresponding signal intensity of MGAT3 shown in (*B*). The activity relative to that of human MGAT3-WT at 37 °C is shown (n = 4, mean ± SD, ∗∗∗*p* < 0.001, *∗∗∗∗p* < 0.0001, one-way ANOVA with Tukey’s test). *D*, the Myc-tagged human MGAT3, *Xenopus* MGAT3-L and Loop deletion mutant (xMGAT3-L-ΔLp), *Xenopus*-MGAT3-L substituted with the human Loop (xMGAT3-L-hLp) or Tail (xMGAT3-L-hTail) region, and human-MGAT3 substituted with the *Xenopus* L Loop (hMGAT3-x-L-Lp) or Tail (hMGAT3-x-L-Tail) region were expressed in Neuro2A-MGAT3-KO cells. The empty vector (pcDNA6-mycHisA) was transfected into Neuro2A-MGAT3-KO cells as a control (Mock). Proteins in the cell lysates were blotted with anti-Myc-tag (*upper*) and anti-GAPDH (*lower)* antibodies. *E*, the activity of MGAT3s was calculated according to the peak areas shown in Figure S12, *C* and *D* and divided by the corresponding signal intensity of MGAT3 shown in (*D*). The activity relative to that of human MGAT3-WT at 37 °C is shown (n = 3, mean ± SD, *∗∗∗∗p <* 0.0001, one-way ANOVA with Tukey’s test).

### Phylogenetic analysis of MGAT3

To gain greater insight into the species-specific temperature dependence of MGAT3 and to investigate the evolutionary relationships of different MGAT3s, phylogenetic analysis was performed. We collected 99 amino acid sequences of MGAT3 from various species (Fig. S13 and Table S2) and generated a phylogenetic tree using iTOL (31) (Fig. S14). To clarify the evolutionary relationships between MGAT3 sequences, another phylogenetic tree was created using 55 representative sequences according to the major taxonomic groups, including mammals, sauropsids, amphibians, teleosts, and invertebrates (Fig. 5*A*, right). Mammalian and sauropsid MGAT3s formed closely related clusters, whereas amphibian MGAT3s, including *Xenopus* homologs, constituted a distinct clade. Teleost MGAT3 sequences showed a more complex distribution, and invertebrate MGAT3-related sequences were positioned separately from vertebrate sequences, reflecting their greater evolutionary divergence (Fig. 5*A*, right, and S14). Sequence Logo analysis further revealed that conservation of the Loop region was higher within mammal and sauropsid MGAT3s, although several positions displayed residue preferences (Figs. 5*A*, left upper, and S15). The Loop region in amphibian MGAT3s displayed poor conservation, especially in the central positions (Figs. 5*A*, left middle, and S15). In teleost MGAT3s, the Loop sequences exhibited higher divergence between the two groups (Figs. 5*A*, left below, and S15).

**Figure 5:**
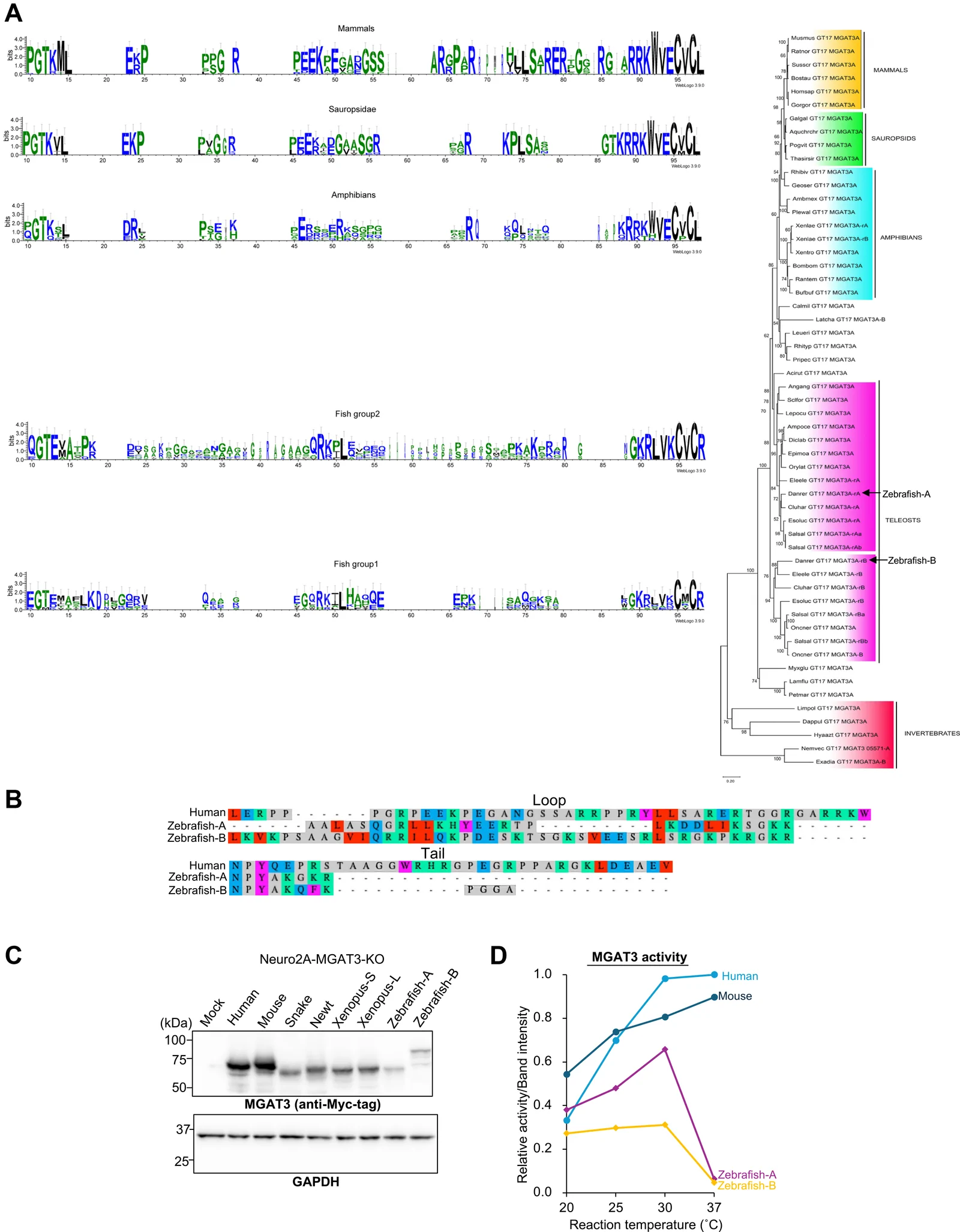
**Phylogenetic analysis of the MGAT3 sequences derived from various species**. *A*, evolutionary relationships between MGAT3 sequences and the Weblogos of the Loop region. The 99 MGAT3-related sequences identified (Table S2) were aligned using ClustalW (Fig. S13). A graphical representation of amino acid residue conservation at each position of the multiple alignment of the Loop region was obtained for each main vertebrate branch (Mammals, Sauropsidae, Amphibian, fish group 1, and fish group 2) using the Seq2Logo tool (55) and is provided on the *left side* of the figure. The overall height of the stacks indicates the sequence conservation at a given position, while the height of symbols within the stack indicates the relative frequency of each amino acid at that position (Fig. S15). On the *right side*, the evolutionary history of 55 MGAT3-related sequences was inferred using the Maximum Likelihood (ML) method based on the JTT matrix-based model (56). The tree with the highest log likelihood (−10622.83) is shown. Initial tree(s) for the heuristic search were obtained automatically by applying Neighbor-Join and BioNJ algorithms to a matrix of pairwise distances estimated using a JTT model and then selecting the topology with superior log likelihood value. A discrete Gamma distribution was used to model evolutionary rate differences among sites [5 categories (+G, parameter = 0.7066)]. The tree is drawn to scale, with branch lengths measured in the number of substitutions per site. The percentages of replicate trees in which the associated taxa clustered together in the bootstrap test (50 replicates) are shown next to the branches (57). The final dataset comprised 333 positions. Evolutionary analyses were conducted using MEGA7 (54). The major metazoan branches are highlighted with colored backgrounds (*yellow*: mammals; *green*: sauropsidae; *blue*: amphibians; *pink*: teleosts; *red*: invertebrates). Bootstrap support values of more than 50% are indicated. The 55 MGAT3 sequences were from *Mus musculus* (Musmus), *Rattus norvegicus* (Ratnor), *Sus scrofa* (Susscr), *Bos taurus* (Bostau), *Homo sapiens* (Homsap), *Gorilla gorilla* (Gorgor), *Gallus gallus* (Galgal), *Aquila chrysaetos chrysaetos* (Aquchrchr), *Pogona vitticeps* (Pogvit), *Thamnophis sirtalis sirtalis* (Thasirsir), *Rhinatrema bivittatum* (Rhibiv), *Geotrypetes seraphini* (Geoser), *Ambystoma mexicanum* (Ambmex), *Pleurodeles waltl* (Plewal), *Xenopus laevis* (Xenlae), *Xenopus tropicalis* (Xentro), *Bombina bombina* (Bombom), *Rana temporaria* (Rantem), *Bufo bufo* (Bufbuf), *Callorhinchus milii* (Calmil), *Leucoraja erinacea* (Leueri), *Rhincodon typus* (Rhityp), *Pristis pectinate* (Pripec), *Acipenser ruthenus* (*A. ruthenus*), *Anguilla anguilla* (Angang), *Scleropages formosus* (Sclfor), *Lepisosteus oculatus* (Lepocu), *Amphiprion ocellaris* (Ampoce), *Dicentrarchus labrax* (Diclab), *Epinephelus moara* (Epimoa), *Oryzias latipes* (Orylat), *Electrophorus electricus* (Eleele), *Danio rerio* (Danrer), *Clupea harengus* (Cluhar), *Esox lucius* (Esoluc), *Salmo salar* (Salsal), *Oncorhynchus nerka* (Oncner), *Myxine glutinosa* (Myxglu), *Lampetra fluviatilis* (Lamflu), *Petromyzon marinus* (Petmar), *Limulus polyphemus* (Limpol), *Daphnia pulex* (Dappul), *Hyalella Azteca* (Hyaazt), *Nematostella vectensis* (Nemvec), *Exaiptasia diaphana* (Exadia). *B*, multiple sequence alignment of the Loop and Tail regions among human-MGAT3, and zebrafish-MGAT3A and -MGAT3B. *C*, the Myc-tagged human-, mouse-, snake-, and newt-MGAT3, and Myc-tagged *Xenopus*-MGAT3-S and -L, and zebrafish-MGAT3A and -MGAT3B were expressed in Neuro2A-MGAT3-KO cells. The empty vector (pcDNA6-mycHisA) was transfected into Neuro2A-MGAT3-KO cells as a control (*Mock*). Cell lysates were prepared after 48 h of transfection, and the cellular proteins were blotted with anti-Myc-tag (*upper*) and anti-GAPDH (*lower*) antibodies. *D*, activity of MGAT3 was calculated according to the peak areas shown in Figure S17 and divided by the corresponding signal intensity of MGAT3 shown in (*C*). Activity relative to that of human-MGAT3-WT at 37 °C (1.0) is shown.

To investigate the temperature dependence of teleost MGAT3, we expressed zebrafish MGAT3A and MGAT3B, both of which possess distinct Loop sequences from human MGAT3 and a short Tail sequence (Figs. 5*B* and S16), and MGAT3s from other vertebrates in Neuro2A-MGAT3-KO cells. Using the cell lysates, we performed an MGAT3 activity assay toward the oligosaccharide substrate at different temperatures (Fig. S17). The *in vitro* activities of zebrafish MGAT3A and MGAT3B both reached a peak at 30 °C and sharply dropped at 37 °C (Fig. 5*D*), showing a temperature dependence as *Xenopus* and human MGAT3s did. These data further suggest that the thermal stability and temperature dependence of MGAT3 change during vertebrate evolution.

## Discussion

In this study, we reveal that the lengths and sequences of two disordered regions (Loop and Tail) in MGAT3 are highly divergent among species. Furthermore, we show that the specific activity, thermostability, and temperature dependence of MGAT3 are variable among species, whereas the structure of the catalytic site is conserved. Considering the broad negative impacts of bisecting GlcNAc on *N*-glycan maturation, the species-specific differences in MGAT3 activity have a great influence on shaping *N*-glycan profiles in certain species. Although the mechanisms responsible for the divergence in MGAT3 activity and temperature dependence remain unclear, our findings provide insight into how the structure and activity of MGAT3 have adapted during evolution. Disordered flexible Loop regions have also been reported in other glycosyltransferases, which change their structures upon substrate binding and play crucial roles in substrate recognition and product release (32, 33, 34). Further studies are now needed to elucidate the structure of the MGAT3–substrate complex and clarify the precise roles of the non-conserved Loop and Tail regions in various species.

Regarding the role of the Loop region in MGAT3, the *Xenopus* MGAT3-ΔLoop mutant displayed higher activity than the WT *Xenopus* enzyme (Fig. 4*C*). This was consistent with our previous findings with the human MGAT3-ΔLoop mutant (23), raising the possibility that the Loop region commonly suppresses the catalytic activity of MGAT3. Moreover, chimeric *Xenopus* MGAT3 bearing human Loop did not show catalytic activity at 37 °C (Figure 4, *C* and *E*), suggesting that Loop does not solely determine the temperature dependence of MGAT3. Notably, there is one human-specific *N*-glycosylation site (N141) in the Loop region (Fig. S1). A previous study on the roles of the *N*-glycans of rat MGAT3 found that mutation at either one of the glycosylation sites resulted in decreased activity and mislocalization compared with the WT enzyme (35), indicating that other highly conserved *N*-glycans are critically required for enzyme function. In humans, acquiring an additional N141 glycan in the Loop region might have positively contributed to its role in the regulation of catalytic activity.

Our findings reveal that snake, newt, *Xenopus*, and zebrafish MGAT3s all contain shorter Tail sequences than mammalian MGAT3s (Figs. 1, *C*, *H*, and 5*B*). Moreover, our previous study showed that the human MGAT3-ΔTail mutant displays weaker activity and faster degradation than WT, probably because of a folding defect (23). These findings raise the possibility that a shortened Tail sequence may contribute to weak thermostability. However, replacing the human Tail region with the equivalent region from *Xenopus*, which is the shortest Tail region (only 2 amino acids) among the species tested, barely affected the activity of human MGAT3 (Fig. 4, *C* and *E*). Similarly, adding the long human Tail region to *Xenopus* MGAT3s did not change their preference for a low temperature. These results indicate that the Tail region is not the sole determinant of temperature dependence and that a two-amino-acid Tail sequence (Ser-Ser) is sufficient to generate an active human MGAT3.

Unexpectedly, we detected a high level of *N*-glycans bearing bisecting GlcNAc in the *Xenopus* brain by LC-MS *N*-glycomics (Fig. 2, *C* and *D* and Table S1), indicating that *Xenopus* MGAT3s actively synthesize bisecting GlcNAc *in vivo* despite their lower thermostability than mammalian enzymes. This suggests that native conditions are required for *Xenopus* MGAT3, including a lower temperature and acceptor glycoproteins in *Xenopus*. Although the presence of bisecting GlcNAc in *X. laevis* had previously been reported following the E4-PHA staining of testis (36), our analysis unambiguously detected *N*-glycans bearing bisecting GlcNAc in *X. laevis* tissue (Fig. 2, *C* and *D*). Moreover, our data showed that the percentage and structural profile of bisected *N*-glycans in *Xenopus* brain are similar to those in mouse brain (17), suggesting that the enzymatic machinery for bisected *N*-glycans is at least partially conserved between *Xenopus* and mammals. To understand the biosynthesis and functions of bisecting GlcNAc in greater detail, investigations of glycoproteins modified with bisecting GlcNAc in *Xenopus* and of the redundancy and independence of two *Xenopus* MGAT3 enzymes *in vivo*, are necessary in the future.

Our cross-species enzyme assays demonstrated that MGAT3 has distinct temperature dependence among species (Figs. 3*A*, 5*D*, S9, *B–G*, and S17). In particular, *Xenopus* MGAT3 prefers a lower temperature (20 °C–25 °C) than mammalian MGAT3. However, *Xenopus* glycosyltransferases that function at higher temperatures have been reported; for example, optimal temperatures for *Xenopus* GALNT6 and GALNT16 were reported to be over 40 °C (37), and a hyaluronan synthase DG42 (HAS1) maintained its activity at temperatures ranging from 30 °C to 40 °C (38), indicating that the thermostability and temperature dependence of *Xenopus* glycosyltransferases may differ depending on the enzyme. In addition, a low optimal temperature has been reported for fish glycosyltransferases (*e.g.*, salmonid ST8SIA4, 27 °C) (39). Although fish and frog enzymes generally have lower optimal temperatures than human enzymes to suit their external environment (40), optimal temperatures vary depending on the enzyme and their optimal activity should therefore be carefully analyzed.

In addition to the temperature dependence, human and *Xenopus* MGAT3 exhibited different properties. The lower yield of recombinant soluble form of *Xenopus* MGAT3s at the higher temperature (37 °C) than the lower one (30 °C) could be due to less thermostability, defects in secretion, or accelerated degradation (Fig. 3*C*). It was reported that control of culture temperature and duration had complicated effects on the yield and activity of a recombinant soluble bacterial glucosyltransferase (41), and that heat stress in mammalian cells promotes proteasomal degradation of the Golgi glycosyltransferase GCNT3 (C2GnT-M) (42). These reports showed the multiple possible effects of high temperature on glycosyltransferase functions in cells. We also showed that *Xenopus* MGAT3 showed greater tolerance to various divalent cations, such as Mg^2+^ and Ca^2+^, compared with human MGAT3 (Fig. S11*B*). This suggested that ectothermic animals have evolved MGAT3 with a broader permissive range for cations, potentially reflecting adaptation to variable physicochemical conditions in a cold-blooded intracellular environment. Identifying the sequence or amino acids enabling such broader metal tolerance in *Xenopus* MGAT3 is an important issue.

Our phylogenetic analysis showed that MGAT3 orthologs are broadly maintained across vertebrates (Fig. 5*A*, right), while lineage-dependent sequence divergence was evident particularly in the non-conserved Loop and Tail regions (Figs. 5*A* and S15). Our results suggested that the two disordered regions may have distinct roles in evolution. The Loop region shows substantial sequence variation among MGAT3 orthologs (Figs. 5*A* and S15), indicating that it may serve as an evolutionarily flexible regulatory element that tunes catalytic activity in a species-specific manner. In contrast, deletion of the Tail region reduced protein stability in human MGAT3 (23), indicating that this region may be related to maintaining the structural integrity of MGAT3. These proposed roles remain to be verified through further analyses using additional orthologs, chimeric proteins, and structural approaches. Recently, we performed phylogenetic analysis of another *N*-glycan branching enzyme MGAT4 (19, 43) and found that MGAT4 has undergone greater evolutionally divergence due to several gene duplication events (8). We identified MGAT4 genes in vertebrates (*mgat4a* to *mgat4g*) with different acceptor specificities and activities, and some no longer producing functional gene product in humans because of pseudogenization and inactivating mutations (8), contributing to the presence of the species-specific non-human *N*-glycan branch (44). Our present findings provide further insight into the species-dependent differences in *N*-glycan branching.

In conclusion, our study reveals the species-dependent non-conserved sequences and temperature dependence of MGAT3. Although these findings contribute to understanding the catalytic mechanism of MGAT3, it remains unclear how the non-conserved Loop and Tail regions affect catalytic activity and what determines the species-specific temperature dependence of MGAT3. These questions may be addressed by elucidating the 3D structure of the MGAT3–substrate complex, which may eventually lead to the development of novel therapeutic strategies against MGAT3-associated diseases.

## Experimental procedures

### Reagents

Listed antibodies and lectins were used: anti-Myc-tag (mouse, clone 4A6, Merck Millipore; 05–724), anti-GAPDH (mouse, clone 6C5, Merck Millipore; G8795), horseradish peroxidase (HRP)-conjugated anti-mouse IgG (Cytiva; NA931-1 Ml), E4-PHA (J-Chemical; J111). E4-PHA was conjugated with HRP using Peroxidase Labeling Kit-NH2 (Dojindo; LK11).

### Plasmid construction

Synthetic oligonucleotides and cDNAs for plasmid construction were listed in Table S3. The plasmids for human MGAT3-WT, -ΔLoop and -ΔTail (pcDNA6-myc-HisA/human MGAT3-WT/ΔLoop/ΔTail-2 × Myc) were constructed in our previous study (23). The cDNAs encoding MGAT3 of mouse, snake, *Xenopus* (S and L), newt, and zebrafish (A and B) were artificially synthesized with codon optimization for expression in mouse cells as listed in Table S3. Using these cDNAs as templates, DNA fragments were amplified and ligated into EcoRI/XhoI sites of pcDNA6-Myc-HisA by Gibson assembly to obtain plasmids expressing MGAT3s with a Myc-tag. For the plasmids for *Xenopus* MGAT3-S/L-ΔLoop, using the respective WT-Myc version of plasmids as template, two cDNA fragments were amplified so that the GGGSGS linker sequence was inserted instead of Loop. The objective DNA fragments were ligated into EcoRI/XhoI sites of pcDNA6-myc-HisA by Gibson assembly. For Loop-swapping mutants between human and *Xenopus*, two DNA fragments were amplified using pcDNA6-myc-HisA-human/*Xenopus*-S/L-ΔLoop as the template, and these amplified fragments together with the annealed oligonucleotides encoding *Xenopus* or human Loop were inserted into EcoRI/XhoI sites of pcDNA6-myc-HisA by Gibson assembly. For chimeric human MGAT3 with *Xenopus* Tail, the DNA fragment until P505 was amplified using pcDNA6-myc-HisA-human MGAT3-2 × Myc as template with the primer containing *Xenopus* Tail (SS) and ligated into EcoRI/XhoI sites of pcDNA-myc-HisA by Gibson assembly. For chimeric *Xenopus* MGAT3 with human Tail, the DNA fragment until Y498/Y500 in *Xenopus*-S/L was amplified using the respective WT-Myc version of plasmids as templates. The amplified fragment together with the annealed oligonucleotides encoding human Tail were ligated into EcoRI/XhoI site of pcDNA-myc-HisA by Gibson assembly. The plasmid for pcDNA-IHT/human MGAT3-WT was constructed in our previous study (23). The DNA fragments encoding the truncated luminal domain of *Xenopus* MGAT3-S (R63 to S502) and -L (R63 to S500) were amplified by PCR using the respective WT-Myc versions of the plasmids as templates. The resultant fragments were ligated into EcoRI/XhoI sites of pcDNA-IHT by Gibson assembly.

### Cell culture

Neuro2A-MGAT3-KO (45) and HEK293T cells (ATCC) were cultured in Dulbecco’s modified Eagle’s medium (D-MEM) containing 10% (v/v) fetal bovine serum (FBS) and 50 μg/ml kanamycin. Cells were cultured at 37 °C in a 5% CO_2_ atmosphere.

### Plasmid transfection and preparation of cell lysate

Cells were cultured on a 6-cm culture dish for 24 h to reach 60%–80% confluency. Five μg of plasmids (for 6-cm dish) were transfected into cells using Lipofectamine 3000 reagent (Thermo Fisher Scientific) according to the manufacturer’s protocol. After 48 h, cells were washed with PBS and collected by scraping, followed by centrifugation at 470*g* for 5 min. After washing with PBS, cells were centrifuged again at 13,800*g* for 2 min. The cells were then lysed in lysis buffer [PBS, 0.5% Nonidet P-40, Protease Inhibitor Cocktail Set V (EDTA free) (Fujifilm)] by sonication. Protein concentration was measured using Pierce BCA Protein Assay Kit (Thermo Fisher Scientific) according to the manufacturer’s protocol. The cell lysates were mixed with Laemmli 5 × SDS sample buffer and heated at 95 °C for 5 min.

For purification of soluble MGAT3, cells were cultured on a 15-cm culture dish, and plasmids were transfected using polyethyleneimine MAX (Polysciences) as described below (see “Purification of recombinant enzymes”).

### Western and lectin blotting, and Coomassie brilliant blue (CBB) staining

The same amount of protein was applied to each lane of 5%–20% sodium dodecyl sulfate-polyacrylamide (SDS-PAGE) gels and separated by electrophoresis. CBB staining of proteins was performed using GelCode Blue Safe Protein Stain (Thermo Fisher Scientific). For western blotting, the proteins were transferred to nitrocellulose membranes using a semi-dry blotter. The membranes were blocked with 5% (w/v) skim milk in TBS containing 0.1% (v/v) Tween-20 (TBS-T) for over 30 min. Then, the membranes were incubated with the primary antibody diluted with 5% skim milk in TBS-T at 4 °C overnight. The membranes were washed for 5 min with TBS-T three times and then incubated with the secondary antibody diluted with 5% skim milk in TBS-T at room temperature for 1 h. After washing three times with TBS-T and once with TBS, chemiluminescence signals were detected as described below.

For lectin blotting, after transfer, membranes were blocked with 1% (w/v) bovine serum albumin (BSA) in TBS-T at room temperature for 1 h. The membranes were incubated with HRP-conjugated lectin diluted with 1% BSA in TBS-T at 4 °C overnight. The membranes were washed for 5 min with TBS-T three times and for 5 min with TBS once; chemiluminescence signals were detected as follows.

Membranes were incubated with Western Lightning Plus-ECL (PerkinElmer) for 30 s, and the chemiluminescence signals were detected using a FUSION-SOLO 7s EDGE system (Vilber Lourmat). The antibodies and lectins were diluted as follows: anti-Myc tag (1:500), anti-GAPDH (1:1000), HRP-conjugated anti-mouse IgG (1:5000), and HRP-conjugated E4-PHA (1:1000). Representative images from at least three experiments are shown in figures.

### Assay of MGAT3 activity toward oligosaccharide using high-performance liquid chromatography (HPLC)

MGAT3 activity toward an oligosaccharide substrate was measured using an HPLC method with slight modifications from a previous study (7). The fluorescence-labeled oligosaccharide, GnGnbi-PA, was used as the acceptor substrate, and cell lysates were used as the enzyme source. The reaction (10 μl in total, containing 20 mM UDP-GlcNAc and 10 μM GnGnbi-PA) was conducted at 37 °C for 3 h in reaction buffer [200 mM GlcNAc, 125 mM MES (pH 6.2), 0.5% (v/v) Triton X-100, 1 mg/ml BSA, 10 mM MnCl_2_] and stopped by heating at 95 °C for 5 min. To assay MGAT3 activity at various pH values, the reaction buffers were prepared as described above, except that 125 mM MES (pH 6.2) was replaced with 125 mM MES pH 5.4, 5.7, 6.0, 6.3, 6.6, or 6.9. In addition, to assay MGAT3 activity with various divalent cations, 10 mM MnCl_2_ in the standard reaction buffer was substituted with 1 mM MnCl_2_, 10 mM CaCl_2_, 10 mM ZnSO_4_, or 10 mM MgCl_2_, respectively. The mixture was diluted with 40 μl of water and then centrifuged at 16,000*g* for 5 min. The supernatant was analyzed by reversed-phase HPLC using an ODS column (GL Sciences, Inertsil ODS-3, 4.6 × 250 mm). HPLC analysis was conducted by isocratic elution with buffer A [20 mM ammonium acetate buffer (pH 4.0)] and B (1% 1-butanol in buffer A) mixed at a 4:1 ratio.

### Animal experiments

All animal experiments in this study followed the guidelines for animal experiments of Gifu University and were approved by the institutional Animal Experiment Committee of Gifu University (approval ID: AG-P-C-20250025 and AG-P-N-20250081). All mice were from a C57BL/6N genetic background, and *Mgat3*-deficient (*Mgat3*^−/−^) mice were generously provided by Dr Jamey D. Marth (University of California, Santa Barbara) (46). The mice were housed with fewer than five individuals for each cage under the following conditions: 23 ± 3 °C, 55% ± 15% humidity, and 12 h light:12 h dark. All *X. laevis* (male adult) were purchased from Hamamatsu Seibutsu Kyozai Co. (Japan). For brain and kidney collection, mice were anesthetized by isoflurane. For brain collection, frogs were anesthetized by transfer to a water tank containing 2 g/l of ethyl m-aminobenzoate methanesulfonate.

### Preparation of homogenate and membrane fraction

The homogenate was prepared for *N*-glycomic analysis. A *X. laevis* brain was homogenized in 400 μl of ultra-pure water in a BiomasherII (Fujifilm). The homogenate was rapidly frozen in liquid nitrogen and lyophilized overnight by a frozen vacuum dryer (LABCONKO, Fz-2.5, Asahi Life Science).

The membrane fraction was prepared for biochemical experiments. The adult (13–65-week-old) mice brains and kidneys were homogenized in seven volumes of homogenization buffer [(50 mM Tris-HCl, pH 7.4, 0.15 M NaCl, Protease Inhibitor Cocktail Set V (EDTA free) (Fujifilm)] in a Potter-Elvehjem-type tissue grinder. *X. laevis* brains were homogenized in 400 μl of the homogenization buffer in BiomasherII (Fujifilm). The homogenate was centrifuged at 1000*g* for 10 min at 4 °C to remove nuclei and unbroken cells. The supernatant was ultracentrifuged at 105,000*g* for 30 min at 4 °C. The resultant precipitates (membrane fraction) were lysed with lysis buffer [(50 mM Tris-HCl, pH 7.4, 0.15 M NaCl, 0.5% Nonidet P-40, Protease Inhibitor Cocktail Set V (EDTA free) (Fujifilm)] by sonication and then used for subsequent analysis.

### Glycan structure analysis

PA-labeled *N*-glycans were prepared from a *X. laevis* brain by hydrazinolysis followed by pyridylamination, as described previously (47). Briefly, 1.5 mg of lyophilized *Xenopus* brain homogenates was heated at 100°C for 12 h with 0.5 ml of anhydrous hydrazine. After removal of hydrazine by repeated evaporations, the released glycans were re-N-acetylated with acetic anhydride in a saturated sodium bicarbonate solution and subsequently passed through a Dowex 50W x 2 (H^+^) cation-exchange resin (Dow Chemicals) to remove sodium ions. Lyophilized sample was then heated at 90 °C for 60 min with 20 μl of pyridylamination reagent, followed by heating at 80 °C for 35 min after the addition of 70 μl of reducing reagent. PA-glycans were purified by phenol/chloroform extraction, gel filtration on a TSKgel Toyopearl HW-40F column (Tosoh, Tokyo, Japan), and solid-phase extraction using a graphite carbon cartridge (GL-Pak Carbograph 300 mg; GL Sciences Ltd, Tokyo, Japan).

Anion-exchange HPLC was performed on a TSKgel DEAE-5PW column (0.75 × 7.5 cm; Tosoh) using a Waters Alliance HPLC system equipped with a Waters 2475 fluorescence detector (Milford) (47).

LC-MS/MS analysis was performed using an LTQ XL linear ion trap mass spectrometer equipped with ESI and coupled to a Dionex Ultimate 3000 HPLC system (Thermo Scientific), as described previously (48). Briefly, PA-*N*-glycans were eluted with 0.2% (v/v) formic acid in water as solvent A and 20% acetonitrile in 0.2% formic acid as solvent B and were separated on a reversed-phase InertSustain AQ-C18 column (3 μm, 0.21 × 15 cm, GL Sciences Inc., Tokyo, Japan). After separation, the column eluate was split and introduced into both the fluorescence detector (FLD) and the mass spectrometer.

For glycan structure assignment, the elution times of PA-glycans in reversed-phase LC were normalized using the glucose unit scale (49) and the reversed-phase scale (50) (Table S1). Glycan structures were assigned comprehensively by comparison with the existing data, taking into account elution behavior in reversed-phase HPLC and fragmentation patterns obtained by MS/MS analysis. Quantification of PA-*N*-glycans was based on peak areas in the fluorescence chromatograms obtained by reversed-phase LC. When multiple glycans co-eluted within a single peak, the fluorescence peak area was apportioned according to the relative signal intensities in the mass spectra. Symbol nomenclature was described in accordance with the international guideline, Symbol Nomenclature for Glycans (SNFG).

### Purification of recombinant enzymes

HEK293T cells were cultured on 15-cm culture dishes to obtain soluble His-tagged human and *Xenopus* MGAT3. The cells were transfected with pcDNA-IHT/human or *Xenopus* MGAT3 using polyethyleneimine MAX when confluency reached 60%–80%. After culture for 4 to 6 h, media were replaced with Opti-MEM I. HEK293T cells transfected with the plasmid for human MGAT3 were further cultured at 37 °C for 72 h, whereas the cells transfected with the plasmids for *Xenopus* MGAT3 were further cultured at 30 °C or 37 °C for 72 h. Soluble His-tagged human and *Xenopus* MGAT3 were purified from media through a Ni^2+^-column. After applying the samples to the column, the column was washed with 10 mM phosphate buffer (pH 7.4) containing 0.5 M NaCl and 20 mM imidazole. The His-tagged proteins were eluted with 10 mM phosphate buffer (pH 7.4) containing 0.5 M NaCl and 0.5 M imidazole. The eluates were concentrated using Amicon Ultra Centrifugal Filters (10 kDa MWCO, Millipore).

### Structural representation

The atomic structures of MGAT3 from various species were generated by AlphaFold2 (51). The structural figures were drawn with PyMOL (The PyMOL Molecular Graphics System, Version 3.1.4.1 Schrödinger, LLC.).

### Thermal stability assay

The thermal shift assay was performed using Tycho NT. 6 (NanoTemper Technologies). Purified human and *Xenopus* MGAT3 in 50 mM MES buffer were loaded in glass capillaries. Intrinsic fluorescence was recorded at 330 nm and 350 nm while heating the sample from 35 to 95 °C. The ratio of fluorescence (350/330 nm) and the inflection temperatures (Ti) were calculated by Tycho NT.6.

### Sequence alignment

The amino acid sequence of human MGAT3 (NCBI accession number NP_001091740) was used as template to identify similar sequences of other vertebrate by blast search against NCBI database. The homologous sequences from various vertebrates were selected. Multiple sequence alignment (MSA) was performed by MAFFT server, and the output was visualized using ESPript3.0 (52).

### Identification of MGAT3 (GT17)-related sequences

The CAZyme predicted GT17 proteins were identified by BLAST analysis and retrieved from public databases (*e.g.*, NCBI and ENSEMBL). Sequences of human MGAT3 were used as a query for BLAST search of reference genomes (53) and identify orthologues in representative metazoan taxa.

### Phylogenetic analysis

Phylogenetic relationships were established using the Molecular Evolutionary Genetics Analysis software version 7 (MEGA 7.0.26) (54). Ninety-nine protein sequences were aligned with the MUSCLE program in MEGA 7. The multiple alignments were curated manually to retain the informative sites of 55 sequences. Phylogenetic trees were obtained using the Maximum Likelihood (ML) method and edited in Mega 7.0.26 (https://www.megasoftware.net/57) and graphically shaped with Adobe Illustrator CS6. Conservation in the loop region in each group of animals was visualized by a sequence logo, a graphical representation of conservation and variability of motifs in the MSA. These logos were generated using the Seq2Logo 2.0 program (55), where the y axis describes the amount of information in bits.

### Statistical analysis

Statistical analyses were performed using GraphPad Prism 10 software (GraphPad Software, Inc). Data are presented as mean ± SD n represents the number of independent biological replicates or the number of individual animals. The used statistical tests, multiple-comparison procedures and significance threshold are described in the figure legends.

## Data availability

Glycomic raw data for glycan structure analysis by LC-MS have been deposited in GlycoPOST (announced ID: GPST000735). All other data are contained within this manuscript.

## Supporting information

This article contains supporting information (31).

## Conflict of interest

The author is an Editorial Board Member/Editor-in-Chief/Associate Editor/Guest Editor for this journal and was not involved in the editorial review or the decision to publish this article. The authors declare the following financial interests/personal relationships which may be considered as potential competing interests: One of the authors (YK) is an Editorial Board Member for this journal but was not involved in the editorial review or the decision to publish this article.

## Supporting information

SupportingInformation

TableS1

TableS2

TableS3
