# Supplementary material for: Species-specific disordered regions and temperature dependence of bisecting GlcNAc-synthetic enzyme MGAT3
Source: J Biol Chem. 2026 Sep 23;302(11):113604. doi: 10.1016/j.jbc.2026.113604 (PMC13696804; doi:10.1016/j.jbc.2026.113604)
Supplement: Supplementary file 1 [file mmc1.pdf]

Supporting Information

Species-specific disordered regions and temperature dependence of bisecting GlcNAc-synthetic enzyme MGAT3

Wanxue Bao, Shunji Natsuka, Anne Harduin-Lepers, Masamichi Nagae, Takahiro Yamasaki, Atsushi Iwasawa, Kyoko Matsuzawa, Yasuhiko Kizuka

*Correspondence: Yasuhiko Kizuka, Ph.D., kizuka.yasuhiko.k8@f.gifu-u.ac.jp.,

This Supporting information includes:

Fig. S1–S17 (included in this PDF)

Table S1-S3 (separately submitted)

Figure S1

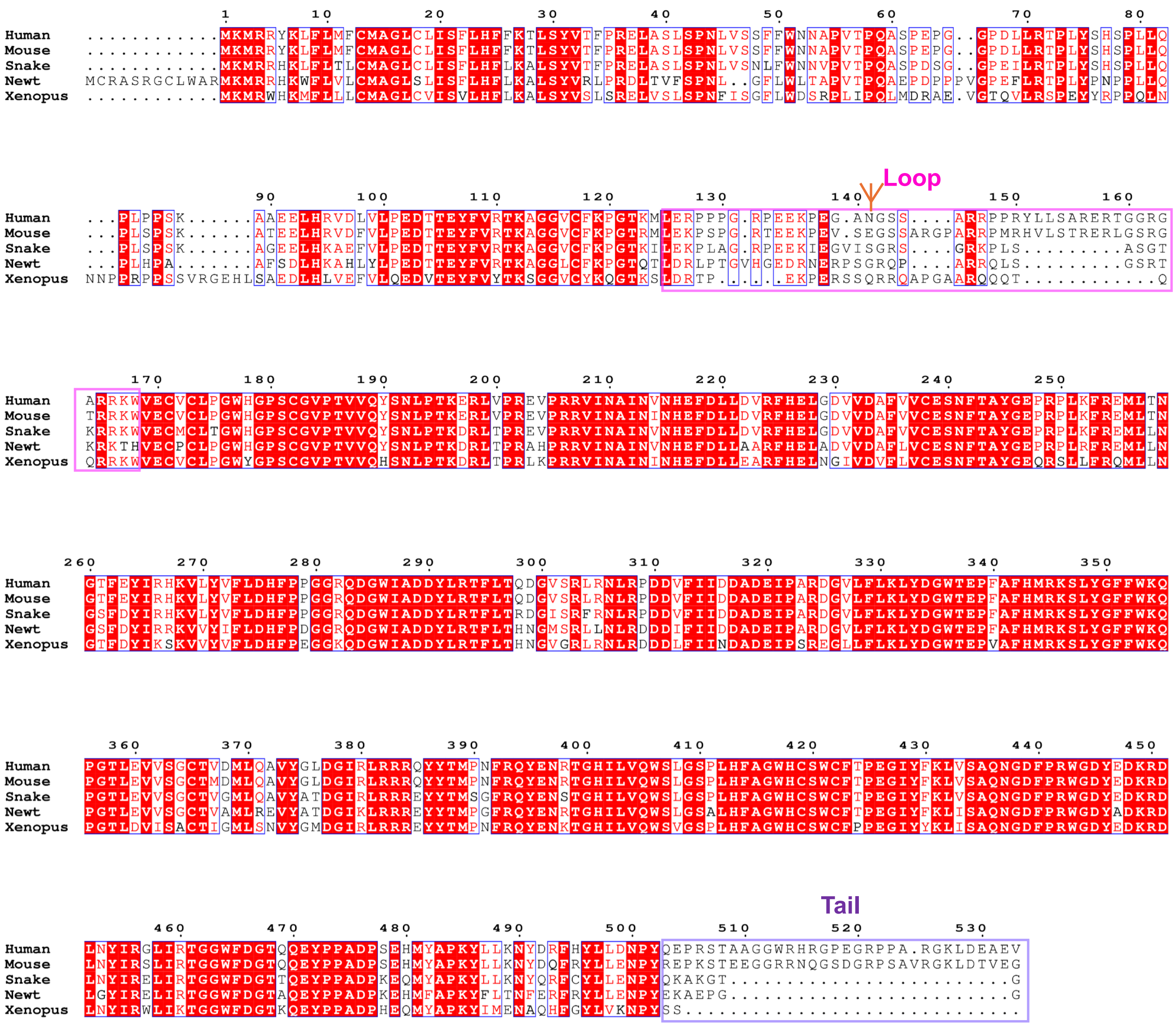

Figure S1. Sequence alignment of MGAT3 among mammals, reptiles and amphibia. The amino acid sequence of human MGAT3 (NCBI accession number NP_001091740) was used as template to identify similar sequences of other vertebrate species by blast search against NCBI database. The homologous sequences from four representative vertebrate species were selected. Mouse: *Mus musculus*, NCBI accession number NP_034925; Snake: *Thamnophis sirtalis,* NCBI accession number XP_013930020; *Xenopus*: *Xenopus laevis,* NCBI accession number XP_018116698; Newt: *Pleurodeles waltl*, NCBI accession number XP_069087394. The conserved sites were enclosed in blue, and the conserved residues were shown in red. The Loop was enclosed by magenta rectangle, and the Tail was enclosed by violet rectangle. The *N*-glycosylation site N141 in human MGAT3 was indicated by orange trident.

Figure S2

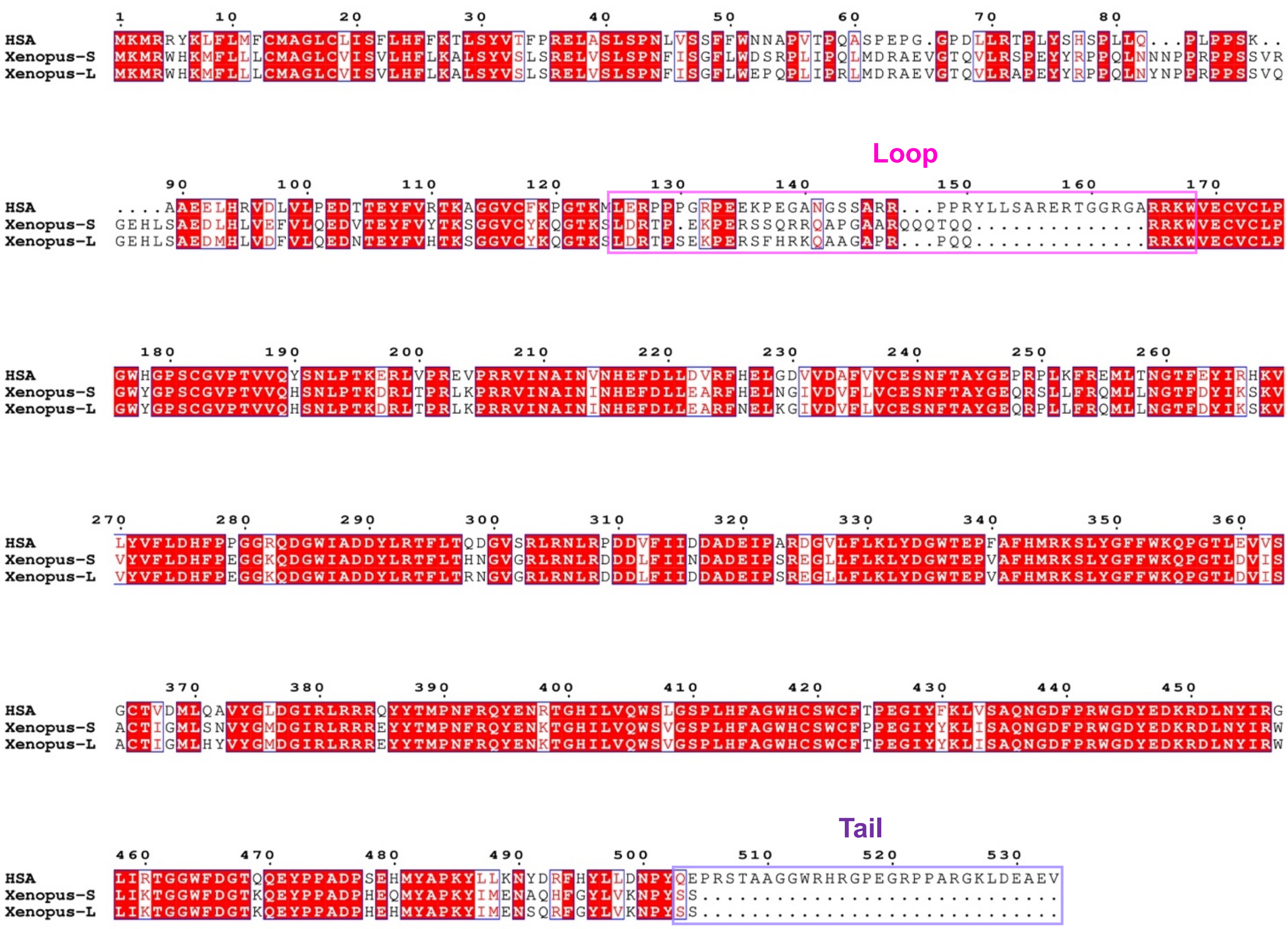

Figure S2. Sequence alignment of MGAT3 among human, *Xenopus*-L, and *Xenopus*-S. The amino acid sequence of human MGAT3 (NCBI accession number NP_001091740) was used as template. *Xenopus*-L: *Xenopus laevis*-L, NCBI accession number XP_018114325; *Xenopus*-S: *Xenopus laevis*-S, NCBI accession number XP_018116698. The conserved sites were enclosed in blue, and the conserved residues were shown in red. The Loop was enclosed by magenta rectangle, and the Tail was enclosed by violet rectangle.

Figure S3

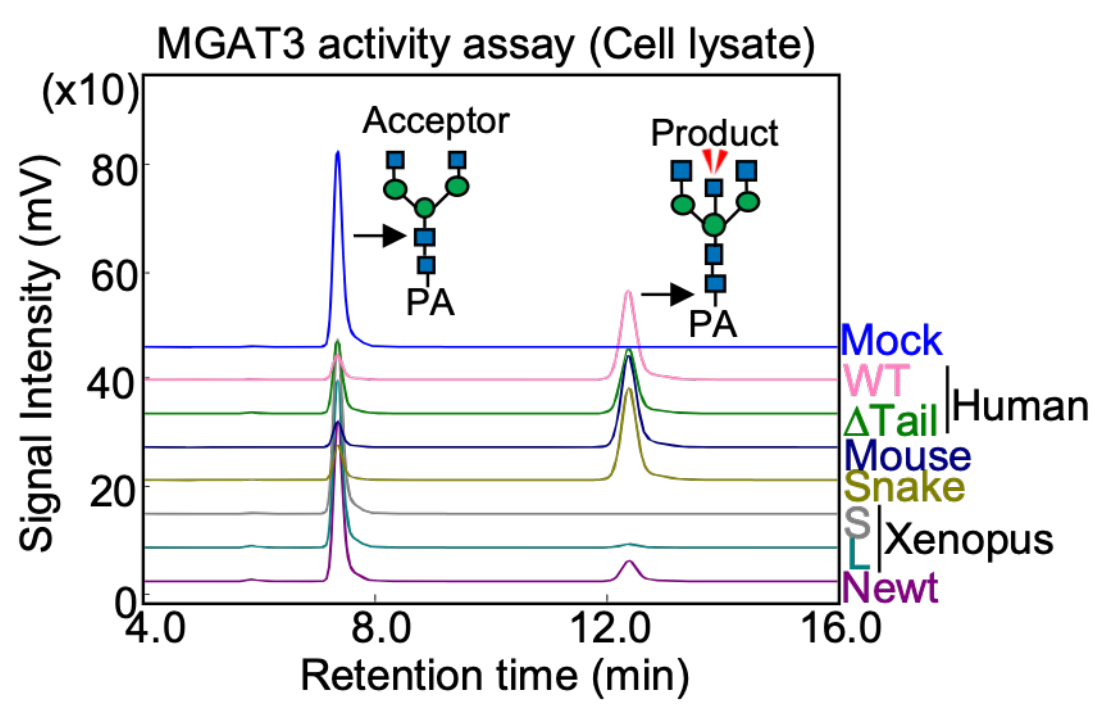

Figure S3. The HPLC-based activity assay of MGAT3 in various species. The cell lysates of Neuro2A-MGAT3-III-KO expressing MGAT3 from various species were reacted with the fluorescently labeled oligosaccharide acceptor substrate, GnGnbi-PA. The unreacted substrate and the product with bisecting GlcNAc were separated and quantified by reverse phase HPLC.

Figure S4

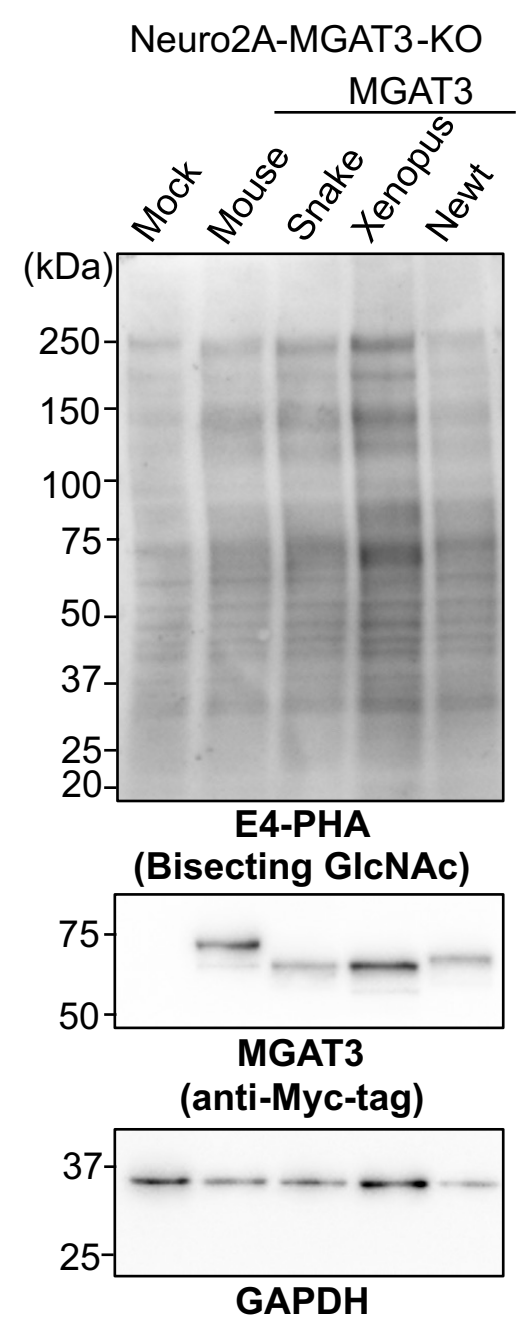

Figure S4. *In cellulo* activity of MGAT3 of various vertebrate species. Myc-tagged mouse-, snake-, *Xenopus*- and newt-MGAT3 were expressed, or the empty vector (pcDNA6-mycHisA) was transfected (Mock) in Neuro2A-MGAT3-KO cells. Cell lysates were prepared after 48 h of transfection, and the cellular proteins were blotted with E4-PHA (upper), anti-Myc-tag (middle) and anti-GAPDH (lower) antibodies.

Figure S5

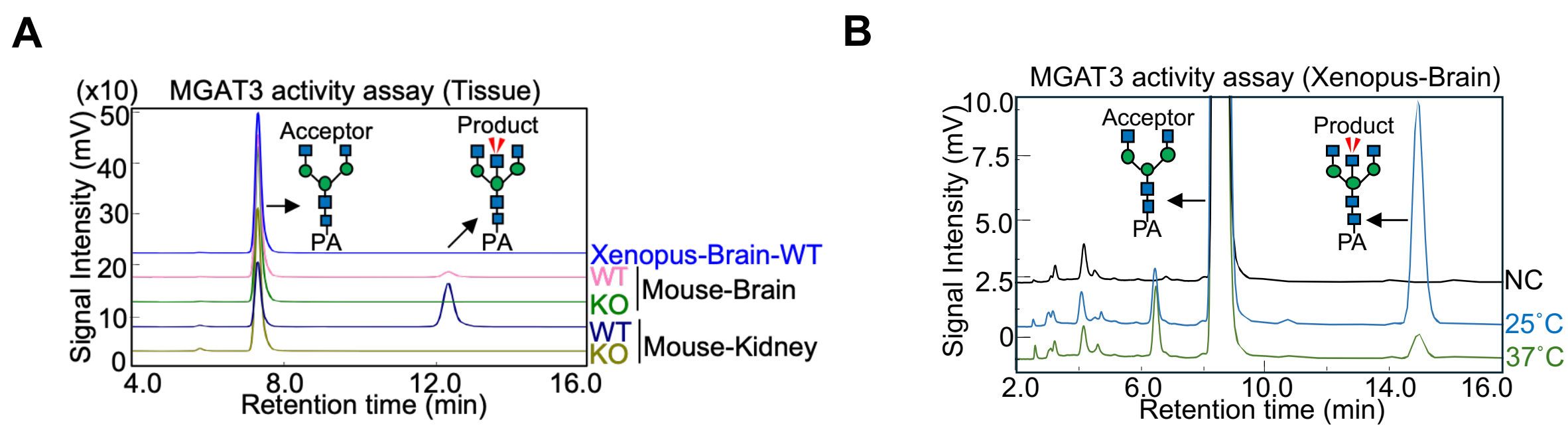

Figure S5. The HPLC-based activity assay of mouse brain, mouse kidney and *Xenopus* brain. *A*, The membrane lysates of *Xenopus* brains, and brains and kidneys of WT and *Mgat3* KO mice were reacted with the fluorescently labeled oligosaccharide acceptor substrate, GnGnbi-PA. The unreacted substrate and the product with bisecting GlcNAc were separated and quantified by reverse phase HPLC. ***B*,** The membrane lysates of *Xenopus* brains were reacted with the fluorescently labeled oligosaccharide acceptor substrate, GnGnbi-PA at 25˚C or 37˚C. The unreacted substrate and the product with bisecting GlcNAc were separated and quantified by reverse phase HPLC. NC (negative control): ultrapure water instead of membrane lysate.

Figure S6

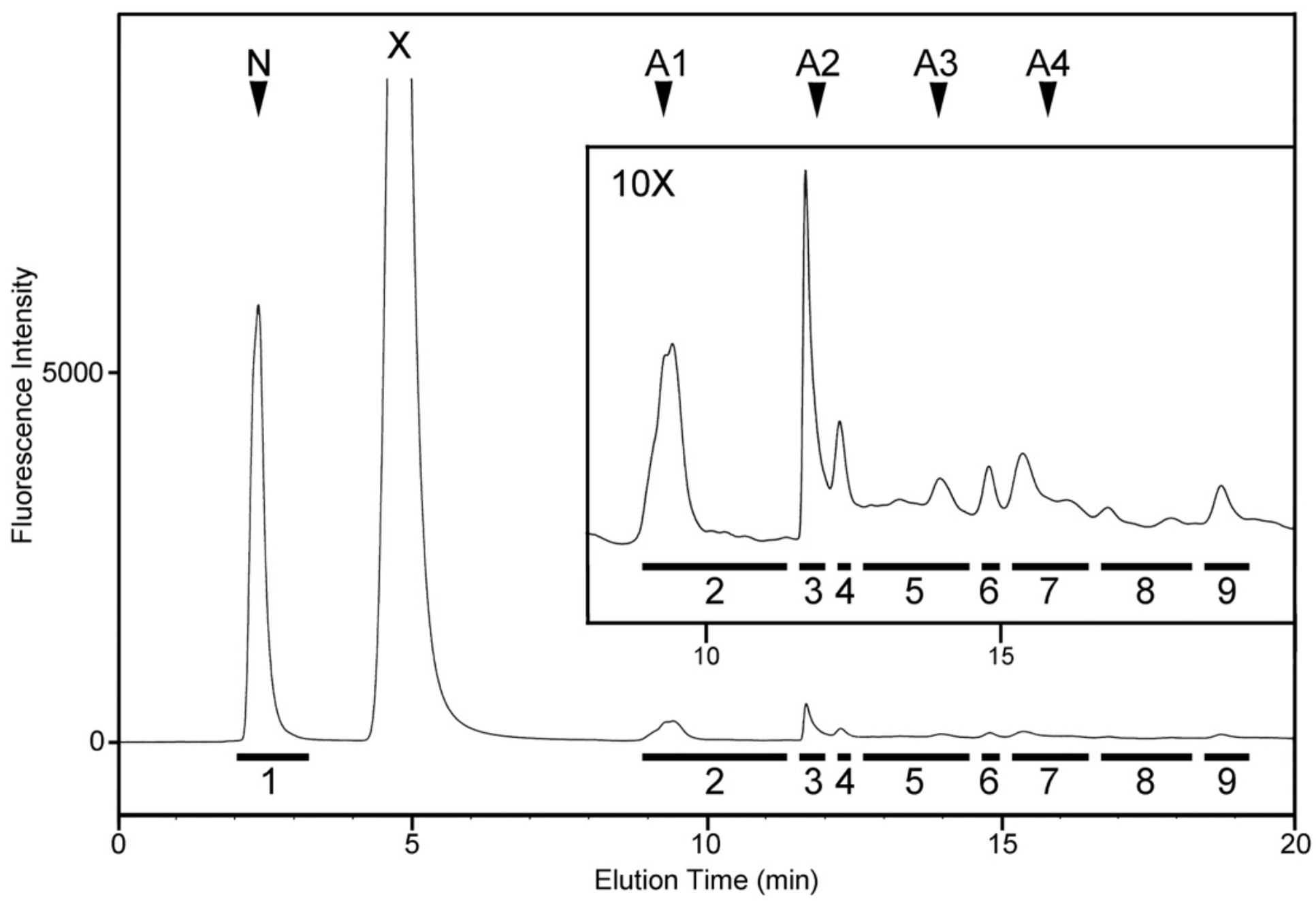

Figure S6. Anion-exchange chromatogram of PA-glycans derived from *Xenopus* brain. Fractions 1–9 were collected and subjected to further analysis. Arrowheads labeled N and A1–A4 indicate the elution positions of asialo-, monosialo-, disialo-, trisialo-, and tetrasialo-PA *N*-glycans, respectively, derived from standard human α1-acid glycoprotein.

Figure S7

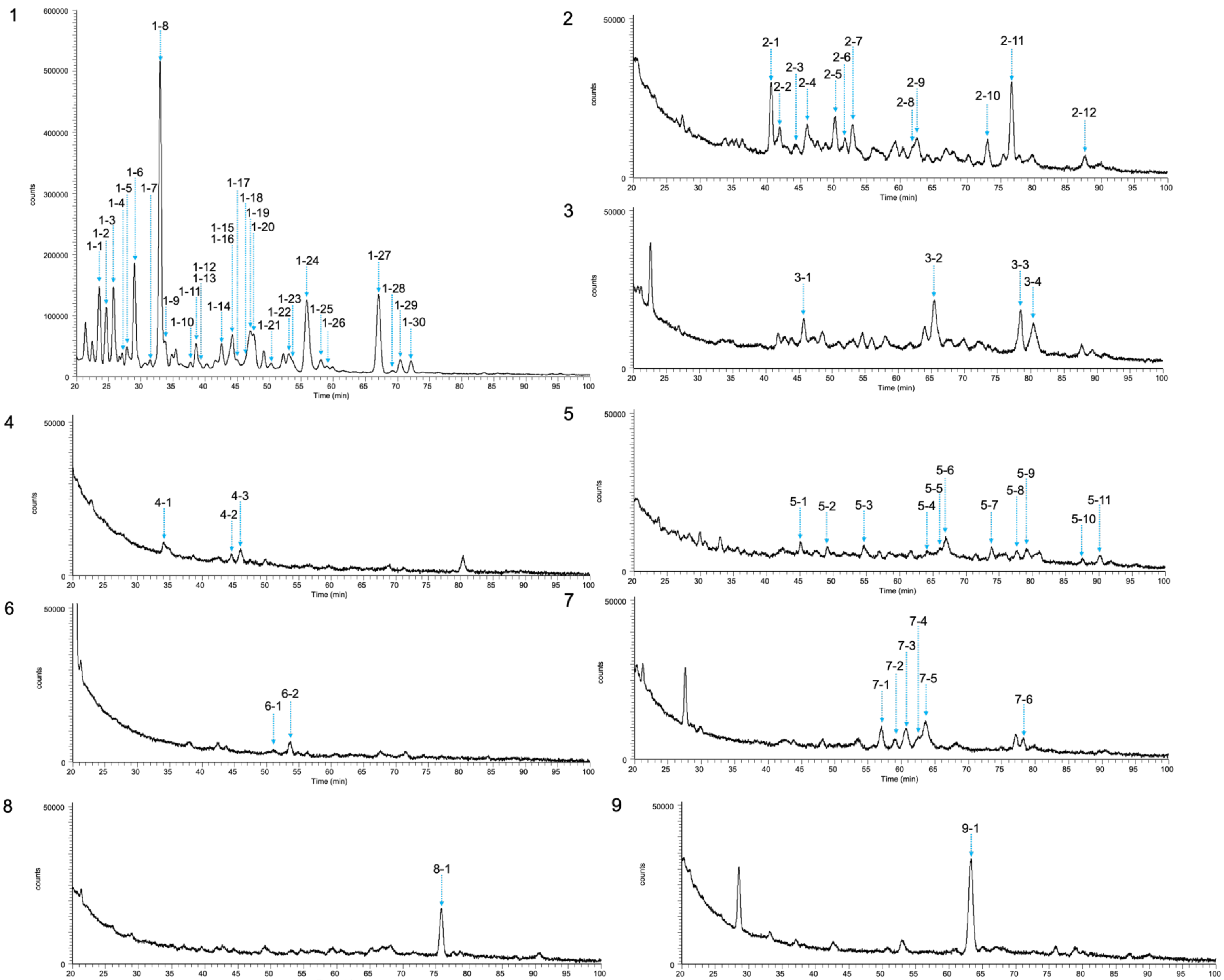

Figure S7. Reverse-phase LC separation of fractions (1~9) from anion-exchange HPLC. Reversed-phase chromatograms of fractions 1–9. The analysis results for the labeled peaks are summarized in Table S1.

Figure S8

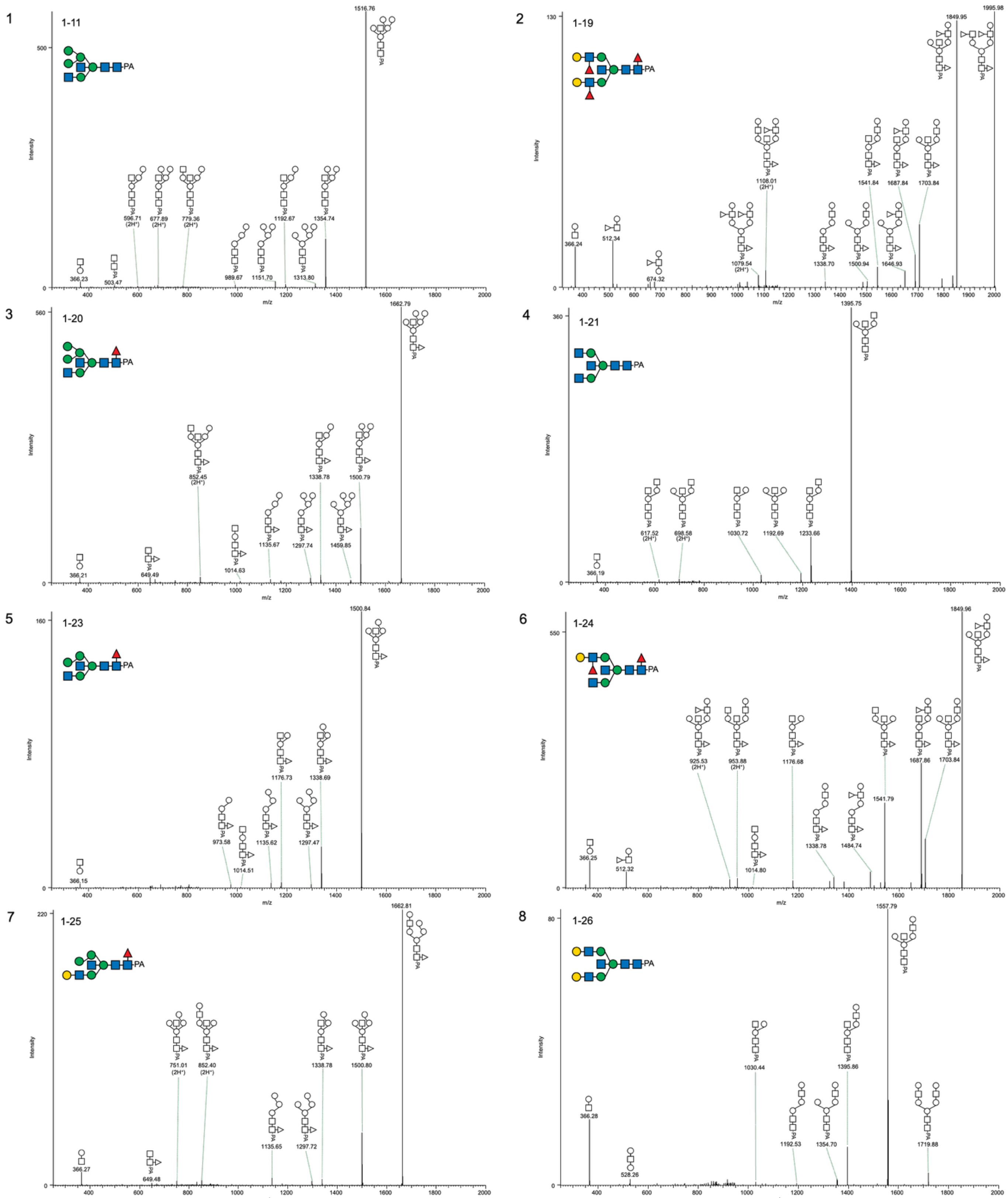
1
1-11
1516.76
596.71 (2H+)
677.89 (2H+)
779.36 (2H+)
989.67
1151.70
1192.67
1313.80
1354.74
366.23
503.47
2
1-19
1849.95
1995.98
366.24
512.34
674.32
1079.54 (2H+)
1108.01 (2H+)
1338.70
1500.94
1541.84
1646.93
1687.84
1703.84
3
1-20
1662.79
366.21
649.49
852.45 (2H+)
1014.63
1135.67
1297.74
1338.78
1459.85
1500.79
4
1-21
1395.75
366.19
617.52 (2H+)
698.58 (2H+)
1030.72
1192.69
1233.66
5
1-23
1500.84
366.15
973.58
1014.51
1135.62
1176.73
1297.47
1338.69
6
1-24
1849.96
366.25
512.32
925.53 (2H+)
953.88 (2H+)
1014.80
1176.68
1338.78
1484.74
1541.79
1687.86
1703.84
7
1-25
1662.81
366.27
649.48
751.01 (2H+)
852.40 (2H+)
1135.65
1297.72
1338.78
1500.80
8
1-26
1557.79
366.28
528.26
1030.44
1192.53
1354.70
1395.86
1719.88
Intensity
m/z
PA

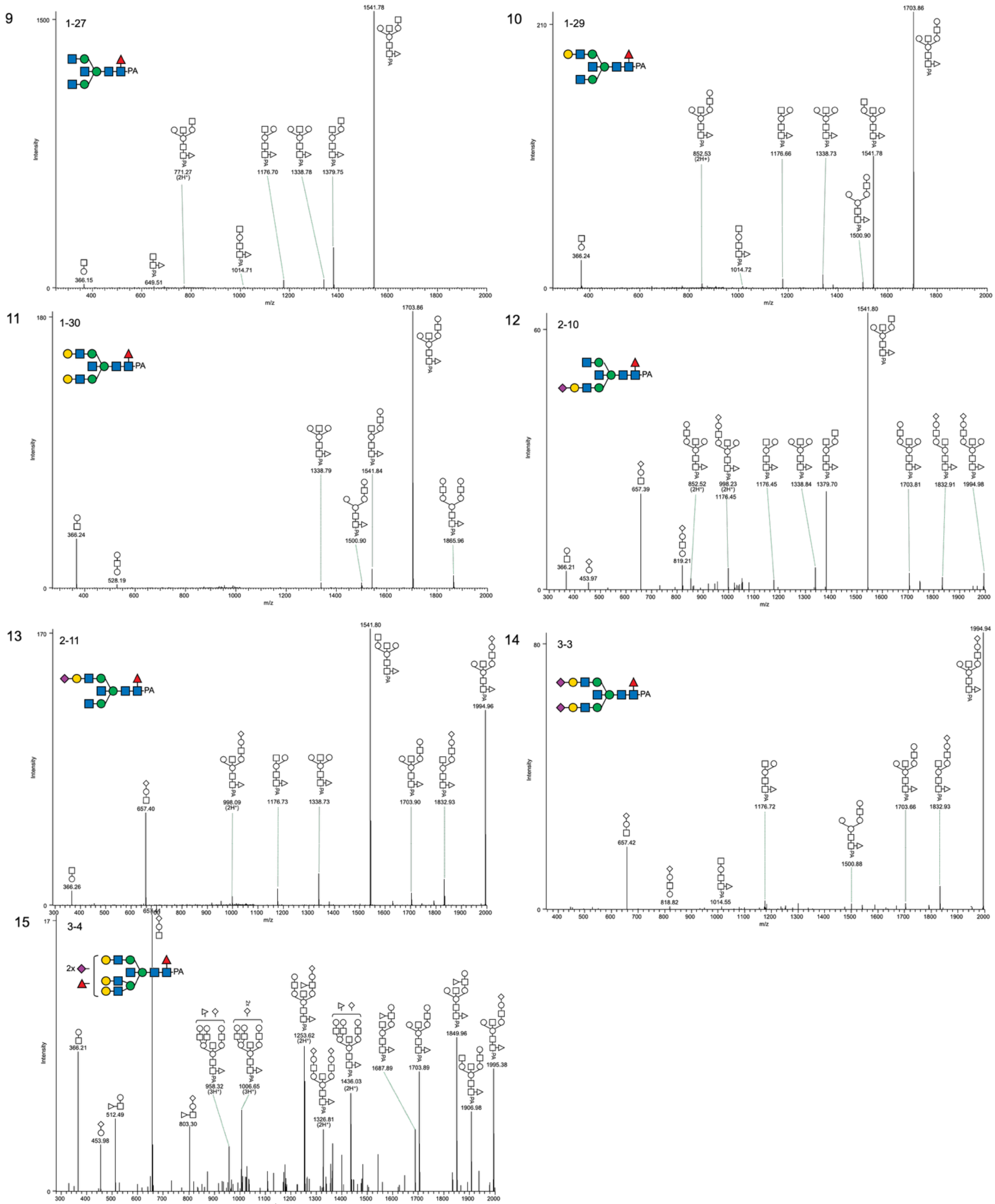

Figure S8. MS/MS spectra of glycans containing bisecting GlcNAc derived from *Xenopus* brain. The glycan numbers are listed in Table S1.

Figure S9

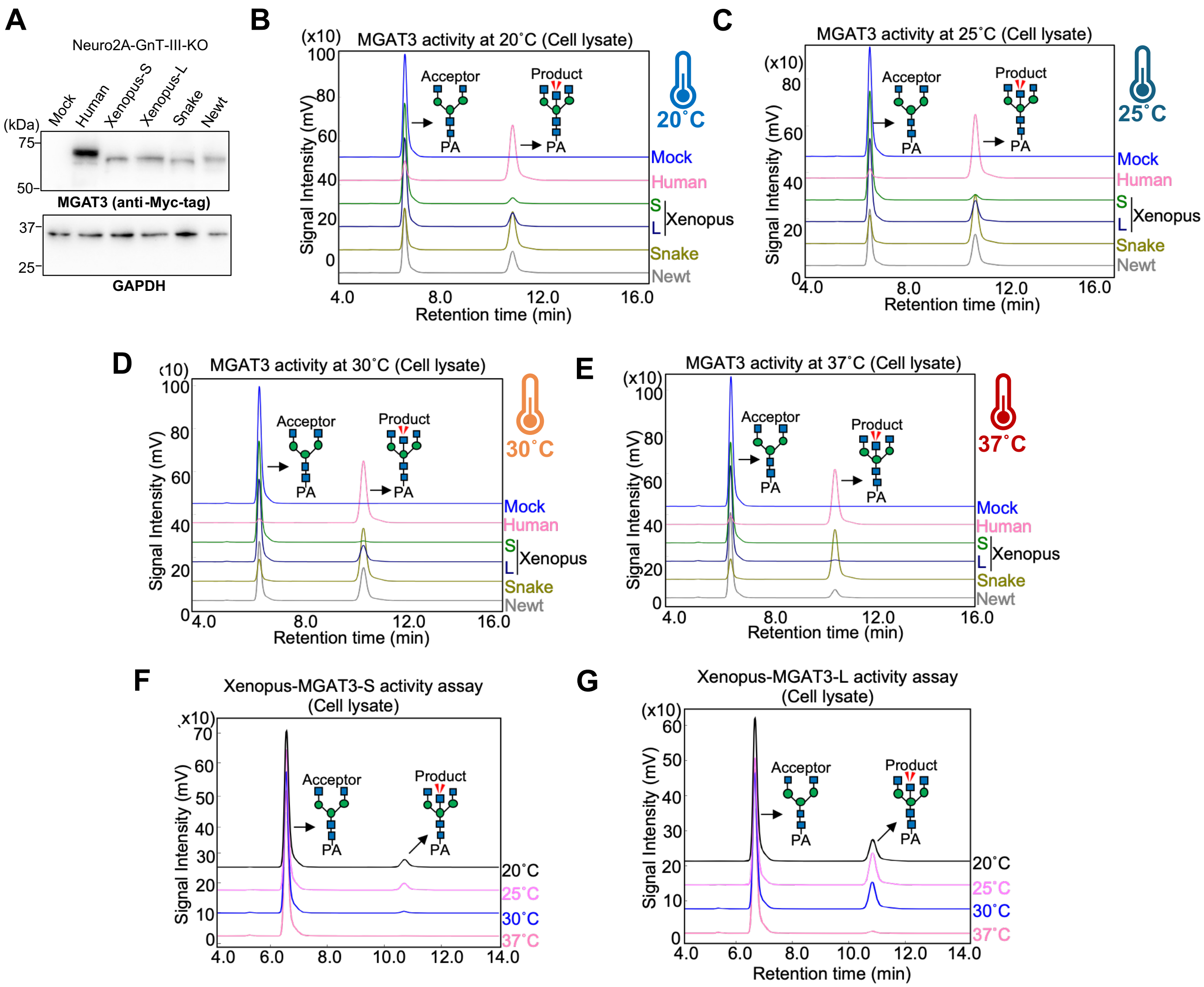

Figure S9. The HPLC-based activity assay of MGAT3 in various species at different reaction temperatures. ***A,*** The Myc-tagged human-, snake-, newt-MGAT3 and *Xenopus*-MGAT3-S and -L were expressed, or the empty vector (pcDNA6-mycHisA) was transfected (Mock) in Neuro2A-MGAT3-KO cells. Cell lysates were prepared after 48 h of transfection, and the cellular proteins were blotted with anti-Myc-tag (upper) and anti-GAPDH (lower) antibodies. ***B-E***, The cell lysates of Neuro2A-MGAT3-KO expressing human, snake, newt-MGAT3, and *Xenopus*-MGAT3-S and -L, or transfected with the empty vector (Mock, pcDNA6-mycHisA) were reacted with the fluorescently labeled oligosaccharide acceptor substrate, GnGnbi-PA at 20˚C (B), 25˚C (C), 30˚C (D) or 37˚C (E). The unreacted substrates and the products with bisecting GlcNAc were quantified by reverse phase HPLC. ***F-G***, HPLC chromatograms of the same activity assay as (B-E) for *Xenopus*-MGAT3-S and -L at different temperatures are shown.

Figure S10

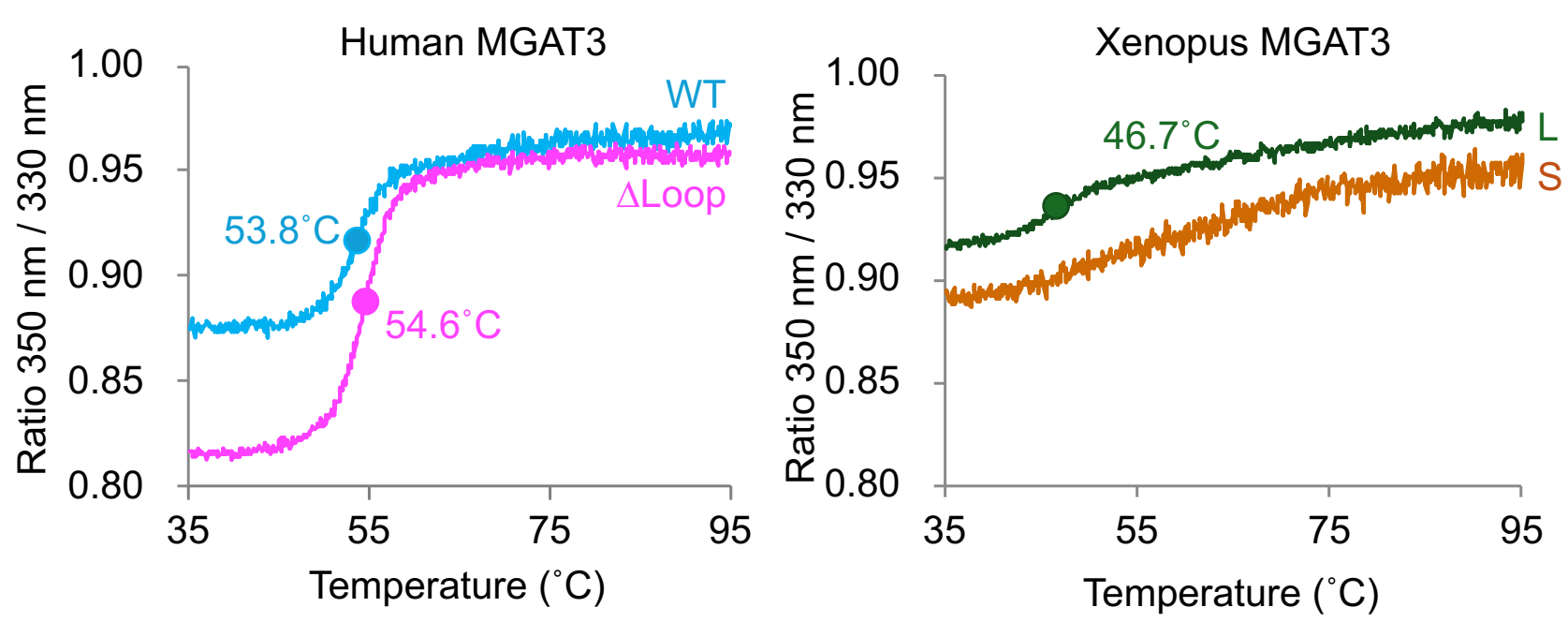

Figure S10. Thermostability analysis of human and *Xenopus* MGAT3.

Figure S11

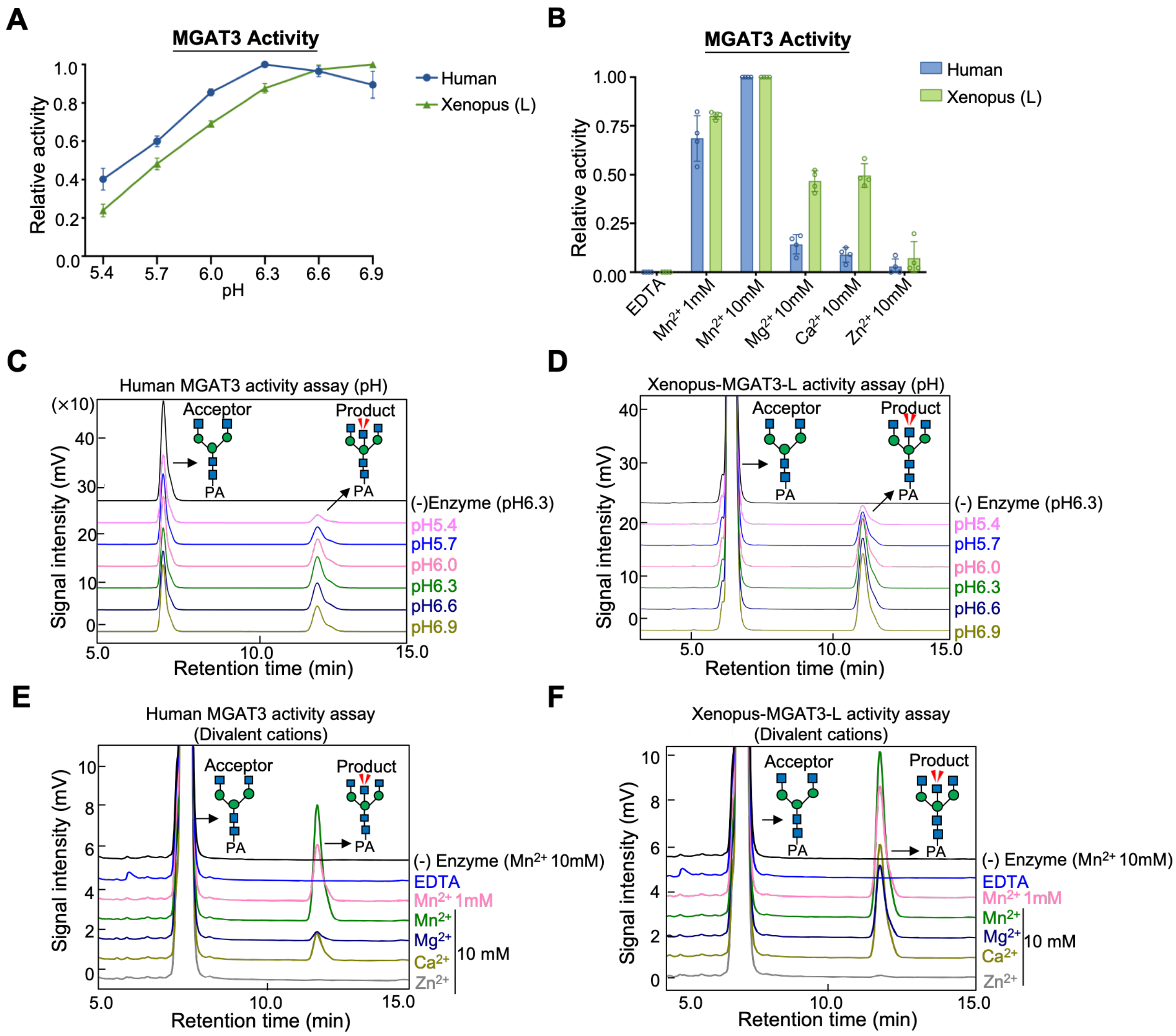

Figure S11 The activity of human and *Xenopus* MGAT3 at different pH values and with different cations. ***A,C,D*** The purified human and *Xenopus* MGAT3 were reacted with the fluorescently labeled oligosaccharide acceptor substrate, GnGnbi-PA, in the reaction buffers with different pH values. The unreacted substrates and the products with bisecting GlcNAc were quantified by reversed phase HPLC. Activity of MGAT3 was calculated according to the peak areas in (C) and (D). The activity relative to that at pH 6.3 for human MGAT3 or that at pH 6.9 for *Xenopus* MGAT3 is shown (n = 3, mean ± SD). ***B,E,F*** The purified human and *Xenopus* MGAT3 were reacted with GnGnbi-PA in the reaction buffers containing different divalent cations. Activity of MGAT3 was calculated according to the peak areas in (E) and (F). The activity relative to that in the buffer containing 10 mM Mn^{2+} is shown (n = 3, mean ± SD).

Figure S12

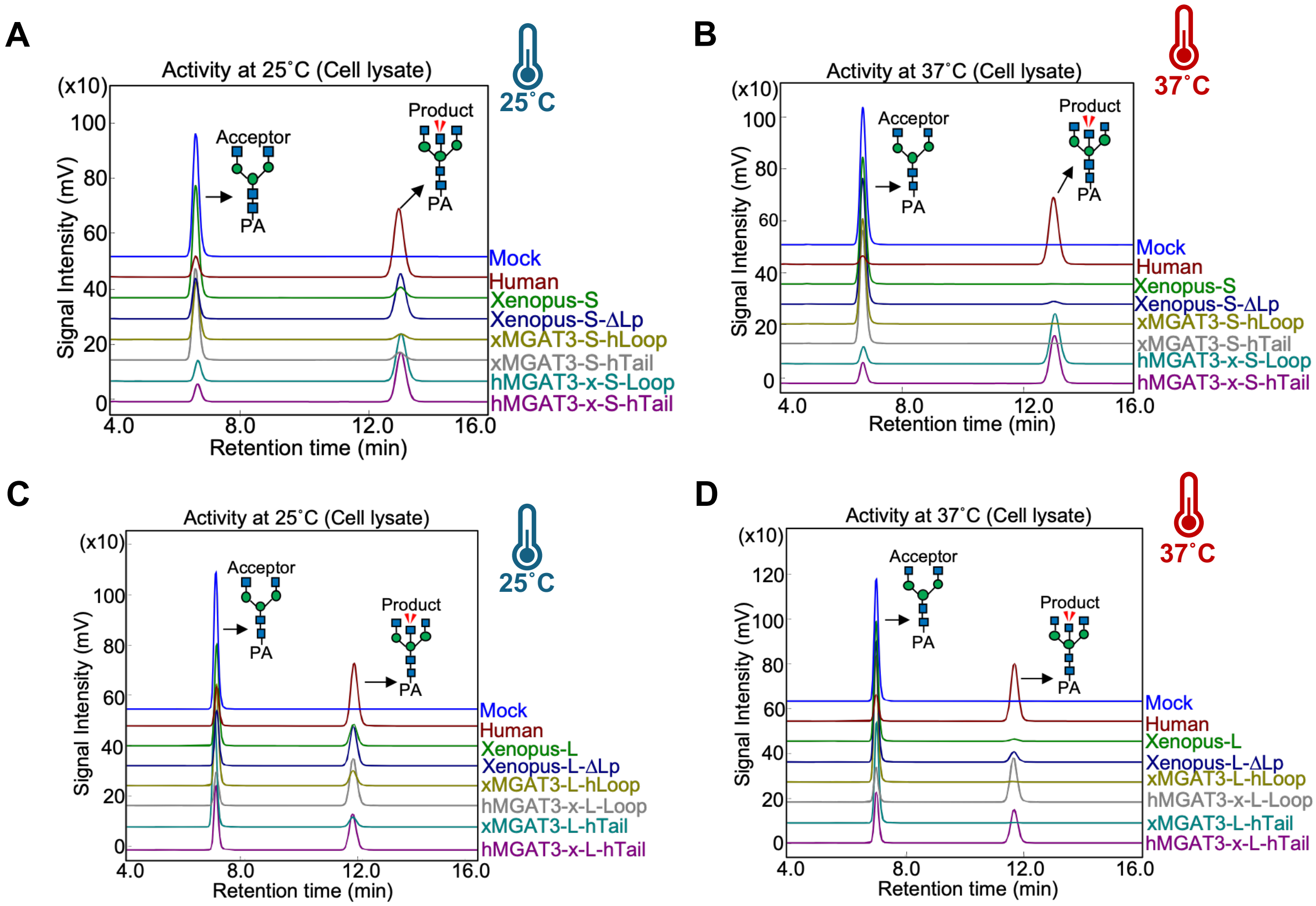

Figure S12. The HPLC-based activity assay of chimeric MGAT3 between *Xenopus*-S or –L and human at different reaction temperatures. *A-D*, The cell lysates of Neuro2A-MGAT3-KO expressing chimeric MGAT3 between *Xenopus*-S and human were reacted with the fluorescently labeled oligosaccharide acceptor substrate, GnGnbi-PA at 25˚C (A) and 37˚C (B). The cell lysates of Neuro2A-MGAT3-KO expressing chimeric MGAT3 between *Xenopus*-L and human were reacted with the fluorescently labeled oligosaccharide acceptor substrate, GnGnbi-PA at 25˚C (C) and 37˚C (D). The unreacted substrates and the products with bisecting GlcNAc were quantified by reverse phase HPLC.

Figure S13

```
10         20         30         40         50         60         70         80         90         100        110        120        130        140        150
                                                                                                   |          |          |          |          |          |          |          |          |          |          |          |          |          |          |

Nemvec_GT17_MGAT3_05571_A   --------------------------------------------------------------MMACRFVIACCRFLPLRRLCSPRKILLSGCFVFLLVTLNLL----------------STSDVNYKATEI-----------------DG--
Exadia_GT17_MGAT3A_B        -----------------------------------------------------------------MRYLRVLC-FLCIR-LCSPRRSIIFAISICLFFTMYLL----------------XNTGMDLPERQI-----------------SG--
Exadia_GT17_MGAT3A          ----------------------------------------------------------------------------MHR---SPRQ--------FFLSERE----------------------S----------------------------
Nemvec_GT17_MGAT3A_B        -------------------------------------------------------------------MVNLRCKTRLLERFIITGSTVVVIVTVVLLVKAGST----------------WNPSLQAVQGNI-----------------HA--
Salsal_GT17_MGAT3A_rBa      --------------------------------------------------------MARLFLQGR---MKMQKHKFIF--FCALALCVFSQLHNYKALHHMPI----------------LS-ELYASSVN-------------------ALS
Oncner_GT17_MGAT3A          --------------------------------------------------------MARLFLHGR---MKMQRHKFFF--FCALALCVFSQLHNYKPLHHMPI----------------LS-ELYASSVN-------------------ALS
Salsal_GT17_MGAT3A_rBb      --------------------------------------------------------MARLYRQGR---TKMRRHKVFL--FGALALCVLYLLQYCKALHHMLM----------------LT-ELYTSSVNL-----------------EALG
Oncner_GT17_MGAT3A_B        --------------------------------------------------------MARLYRQGR---TKMRRHKVFL--FGTLALCVLSLLQYCKALHHMLM----------------LR-ELYTSSVNL-----------------EALG
Esoluc_GT17_MGAT3A_rB       -------------------------------------------------MCVNNGRLSSLWTKLRNQKTKMRRLKVFV--LCLMAFCILLLRHHYQVLYNRLG----------------VR-DLQAASAKF-----------------KALR
Danrer_GT17_MGAT3A_rB       --------------------------------------------------------MGRSYLSVR---MRVKRHKVFL--VCLLGICVVSFLHYYTALHNISL----------------LQ-EISSPSSHL-----------------KTLK
Eleele_GT17_MGAT3A_rB       --------------------------------------------------------------------MRVRRRRLFL--LCCASACVVSLLSCYGVFH------------------------LPRQGLHL-----------------KTTT
Cluhar_GT17_MGAT3A_rB       --------------------------------------------------------------------MKIRRFKALL--FFSVGLCVVSFLHYYKALNQIPM----------------LK-ELSNSPANL-----------------KHLA
Orylat_GT17_MGAT3A          ---------------------------------------MSWLKCISKS----LP----MRCWRLLCLMKMRRHKVFL--LCTVGLCVISFLHYYKALHYVSL----------------LR-ELSAPYPNI-----------------KSFI
Orymel_GT17_MGAT3A          ---------------------------------------MSLLKCTSIC----LP----TRCWRLLCLMKMRRHKVFL--LCTVGLCVISFLHYYKALHYVSL----------------LR-ELSAPYPNI-----------------KSFI
Latcal_GT17_MGAT3A          ---------------------------------------MSWLRGTWAP---LSPQKTPTRCLRLLCLMKMRRHRVFL--LCTVGLCVISFLHYYKALHYVSL----------------LR-ELSAPYPNI-----------------KSFI
Diclab_GT17_MGAT3A          ---------------------------------------MSWLRGTWES---LNLQKTAARCLRLLWLMKMRRHRVFL--LCTVGLCVISFLHYYKALHYVSL----------------LR-ELSAPYPNI-----------------KSFI
Epimoa_GT17_MGAT3A          --------------------------------MGVITAFMSWTRRTWES---QNQQQTPSRCLRFLCLMKMRRHRVFL--LCTVGLCVISFLHYYKALHYVSL----------------LR-ELSAPYPNI-----------------KSFI
Ampoce_GT17_MGAT3A          --------------------------------MHPTSVSLNTVLSTLSLPMVLNPVCNTTMTLR-RCRMKMRRHRVFL--LCTVGLCVISFLHYYKALHYVSL----------------LR-ELSAPYPNI-----------------KSFI
Lepocu_GT17_MGAT3A          --------------------------------------------------------------------MKMRRHRVFL--LCTVGLCVISFLHYYKALHYVSL----------------LR-ELSAPYPNI-----------------KSFI
Sclfor_GT17_MGAT3A          --------------------------------------------------------------------MRMRRYRVFL--LCTVGLCVISFLHYYKALHYVSL----------------LR-ELSAPYPNI-----------------NSFI
Angang_GT17_MGAT3A          -----------------------------------------------------------MWRVIPCGRMKMRRHKVFL--FCTVGLCVISFLHYYKALHYVSL----------------LR-ELSAPYPKI-----------------KSFI
Salsal_GT17_MGAT3A_rAa      ------------------------------------------MERTMDG-----DLSSHHGHFIYCDRMKMRRHRVFL--LCTVGLCVISFLHYYKALHYVSL----------------LK-ELSAPYPNI-----------------KSFI
Salsal_GT17_MGAT3A_rAb      --------------------------------------------------------------------MKMRRHRVFL--LCTVGLCVISFLHYYKALHYVSL----------------LK-ELSAPYPNI-----------------KSFI
Esoluc_GT17_MGAT3A_rA       --------------------------------------------------------------------MKMRRHRVFL--LCTVGLCVISFLHYYKALHYVSL----------------LR-ELSAPYPNI-----------------KSFI
Danrer_GT17_MGAT3A_rA       --------------------------------------------------------------------MKMRRHRVFL--ICTVGLCVISFLHYYKALHYVSL----------------LR-ELSAPYPNI-----------------KSFI
Cluhar_GT17_MGAT3A_rA       --------------------------------------------------------------------MKMRRHRVFL--LCTVGLCVISFLHYYKALHYVSL----------------LR-ELSAPYPNI-----------------KSFI
Eleele_GT17_MGAT3A_rA       --------------------------------------------------------------------MKMRRHRVFL--LCTVGLCVISFLHYYKALHYVSL----------------LR-ELSAPYPNI-----------------KSFI
Amioce_GT17_MGAT3A          ---------------------------------------MTRVTGTATS-----ES------AREIDTMRMRRHRVFL--LCTVGLCVISFLHYYKALHYVSL----------------LR-ELSAPYPNI-----------------RSFI
Acirut_GT17_MGAT3A          --------------------------------------------------------------------MKMRRHRVFL--LCTVGLCVISFLHYYKALHYVSL----------------LR-ELSAPYPNI-----------------KSFI
Acirut_GT17_MGAT3A_B        --------------------------------------------------------------------MKMRRHRVFL--LCTVGLCVISFLHYYKALHYVSL----------------LR-ELSAPYPNI-----------------KSFI
Polspa_GT17_MGAT3A          -----------------------------------------------------------MACLSLLRRMKMRRHRVFL--LCTVGLCLISFLHYYKALHYVTL----------------LR-ELSSPYPNI-----------------KSFF
Galgal_GT17_MGAT3A          ----------------------------------------------MVLYSFREGNSTRLSIAGSTRRMKMRRHKLFLT-LCMAGLCLISFLHFLKALSYVTF----------------PR-DLASLSPNL-----------------VS--
Melgal_GT17_MGAT3A          ----------------------------------------------MVLYSFREGNSTRPSIAGSPQRMKMRRHKLFLT-LCMAGLCLISFLHFLKALSYVTF----------------PR-DLASLSPNL-----------------VS--
Cotcotjap_GT17_MGAT3A       --------------------------------------------------------------------MKMRRHKLFLT-LCMAGLCLISFLHFLKALSYVTF----------------PR-DLASLSPNL-----------------VS--
Aquchrchr_GT17_MGAT3A       --------------------------------------------------------------------MKMRRHKLFLT-LCMAGLCLISFLHFLKALSYVTF----------------PR-ELASLSPNL-----------------VS--
Strcamaus_GT17_MGAT3A       --------------------------------------------------------------------MKMRRHKLFLT-LCMAGLCLISFLHFLKALSYVTF----------------PR-ELASLSPNL-----------------VS--
Pogvit_GT17_MGAT3A          --------------------------------------------------------------------MKMRRHKLFLT-LCMAGLCLISFLHFLKALSYVTF----------------PR-ELASLSPNL-----------------VS--
Anocar_GT17_MGAT3A          ------------------------------------------------------------------MWMKMRRHKLFLT-LCMAGLCLISFLHFLKALSYVTF----------------PR-ELASLSPNL-----------------VS--
Gekjap_GT17_MGAT3A          --------------------------------------------------------------------MKMRRHKLFLT-LCMAGLCLISFLHFLKALSYVTF----------------PR-ELASLSPNL-----------------VS--
Pytbiv_GT17_MGAT3A          --------------------------------------------------------------------MKMRRHKLFLT-LCMAGLCLISFLHFLKALSYVTF----------------PR-ELASLSPNL-----------------VS--
Thasirsir_GT17_MGAT3A       --------------------------------------------------------------------MKMRRHKLFLT-LCMAGLCLISFLHFLKALSYVTF----------------PR-ELASLSPNL-----------------VS--
Musmus_GT17_MGAT3A          --------------------------------------------------------------------MKMRRYKLFLM-FCMAGLCLISFLHFFKTLSYVTF----------------PR-ELASLSPNL-----------------VS--
Muspah_GT17_MGAT3A          ------MEPETSASLAGSRDLRVALLSS---------------------------------------WMKMRRYKLFLM-FCMAGLCLISFLHFFKTLSYVTF----------------PR-ELASLSPNL-----------------VS--
Ratnor_GT17_MGAT3A          --------------------------------------------------------------------MKMRRYKLFLM-FCMAGLCLISFLHFFKTLSYVTF----------------PR-ELASLSPNL-----------------IS--
Mesaur_GT17_MGAT3A          -MGRGEAAADASISPPGAAEPPPPLLLPPLLRPSGRWNVELSIFSAPCLPVCHQSPRRDDKGQKPLKLMKMRRYKLFLM-FCMAGLCLISFLHFFKTLSYVTF----------------PR-ELASLSPNL-----------------VS--
Nangal_GT17_MGAT3A          --------------------------------------------------------------------MKMRRYKLFLM-FCMAGLCLISFLHFFKTLSYVTF----------------PR-ELASLSPNL-----------------VS--
Hetgla_GT17_MGAT3A          ------------------MNPR-------------------------------------------FRRMKMRRYKLFLV-FCMAGLCLISFLHFFKTLSYVTF----------------PR-ELASLSPNL-----------------VS--
Canlup_GT17_MGAT3A          MGREAAADASISPPGAPGAAEPPPPLLPPPLRPSGRWVIFMKNRKSRT----EISHIKPATHLVPGDRMKMRRYKLFLM-FCMAGLCLISFLHFFKTLSYVTF----------------PR-ELASLSPNL-----------------VS--
Vullag_GT17_MGAT3A          --------------------------------------------------------------------MKMRRYKLFLM-FCMAGLCLISFLHFFKTLSYVTF----------------PR-ELASLSPNL-----------------VS--
Lyncan_GT17_MGAT3A          --------------------------------------------------------------------MKMRRYKLFLM-FCMAGLCLISFLHFFKTLSYVTF----------------PR-ELASLSPNL-----------------VS--
Felcat_GT17_MGAT3A          --------------------------------------------------------------------MKMRRYKLFLM-FCMAGLCLISFLHFFKTLSYVTF----------------PR-ELASLSPNL-----------------VS--
Pantigalt_GT17_MGAT3A       --------------------------------------------------------------------MKMRRYKLFLM-FCMAGLCLISFLHFFKTLSYVTF----------------PR-ELASLSPNL-----------------VS--
Hipampkib_GT17_MGAT3A       MGREVATDASISPPGAPGAAEPPPPLLPPLLRPGGRWQKVPGVMATN-----SKAQVPAFSAAWPGSLMKMRRYKLFLM-FCMAGLCLISFLHFFKTLSYVTF----------------PR-ELASLSPNL-----------------VS--
Susscr_GT17_MGAT3A          --------------------------------------------------------------------MKMRRYKLFLM-FCMAGLCLISFLHFFKTLSYVTF----------------PR-ELASLSPNL-----------------VS--
Bostau_GT17_MGAT3A          --------------------------------------------------------------------MKMKRYKLFLM-FCMAGLCLISFLHFFKTLSYVTF----------------PR-ELASLSPNL-----------------VS--
Vicpac_GT17_MGAT3A          --------------------------------------------------------------------MKMRRYKLFLM-FCMAGLCLISFLHFFKTLSYVTF----------------PR-ELASLSPNL-----------------VS--
Panpan_GT17_MGAT3A          MGREAAADASISPPGTPGAAEPPPPLLPPLLRPSGRW---MESCSCRPGSGVQWRDHGSLQLPPPGFKMKMRRYKLFLM-FCMAGLCLISFLHFFKTLSYVTF----------------PR-ELASLSPNL-----------------VS--
Pantro_GT17_MGAT3A          --------------------------------------------------------------------MKMRRYKLFLM-FCMAGLCLISFLHFFKTLSYVTF----------------PR-ELASLSPNL-----------------VS--
Homsap_GT17_MGAT3A          --------------------------------------------------------------------MKMRRYKLFLM-FCMAGLCLISFLHFFKTLSYVTF----------------PR-ELASLSPNL-----------------VS--
Gorgor_GT17_MGAT3A          ------------------------------------------MEADFGMNPTRLTPLRPPKKLGNLERMKMRRYKLFLM-FCMAGLCLISFLHFFKTLSYVTF----------------PR-ELASLSPNL-----------------VS--
```

```
Ponpyg_GT17_MGAT3A           ----------------------------------------------------------------------MRRYKLFLM-FCMAGLCLISFLHFFKTLSYVTF----------------PR-ELASLSPNL---------------VS--
Nomleu_GT17_MGAT3A           --------------------------------------------------------------------MKMRRYKLFLM-FCMAGLCLISFLHFFKTLSYVTF----------------PR-ELASLSPNL---------------VS--
Hylmol_GT17_MGAT3A           --------------------------------------------------------------------MKMRRYKLFLM-FCMAGLCLISFLHFFKTLSYVTF----------------PR-ELASLSPNL---------------VS--
Papanu_GT17_MGAT3A           --------------------------------------------MDRPRVSVAEPIPPPPSFKASSPRMKMRRYKLFLM-FCMAGLCLISFLHFFKTLSYVTF----------------PR-ELASLSPNL---------------VS--
Macmul_GT17_MGAT3A           --------------------------------------------------------------------MKMRRYKLFLM-FCMAGLCLISFLHFFKTLSYVTF----------------PR-ELASLSPNL---------------VS--
Tacacu_GT17_MGAT3A           --------------------------------------------------------------------MKMRRHKLFLT-LCMAGLCLISFLHFLKALSYVTF----------------PR-ELASLSPNL---------------VS--
Ornana_GT17_MGAT3A           --------------------------------------------------------------------MKMRRHKLFLT-LCMAGLCLISFLHFLKALSYVTF----------------PR-ELASLSPNL---------------VS--
Geoser_GT17_MGAT3A           ----------------------------------MRERLKCARPHVGALAGVLHENENVSKAVKKARRMKMRRHKLFLV-LCMAGLCLISFMHFLKALSYVTF----------------PR-ELTALNPNL---------------MS--
Micuni_GT17_MGAT3A           --------------------------------------------------------------------MKMRRHKLFLV-LCMAGLCLISFMHFLKALSYVTF----------------PR-ELAAMSPNL---------------MS--
Rhibiv_GT17_MGAT3A           ------------------------------------MYLEMHTPYDG-----------------NDLRMKMRRHKLFLV-LCMAGLCLISFLHFLKALSYVTF----------------PR-ELAALSPNL---------------MA--
Plewal_GT17_MGAT3A           --------------------------------------------------------------------MKMRRHKWFLV-LCMAGLSLISFLHFLKALSYVRL----------------PR-DLTVFSPNL---------------G---
Lishel_GT17_MGAT3A           --------------------------------------------------------------------MKMRRHKWFLV-LCMAGLSLISFLHFLKALSYVRL----------------PR-DLTVFSPNL---------------G---
Ambmex_GT17_MGAT3A           --------------------------------------------------------------------MKMRRHKWFLV-LCMVGLSLISFLHFLKALSYVRL----------------PR-DLTVFSPNL---------------G---
Xenlae_GT17_MGAT3A_rA        --------------------------------------------------------------------MKMRWHKMFLL-LCMAGLCVISVLHFLKALSYVSL----------------SR-ELVSLSPNF---------------IS--
Xenlae_GT17_MGAT3A_rB        --------------------------------------------------------------------MKMRWHKMFLL-LCMAGLCVISVLHFLKALSYVSL----------------SR-ELVSLSPNF---------------IS--
Xentro_GT17_MGAT3A           --------------------------------------------------------------------MKMRWHKMFLL-LCMAGLCVISVLHFLKALSYVSL----------------SR-ELVSLSPNF---------------IS--
Hylsar_GT17_MGAT3A           ----------------------------------------------------------MHCASAALIWMKMRWHKMFLL-LCMAGLCVISVLHFLKALSYVSL----------------SK-DLVSLSPNF---------------IS--
Bufbuf_GT17_MGAT3A           ---------------------------------------------------------------MLRFRMKMRWHKMFLL-LCMAGLCVISVLHFLKALSYVSL----------------SK-DLVSLSPNF---------------IS--
Rantem_GT17_MGAT3A           --------------------------------------------------------------------MKMRWHKMFLL-LCMAGLCVISVLHFLKALSYVSL----------------SR-ELVSLSPNF---------------IS--
Bombom_GT17_MGAT3A           --------------------------------------------------------------------MKMRWHKMFLL-LCMAGLCVISVLHFLKALSYVSL----------------PR-ELVSLSPNL---------------IS--
Latcha_GT17_MGAT3A           --------------------------------------------------------------------MKMRRHKWFLV-LCMAGLCLISFLHFLKALSYVTI----------------PR-DLSGFNPNI---------------NS--
Latcha_GT17_MGAT3A_B         ------------------------------------------------------------------------------------------------------------------------------------------------------
Rhityp_GT17_MGAT3A           --------------------------------------------------------------------MKMRRRRVVLV-LCMAGLCLISFMHFLKALSYITF----------------SH-ELSAFSPSL---------------RS--
Stetig_GT17_MGAT3A           --------------------------------------------------------------------MKMRRRRVVLV-LCMAGLCLISFMHFLKALSYITF----------------SH-ELSAFSPSL---------------RS--
Leueri_GT17_MGAT3A           --------------------------------------------------------------------MKMRRHRVVLM-LCMAGLCLISFLHFLKALSYITF----------------SR-ELSAFSPSL---------------RS--
Pripec_GT17_MGAT3A           --------------------------------------------------------------------MKMRRHRVVLM-LCMAGLCLISFLHFLKALSYITF----------------SR-EISAFSPSL---------------RS--
Calmil_GT17_MGAT3A           --------------------------------------------------------------------MKMRRHRIVLV-LLMAGLCLISFMHFLKALSYITF----------------SR-ELSSFTPSL---------------RS--
Lamflu_GT17_MGAT3A           -----------------------------------------------------------MAPRTRCSALPLRR-RLLLP-ACVLGLSLAVALQLWQALAPRVAAE-------------IPRLGLKPLSGHL---------------ER--
Letrei_GT17_MGAT3A           -----------------------------------------------------------MAPRTRCSALPSRR-RLLLP-ACVLGLSLAVALQLWQALAPRVAAE-------------IPRLGLKPLSGHL---------------ER--
Petmar_GT17_MGAT3A           -----------------------------------------------------------MAPRIRCSALPLRR-RLLLP-ACVLGLSLAVALQLWQALAPRVAVE-------------IPRLGLKPLSGHL---------------ER--
Myxglu_GT17_MGAT3A           ----------------------------------------------------------------------MRR-RSLML-LCASGLGLLSLLQLHQALRALVS-------------------GLP----HR---------------RE--
Limpol_GT17_MGAT3A           ------------------------------------------------------------------------------------------------------------------------------------------------------
Dapmag_GT17_MGAT3A_A         ----------------------------------------------------------------------------------------------------------------------MVLTVLVFFHSS----------------TS--
Dappul_GT17_MGAT3A_B         ----------------------------------------------------------------------------MISRTLKSFWFHLKQHKRGSRRWIVSIAI------------LQVVTVLVFFHSS----------------PSN-
Hyaazt_GT17_MGAT3A           ---------------------------------------------------------MSPLAVIGRFRRGCPRRELVIFVVVQVLLGLLFFHLYGEHYLPEVIEK------------LQRNSPLVAFDASR---------VDQLDHDSNE
Homame_GT17_MGAT3A           -----------------------------------------------MCLRISLMRSWRRIAAECRLRKRLLLKSVVVLIVVQSVLVYMLVASNHRLQEEKEMEKEDHQGDDTVLGAWVVGQNIVLFTATYGRGTFLSQELQDPRQESGQ
Dappul_GT17_MGAT3A           ------------------------------------------------------------MSLKKLLKKPKTSEDCAVSSFEDKMTPMRRFALWIVTIQIVMIIG------------IMS---VYTFQPG--------------------
Anasim_GT17_MGAT3A           ----------------------------------------------------------------MYYKLDVKLFLLWMLLLVQVLLVVFYLLMAPSSYEYSTN-----------------APELSSDMSNN-------------------
Dromel_GT17_MGAT3_05573      -------------------------------------------------------MVRSTFLATYNQTPVAGVGRLAITKKLVLWLVLVLQVGFIGCIWLLQMSR-------------GQESQQRSAMDQLG-------------GRVN-

Prim.cons.                   MGREAAADASISPPGAPGAAEPPPPLLPPLLRPSGRWVLMSWLR2T35L2SVL2PQMTRLRCL2RLRRMKMRRHKLFLMRLCMAGLCLISFLHFLKALSYVTFAEEDHQGDDTVLGALIPR3ELASLSPNLGRGTFLSQE222223VSFI

                                      160       170       180       190       200       210       220       230       240       250       260       270       280       290       300
                                        |         |         |         |         |         |         |         |         |         |         |         |         |         |         |
Nemvec_GT17_MGAT3_05571_A    -----------------------------LRN----------V--LGWGFW---------------------------------------------------------------------------------------------------
Exadia_GT17_MGAT3A_B         ----------------------------------------------AMQLW---------------------------------------------------------------------------------------------------
Exadia_GT17_MGAT3A           -----------------------------------------------MS-----------------------------------------------------------------------------------------------------
Nemvec_GT17_MGAT3A_B         -----------------------------KPR----------R--LSNEVD---------------------------------------------------------------------------------------------------
Salsal_GT17_MGAT3A_rBa       FLWRGKGNPSNNAHPER-PLPGPVPSDTGLWE--------PEAYREDQGNAEVG-----------------------------------------RKIGRG-------------------------------------------------
Oncner_GT17_MGAT3A           FLWRGKGNPSNNAHPERRPLPGPVPSDTGLWE--------PEAYREDQGN----------------------------------------------------------------------------------------------------
Salsal_GT17_MGAT3A_rBb       FLWRG------NVRSEMKPLPGSRPSDTGLWE--------PGDDREDQGNAKVG-----------------------------------------RRIGHG-------------------------------------------------
Oncner_GT17_MGAT3A_B         FLWRG------NVRSGMKPLPGSRPSNTGLWE--------PGDD---QGNAEVE-----------------------------------------RWIGHG-------------------------------------------------
Esoluc_GT17_MGAT3A_rB        YFWT--------LT-----------TSTNLSE--------PRGYQHNQNG---------------------------------------------RWTGHG-------------------------------------------------
Danrer_GT17_MGAT3A_rB        MLRG------------------------FLWN-------------FPTDDIP--------------------------------------------------------------------------------------------------
Eleele_GT17_MGAT3A_rB        ILGG------------------------FLWGD-------PLVATHPQSNLLS------------------------------------------RIYNHN-------------------------------------------------
Cluhar_GT17_MGAT3A_rB        ALSR------------------------FLWKEKF-----TGGHDISKNAAPGV-----------------------------------------QSQAFL-------------------------------------------------
Orylat_GT17_MGAT3A           MVSG------------------------FFWREKG----AGATPLIPASAD-------------------------------------------DDPRLPVRHNSDTKHRG-G-AGSLGS---RRDEE---LDISDAMRPEGPGVGDHLG
Orymel_GT17_MGAT3A           MVSG------------------------FFWREKG----ASATPLVPASAD-------------------------------------------DDPRLPVRRNPDAKHRG-GGAGSLGS---QRDEE---LDINEG--PERPGVGDHLG
Latcal_GT17_MGAT3A           MVTG------------------------FFWREKG----VAATPLSPASPE-------------------------------------------EAPPLPVIRQMDTKAR--GMAGAVGG---GGGGGGMGLGIGGVM-VGGAGIVGSAG
Diclab_GT17_MGAT3A           MVTG------------------------FFWREKG----VATTPLSPASPE-------------------------------------------EAPPLPVLRQSDSKAR--AVAGAMGGE--GGGGGGMNLGIGGVA-VGGLGIIGSAG
Epimoa_GT17_MGAT3A           MVTG------------------------FFWREKG----VGTTPLSPASPE-------------------------------------------EAPPLPVLRQPDTKAR--AVAGAVGGGGGGGGGGGMGLGIGGVM-VGGAGIVGSAG
Ampoce_GT17_MGAT3A           MVTG------------------------FFWREKG----VATTPLSPASPE-------------------------------------------EAPPLPVLRQSDTKAR--GMAGSVGG---GGGGG---LGTGGVM-VVGPGVIGHAG
Lepocu_GT17_MGAT3A           MVSG------------------------FFWRDG-------ATPLASAPPE-------------------------------------------EALPRRN-KRDRAD------LVLIGS----FSQF------------EMYWHIVSAW
Sclfor_GT17_MGAT3A           MVTG------------------------FFWRE------------QSFPLE-------------------------------------------NSLSGET-K----------------------G------------------------
Angang_GT17_MGAT3A           MVAG------------------------MFWKER-------TTPLSSALLE-------------------------------------------GGPALAM-RLSDNE------AQDSME----LDR-----------------------
```

```
Salsal_GT17_MGAT3A_rAa      MVTG------------------------FFWREG------GSTPLTSAASSSDFAASQE-----------------------------------DGPPPAL-RPSDTKPRIVGAIGPGGG----GGG----------------GRGGGVE
Salsal_GT17_MGAT3A_rAb      MVTG------------------------FFWREG------GGTPLTSAASSSDFAAWQE-----------------------------------DGPPPAL-RPSDTKPRIVGAIGPGGG----GGG----------------GRGGGVE
Esoluc_GT17_MGAT3A_rA       MVTG------------------------FFWREA--------TPLTSAASS-DFAASQK-----------------------------------EGPPPAL-GHSDTKPRVVGPIRPERG----GGRR------------RRRGSGDEAE
Danrer_GT17_MGAT3A_rA       MVTG------------------------FFWKEG-------ST-MSSATPE-------------------------------------------EAPP-------DGK---------LQA----AGG---------------L----ELD
Cluhar_GT17_MGAT3A_rA       MVTG------------------------FFWRER-------ATPMASAVPE-------------------------------------------EGPPPAL-RPSDTKPRGGEDMEDVRM----PGG---------------LGDPGGLV
Eleele_GT17_MGAT3A_rA       IVTG------------------------FFWRER-------AAPLTSAAPE-------------------------------------------EAPPPAL-RPSYVR---------PRA----GDG---------------FR--AVPE
Amioce_GT17_MGAT3A          MVSG------------------------FFWKDGGGAGGESGASFPSAHPEG-------------------------------------------APPPPAPRRASAPPG----EVLPQP----WDR-----------------DVG--E
Acirut_GT17_MGAT3A          MASG------------------------FFWKDG-------DSPLASVEPE-------------------------------------------QAPELLRTPLYDIPLAH-----HL-----------------------------HAQ
Acirut_GT17_MGAT3A_B        MASG------------------------FFWKDG-------DSPLASVEPE-------------------------------------------QAPELLRTPLYDIPLAH-----HL-----------------------------HAQ
Polspa_GT17_MGAT3A          MVSE------------------------FFWKQG-------GTPLGRVEPE-------------------------------------------EAPQLLRTPSYIRELP-------------------------------------LVQ
Galgal_GT17_MGAT3A          ---S------------------------FFWN---------NAPVTPQVSP--------------------------------------------EPGG---------------------------------------------------
Melgal_GT17_MGAT3A          ---S------------------------FFWN---------NAPVTPQVSP--------------------------------------------EPGG---------------------------------------------------
Cotcotjap_GT17_MGAT3A       ---S------------------------FFWN---------NAPVTPQVSP--------------------------------------------EPGG---------------------------------------------------
Aquchrchr_GT17_MGAT3A       ---S------------------------FFWN---------NAPVTPQVSP--------------------------------------------EPGG---------------------------------------------------
Strcamaus_GT17_MGAT3A       ---S------------------------FFWN---------NAPVTPQVSP--------------------------------------------EPGG---------------------------------------------------
Pogvit_GT17_MGAT3A          ---N------------------------LFWN---------NAPITPQVSP--------------------------------------------EPGG---------------------------------------------------
Anocar_GT17_MGAT3A          ---N------------------------FFWN---------NAPVTPQVSP--------------------------------------------EPGG---------------------------------------------------
Gekjap_GT17_MGAT3A          ---N------------------------FFWN---------NAPVTPQVSP--------------------------------------------EPGG---------------------------------------------------
Pytbiv_GT17_MGAT3A          ---N------------------------LFWN---------NAPVTPQASP--------------------------------------------ESGG---------------------------------------------------
Thasirsir_GT17_MGAT3A       ---N------------------------LFWN---------NVPVTPQASP--------------------------------------------DSGG---------------------------------------------------
Musmus_GT17_MGAT3A          ---S------------------------FFWN---------NAPVTPQASP--------------------------------------------EPGG---------------------------------------------------
Muspah_GT17_MGAT3A          ---S------------------------FFWN---------NAPVTPQASP--------------------------------------------EPGG---------------------------------------------------
Ratnor_GT17_MGAT3A          ---S------------------------FFWN---------NAPVTPQASP--------------------------------------------EPGD---------------------------------------------------
Mesaur_GT17_MGAT3A          ---S------------------------FFWN---------NAPVTPQASP--------------------------------------------QPGG---------------------------------------------------
Nangal_GT17_MGAT3A          ---S------------------------FFWN---------NAPVTPQASP--------------------------------------------EPGS---------------------------------------------------
Hetgla_GT17_MGAT3A          ---S------------------------FFWN---------NAPVTPQASP--------------------------------------------EPGG---------------------------------------------------
Canlup_GT17_MGAT3A          ---S------------------------FFWN---------NAPVTPQASP--------------------------------------------EPGS---------------------------------------------------
Vullag_GT17_MGAT3A          ---S------------------------FFWN---------NAPVTPQASP--------------------------------------------EPGS---------------------------------------------------
Lyncan_GT17_MGAT3A          ---S------------------------FFWN---------NAPVTPQASP--------------------------------------------EPGS---------------------------------------------------
Felcat_GT17_MGAT3A          ---S------------------------FFWN---------NAPVTPQASP--------------------------------------------EPGS---------------------------------------------------
Pantigalt_GT17_MGAT3A       ---S------------------------FFWN---------NAPVTPQASP--------------------------------------------EPGS---------------------------------------------------
Hipampkib_GT17_MGAT3A       ---S------------------------FFWN---------NAPVTPQASP--------------------------------------------EPGS---------------------------------------------------
Susscr_GT17_MGAT3A          ---S------------------------FFWN---------NAPVTPQASP--------------------------------------------EPGS---------------------------------------------------
Bostau_GT17_MGAT3A          ---S------------------------FFWN---------NAPVTPQASP--------------------------------------------EPGS---------------------------------------------------
Vicpac_GT17_MGAT3A          ---S------------------------FFWN---------NAPVTPQSSP--------------------------------------------EPGS---------------------------------------------------
Panpan_GT17_MGAT3A          ---S------------------------FFWN---------NAPVTPQASP--------------------------------------------EPGG---------------------------------------------------
Pantro_GT17_MGAT3A          ---S------------------------FFWN---------NAPVTPQASP--------------------------------------------EPGG---------------------------------------------------
Homsap_GT17_MGAT3A          ---S------------------------FFWN---------NAPVTPQASP--------------------------------------------EPGG---------------------------------------------------
Gorgor_GT17_MGAT3A          ---S------------------------FFWN---------NAPVTPQASP--------------------------------------------EPGG---------------------------------------------------
Ponpyg_GT17_MGAT3A          ---S------------------------FFWN---------NAPVTPQASP--------------------------------------------EPGG---------------------------------------------------
Nomleu_GT17_MGAT3A          ---S------------------------FFWN---------NAPVTPQASP--------------------------------------------EPGG---------------------------------------------------
Hylmol_GT17_MGAT3A          ---S------------------------FFWN---------NAPVTPQASP--------------------------------------------EPGG---------------------------------------------------
Papanu_GT17_MGAT3A          ---S------------------------FFWN---------NAPVTPQASP--------------------------------------------EPGD---------------------------------------------------
Macmul_GT17_MGAT3A          ---S------------------------FFWN---------NAPVTPQASP--------------------------------------------EPGD---------------------------------------------------
Tacacu_GT17_MGAT3A          ---S------------------------FFWN---------HAPVTPRASP--------------------------------------------EPAGAP-------------------------------------------------
Ornana_GT17_MGAT3A          ---S------------------------FFWN---------HAPVTPRASA--------------------------------------------GPAGGP-------------------------------------------------
Geoser_GT17_MGAT3A          ---S------------------------FFWD---------TAPVTPQLSA--------------------------------------------VPEP-A-------------------------------------------------
Micuni_GT17_MGAT3A          ---S------------------------FFWD---------TAPVTPQLSA--------------------------------------------VPEP-A-------------------------------------------------
Rhibiv_GT17_MGAT3A          ---S------------------------FFWD---------TAPVTPQLSA--------------------------------------------IPEP-A-------------------------------------------------
Plewal_GT17_MGAT3A          ----------------------------FLWL---------TAPVTPQAEP--------------------------------------------DPPPVG-------------------------------------------------
Lishel_GT17_MGAT3A          ----------------------------FLWM---------TAPVTPQADP--------------------------------------------DPPPVG-------------------------------------------------
Ambmex_GT17_MGAT3A          ----------------------------FLWM---------TAPVTPQFEP--------------------------------------------DPPPAG-------------------------------------------------
Xenlae_GT17_MGAT3A_rA       ---G------------------------FLWE---------PQPLIPRLMD--------------------------------------------RAEVG--------------------------------------------------
Xenlae_GT17_MGAT3A_rB       ---G------------------------FLWD---------SRPLIPQLMD--------------------------------------------RAEVG--------------------------------------------------
Xentro_GT17_MGAT3A          ---G------------------------FLWE---------SRPLIPQFVD--------------------------------------------RAEVG--------------------------------------------------
Hylsar_GT17_MGAT3A          ---G------------------------FFWE---------SRPLIFQHLE--------------------------------------------RTDDG--------------------------------------------------
Bufbuf_GT17_MGAT3A          ---G------------------------FFWE---------SRPLIPQHIE--------------------------------------------RTDDG--------------------------------------------------
Rantem_GT17_MGAT3A          ---G------------------------FFWE---------SRPLLLQHVE--------------------------------------------RMDDS--------------------------------------------------
Bombom_GT17_MGAT3A          ---G------------------------FFWE---------SRPLNPHIVA--------------------------------------------RSDVA--------------------------------------------------
Latcha_GT17_MGAT3A          ---I------------------------FIISRYFW-----DAPTLPLVTA--------------------------------------------KPDL-E-------------------------------------------------
Latcha_GT17_MGAT3A_B        ------------------------------------------------------------------------------------------------------------------------------------------------------
Rhityp_GT17_MGAT3A          ---I------------------------FVNS---------------QFFW--------------------------------------------KPNA---------------------------------------------------
Stetig_GT17_MGAT3A          ---I------------------------FVNS---------------QFFW--------------------------------------------KPNT---------------------------------------------------
Leueri_GT17_MGAT3A          ---I------------------------FVDS---------------HLFW--------------------------------------------KPNE---------------------------------------------------
Pripec_GT17_MGAT3A          ---I------------------------FVDS---------------HIFW--------------------------------------------KQNE---------------------------------------------------
```

```
Calmil_GT17_MGAT3A          ---I------------------------FVDS---------------QFLW--------------------------------------------KESV---------------------------------------------------
Lamflu_GT17_MGAT3A          ----------------------------LFWSG--------HGGDDDDGPR--------------------------------------------QNDA---------------------------------------------------
Letrei_GT17_MGAT3A          ----------------------------LFWSG--------HGGDDDDGPR--------------------------------------------QNDT---------------------------------------------------
Petmar_GT17_MGAT3A          ----------------------------LFWSG--------H---DDEGLR--------------------------------------------QSDA---------------------------------------------------
Myxglu_GT17_MGAT3A          ----------------------------VFLSG--------VTPPDHGGP----------------------------------------------------------------------------------------------------
Limpol_GT17_MGAT3A          ------------------------------------------------------------------------------------------------------------------------------------------------------
Dapmag_GT17_MGAT3A_A        -------------------------------------------VSRNSLSR--------------------------------------------QKLS---------------------------------------------------
Dappul_GT17_MGAT3A_B        -------------------------------------------VWRNPFAV--------------------------------------------QIQP---------------------------------------------------
Hyaazt_GT17_MGAT3A          KEYDHKS----STYKTASEGDTGSHKLDLMSTL-----TLVDVTSKNNLPA--------------------------------------------RHMADEDK-----------------------------------------------
Homame_GT17_MGAT3A          ESVYVEA----QKDNTTEASPGVRVGVVSINKTGH---RGGDSKSETRLPAGRRGGVNGSGEDNPHNQNVPGALTPARKDHGDGENQAGETVGKERAMKDKVEGERSIGEVEEEKEDPIGGRGVEEEF------------VKYRGEDKMA
Dappul_GT17_MGAT3A          --------------------------------------------WSSTLTA---------------------------------------------------------------------------------------------------
Anasim_GT17_MGAT3A          -------------------------------------------VFPVKFVP---------------------------------------------------------------------------------------------------
Dromel_GT17_MGAT3_05573     ----------------------------FVASG--------EAPMEKMALVG--------------------------------------------------------------------------------------------------

Prim.cons.                  MVTSGKGNPSNN222E2KPLPG2RPSDTFFWNEGG2AG3VPNAPVTPQASPG2FAASQESGEDNPHNQNVPGALTPARKDHGDGENQAGETVGKEEPGGAG2R2SDTKPRGVG3AG4LGGG2GGGGGGGMGLGIGGVMRV2G2G2GGHAG

                                    310       320       330       340       350       360       370       380       390       400       410       420       430       440       450
                                     |         |         |         |         |         |         |         |         |         |         |         |         |         |         |

Nemvec_GT17_MGAT3_05571_A   ---------KKTAGKEGKK-------------------------TNHFVSAEA------------------------------------FFLHRPRN------------------------------------IPLIKVDPITDY---VY
Exadia_GT17_MGAT3A_B        ---------KSKVSKQKDY-------------------------LKSFG--KG------------------------------------FSPQRPKV------------------------------------IKVGPPALLSEF---VS
Exadia_GT17_MGAT3A          -------------------------------------------------------------------------------------------------------------------------------------------DHISRS---MS
Nemvec_GT17_MGAT3A_B        ---------NNKPSKRGE---------------------------------EV------------------------------------FHHLRPRS------------------------------------ITLPDPVPMSHH---VT
Salsal_GT17_MGAT3A_rBa      ----SEDPWRKENPDSGNVNRM--------------------TVTLP-NPKGP------------------------------------WGELHKRS------------------------------------FHLSD-DPTPFF---VR
Oncner_GT17_MGAT3A          ----AEEPWRKEQPDSGNVNRM--------------------TVTLP-DPKGP------------------------------------LGELHKRS------------------------------------FHLSD-DPTQFF---VR
Salsal_GT17_MGAT3A_rBb      ----GEDQWGKEQPDSGNVNRM--------------------TVTLP-DPKGP------------------------------------WGELHKRS------------------------------------IHLHD-DPTSFF---VR
Oncner_GT17_MGAT3A_B        ----GEDPWGKEQPDSGNVNRM--------------------TVTLP-DPKGP------------------------------------WGELHKRS------------------------------------FRLRD-DPTPFF---AR
Esoluc_GT17_MGAT3A_rB       ----NEDQRGDEPAAPTDQDTV--------------------TFTLP-DPTGP------------------------------------SGELHKRS------------------------------------FLLRD-DATQFF---VR
Danrer_GT17_MGAT3A_rB       -----FKQDRQKSSHQKDFVD---------------------LPDLP-DAKQV------------------------------------VTNLHKQI------------------------------------FELRD-DSTPFF---TH
Eleele_GT17_MGAT3A_rB       ----VAEKSLKQNSYRKDLAEE--------------------VLVLP-DPTGP------------------------------------NGELHKRA------------------------------------YVLHD-DLNSYF---AR
Cluhar_GT17_MGAT3A_rB       ----VWRPSGVKSQRPVEDTGM--------------------FPQIPPEPSRP------------------------------------MGDLHLRT------------------------------------FQLKE-DTTSYF---AR
Orylat_GT17_MGAT3A          FRV-----PDPPHPWEKPDENNLGELSKDGEAEVYH--------AKHFEREHP-----KAHLG-RAHIAV------FLDNQQWG-PLKSMGDLHTRT------------------------------------HRLQD-DKTSYF---VS
Orymel_GT17_MGAT3A          LRV-----PEPPHPWEKPEENDLGELSKDRGAEDYH--------AKHFEREHP-----KAHLG-REHIAV------FLDNHQWD-PLRSMGDLHTRT------------------------------------HRLQD-DKTSYF---VS
Latcal_GT17_MGAT3A          LEMRLREDPVAPHPWEKPEENQRGDSPNEERAEDHLKPRNPSHQTQPAGPDLP-----EVPLDGKG----------IRKDKLSD-PLDSMGDLHTRT------------------------------------HQLQD-DKTPYF---VR
Diclab_GT17_MGAT3A          LEMKLREEPAPPHPWEKPEENQRGDSANEERAEDHLKPRNPSHQAQPAGPDLP-----EVPSLRKEHRVV------LRKDKFPD-PLESMGDIHIRT------------------------------------HQLQD-DKTPYF---VR
Epimoa_GT17_MGAT3A          LEMRLREEPAPPHPWEKPEENERGDTPNEERAEDHLKQRSASHPAQDAGPDLP-----EVPFVDKEHLDA------LLNNQLPD-PIESMGDLHTRK------------------------------------FQLQD-DKTPYF---VR
Ampoce_GT17_MGAT3A          LEMRQREEPAPPHPWEKPEENQLGEAPNEKRAEDHLNLRNP---AQPARPDPP-----EVPLAGKEHAVV------FAHEQLPD-PVESLGDLHTRT------------------------------------HQLQD-DKTPYF---VR
Lepocu_GT17_MGAT3A          VSW--VNYVQAWESWTFKALPK---ISSPPQEN-----W-----IQKQNRNLA-----PDLQKNKELP----------RMTLPD-PRGPLGDLHRRS------------------------------------FELRD-DPNHYF---VH
Sclfor_GT17_MGAT3A          ---------QGAKEWQVMAK-------------------------QQNHRNAS-----QAKLDLKNLP----------AFTLPD-PSGLHGDLHRRS------------------------------------FQLHD-DQTPYF---VR
Angang_GT17_MGAT3A          ------EMDKDLIAWYPSPR------------D-----G-----VRPNYQNRK-----DAAVR-KSPQ----------MFALPD-PMGTLRDLHRHS------------------------------------FQLHD-DTTPYF---VR
Salsal_GT17_MGAT3A_rAa      MRL--REEPAAPQPWGQPEEEPRHRDTDPNEEREGEDLH-----FKPWYPSHPAHPVRPYAPEEKASAGIDDLVVPS-KAPSPD-PLETLGDIHTRT------------------------------------HQLQD-DPTSYF---VR
Salsal_GT17_MGAT3A_rAb      MRL--REEPAAPQPWGQPEEEPRHRDTDPNEEREGEDLH-----FKPWYPSHPAHLVRPYAPEEKASAGIDDQVVPS-KASSPD-PLETLGDIHTRT------------------------------------HQLQD-DPTSYF---VR
Esoluc_GT17_MGAT3A_rA       RGT--CRATAVGSARGTQTQTHVQRSSGPVKMQQSEDLL-----FKSWNPGHPEHPPRPDTPAEKAPA------MPP-KAPSPS-PLEALGDIHTRT------------------------------------HPLQD-DPTPYF---VR
Danrer_GT17_MGAT3A_rA       LDT--GQEPEAPQPWRRPEEVQ-RRSSSTTEVH---KVG-----LVPWKPSSPTDP-KADPQRESEVRSAINKLQPR-HVSTPD-PLETLGDLHRRS------------------------------------HLLQD-DRSPYF---VR
Cluhar_GT17_MGAT3A_rA       MEA--EEDPQVPRPWGRPEEPP-HGDPDGTQEQNE-RLE-----LTPWHPNHP----AAR-LRQEAPHVVVEELDPT-TAPTPD-PIESMGDLHHRT------------------------------------HHLQN-DHTSYF---VR
Eleele_GT17_MGAT3A_rA       LDK--AGEPRAPQPWARPDEPH-HKDLELIQLEKKGNGK-----MMVWNPSSPPHQ-RPDLLRPNAPSMVSQEFRPQPPASTHD-PITFLGDLHKRT------------------------------------FTLKE-DYGPYF---VR
Amioce_GT17_MGAT3A          GREE--EEDRDGGKRSKPGEIGELDATEELWTQDDHAP------SQPHPNSAPQTRPSADATAPSHPQS---------PRPRPD-PMGPLGDLHRRT------------------------------------FQLQE-DETPYF---VR
Acirut_GT17_MGAT3A          REE---NEPALGFKETKPVDPIPVERGDARLPHPKQE-------VLNLDPNAQP---PVVDVSERQRQRA--------AAPHPG-APEAISDLHRRA------------------------------------FVLRD-QPNTYF---IR
Acirut_GT17_MGAT3A_B        REE---NEPALGFKETKPVDPIPVERGDARLPRPKQE-------VLNLDPNAQP---PVVDVSERQRQRA--------AAPHPG-APEAISDLHRRA------------------------------------FVLRD-QPNTYF---IR
Polspa_GT17_MGAT3A          REE---NEPAQGLKGPRRADRTGAERGGARLPRPEQ--------VLNLDPNTQP---PAEDASERQRGAP--------QPDPPGGAPETISDLHRRP------------------------------------FVLQD-KPNTYF---TR
Galgal_GT17_MGAT3A          -----AEFLRTPLYSHSP---L----------------------LQPLPPSRA------------------------------------SEELHKVE------------------------------------FVLPE-DTTEYF---VR
Melgal_GT17_MGAT3A          -----AEFLRTPLYSHSP---L----------------------LQPLPPSRA------------------------------------SEELHKVE------------------------------------FVLPE-DTTEYF---VR
Cotcotjap_GT17_MGAT3A       -----AEFLRTPLYSHSP---L----------------------LQPLPPSRA------------------------------------SEELHKVE------------------------------------FVLPE-DTTEYF---VR
Aquchrchr_GT17_MGAT3A       -----AEFLRTPLYSHSP---L----------------------LQPLPPSRA------------------------------------SEELHKVE------------------------------------FVLPE-DTTEYF---VR
Strcamaus_GT17_MGAT3A       -----AEFLRTPLYSHSP---L----------------------LQPLPPSRA------------------------------------SEELHRVE------------------------------------FVLPE-DTTEYF---VR
Pogvit_GT17_MGAT3A          -----PDFLRTPLYSHSP---L----------------------LQPLSPSRA------------------------------------GEELHKVE------------------------------------FVLPE-DTTEYF---VH
Anocar_GT17_MGAT3A          -----PEFLRTPLYSHSP---L----------------------LQPLSPSRA------------------------------------SEELHKVE------------------------------------FVLPE-DIAEYF---VH
Gekjap_GT17_MGAT3A          -----PEFLRTPLYSHSP---L----------------------LQPLSPSRA------------------------------------SEELHRVE------------------------------------FVLPE-DISEYF---VH
Pytbiv_GT17_MGAT3A          -----PEILRTPLYSHSP---L----------------------LQPLSPSRA------------------------------------SEELHKAE------------------------------------FVLPE-DTAEYF---VH
Thasirsir_GT17_MGAT3A       -----PEILRTPLYSHSP---L----------------------LQPLSPSKA------------------------------------GEELHKAE------------------------------------FVLPE-DTTEYF---VR
Musmus_GT17_MGAT3A          -----PDLLRTPLYSHSP---L----------------------LQPLSPSKA------------------------------------TEELHRVD------------------------------------FVLPE-DTTEYF---VR
Muspah_GT17_MGAT3A          -----PDLLRTPLYSHSP---L----------------------LQPLSPSKT------------------------------------TEELHRVD------------------------------------FVLPE-DTTEYF---VR
Ratnor_GT17_MGAT3A          -----PDLLRTPLYSHSP---L----------------------LQPLSPSKA------------------------------------TEELHRVD------------------------------------FVLPE-DTTEYF---VR
Mesaur_GT17_MGAT3A          -----PDLLRTPLYSHSP---L----------------------LQPLSPSKA------------------------------------TEELHRMD------------------------------------FVLSE-DTTEYF---VR
Nangal_GT17_MGAT3A          -----PDLLRTPLYSHSP---L----------------------LQPLSPSKA------------------------------------TEELHRMD------------------------------------FVLPE-DTTEYF---VR
Hetgla_GT17_MGAT3A          -----PGLLRTPLYSHSP---L----------------------LQPLSPSRA------------------------------------TEELHRVD------------------------------------FVLPE-DTTEYF---VR
```

```
Canlup_GT17_MGAT3A          -----PDLLRTPLYSHSP---L----------------------LQPLSPSKA------------------------------------TEELHRVD------------------------------------FVLPE-DTTEYF---VR
Vullag_GT17_MGAT3A          -----PDLLRTPLYSHSP---L----------------------LQPLSPSKA------------------------------------TEELHRVD------------------------------------FVLPE-DTTEYF---VR
Lyncan_GT17_MGAT3A          -----PDLLRTPLYSHSP---L----------------------LQPLSPSKA------------------------------------TEELHRVD------------------------------------FVLPE-DTTEYF---VR
Felcat_GT17_MGAT3A          -----PDLLRTPLYSHSP---L----------------------LQPLSPSKA------------------------------------TEELHRVD------------------------------------FVLPE-DTTEYF---VR
Pantigalt_GT17_MGAT3A       -----PDLLRTPLYSHSP---L----------------------LQPLSPSKA------------------------------------TEELHRVD------------------------------------FVLPE-DTTEYF---VR
Hipampkib_GT17_MGAT3A       -----PDLLRTPLYSHSP---L----------------------LQPLSPSKA------------------------------------TEELHRMD------------------------------------FVLPE-DTTEYF---VR
Susscr_GT17_MGAT3A          -----PDLLRTPLYSHSP---L----------------------LQPLSPSKA------------------------------------TEELHRMD------------------------------------FAMPE-DTAEYF---VR
Bostau_GT17_MGAT3A          -----PDLLRTPLYSHSP---L----------------------LQPLSPSKA------------------------------------TEELHRMD------------------------------------FVLPE-DTTEYF---VR
Vicpac_GT17_MGAT3A          -----PDLLRTPLYSHSP---L----------------------LQPLSPSKA------------------------------------AEELHRMN------------------------------------FVLPE-DTTEYF---VR
Panpan_GT17_MGAT3A          -----PDLLRTPLYSHSP---L----------------------LQPLPPSKA------------------------------------AEELHRVD------------------------------------LVLPE-DTTEYF---VR
Pantro_GT17_MGAT3A          -----PDLLRTPLYSHSP---L----------------------LQPLPPSKA------------------------------------AEELHRVD------------------------------------LVLPE-DTTEYF---VR
Homsap_GT17_MGAT3A          -----PDLLRTPLYSHSP---L----------------------LQPLPPSKA------------------------------------AEELHRVD------------------------------------LVLPE-DTTEYF---VR
Gorgor_GT17_MGAT3A          -----PDLLRTPLYSHSP---L----------------------LQPLPPSKA------------------------------------AEELHRVD------------------------------------LVLPE-DTTEYF---VR
Ponpyg_GT17_MGAT3A          -----PDLLRTPLYSHSP---L----------------------LQPLPPSKA------------------------------------AEELHRVD------------------------------------LVLPE-DNTEYF---VR
Nomleu_GT17_MGAT3A          -----PDLLRTPLDSHSP---L----------------------LQPLPPSKA------------------------------------ADELHRVD------------------------------------LVLPE-DTTEYF---VR
Hylmol_GT17_MGAT3A          -----PDLLRTPLDSHSP---L----------------------LQPLPPSKA------------------------------------ADELHRVD------------------------------------LVLPE-DTTEYF---VR
Papanu_GT17_MGAT3A          -----PDLLRTPLYSHSP---L----------------------LQPLPPSKA------------------------------------AEELHRVD------------------------------------FVLPE-DTTEYF---VR
Macmul_GT17_MGAT3A          -----PDLLRTPLYSHSP---L----------------------LQPLPPSKA------------------------------------AEELHRVD------------------------------------FVLPE-DTTEYF---VR
Tacacu_GT17_MGAT3A          -----AEAPRAPLSSHSP---L----------------------LQPLRPSGA------------------------------------GEELHRLD------------------------------------FVLPE-DTADYF---VR
Ornana_GT17_MGAT3A          -----PEAPRPPLSSHSP---L----------------------LQPLPPSGA------------------------------------GEELHRLD------------------------------------FVLPE-DTADYF---VR
Geoser_GT17_MGAT3A          -----PEVLRTPLYSHSP---L----------------------LQPLPPSK-------------------------------------STELHKAD------------------------------------FFLKE-DSTEYF---IR
Micuni_GT17_MGAT3A          -----PEALRTSLYSHSP---L----------------------LQPLPPSK-------------------------------------STELHKAY------------------------------------LFLKE-DNTEYL---IR
Rhibiv_GT17_MGAT3A          -----PEVLRTPLYSHSP---L----------------------LQPLPPQQX------------------------------------STELHKVD------------------------------------FFLKE-DSAEYF---VQ
Plewal_GT17_MGAT3A          -----PEFLRTPLYPNPP---L----------------------LQPLHP-AA------------------------------------FSDLHKAH------------------------------------LYLPE-DTTEYF---VR
Lishel_GT17_MGAT3A          -----LEFLRTPLYPNPP---L----------------------LQPLNP-DA------------------------------------FSDLHKAH------------------------------------LYLAE-DTTEYF---VR
Ambmex_GT17_MGAT3A          -----AELLRTPLYPNAP---L----------------------LQPLHP-DA------------------------------------PSELHKAH------------------------------------LFLPE-DTTEYF---VR
Xenlae_GT17_MGAT3A_rA       -----TQVLRAPEYYRPPQLNY----------------------NPPRPPSSVQ----------------------G-------EH-LSAEDMHLVD------------------------------------FVLQE-DNTEYF---VH
Xenlae_GT17_MGAT3A_rB       -----TQVLRSPEYYRPPQLNN----------------------NPPRPPSSVR----------------------G-------EH-LSAEDLHLVE------------------------------------FVLQE-DVTEYF---VY
Xentro_GT17_MGAT3A          -----TQVLRTPEYYHPPQLNN----------------------NPPRPPSSIQ----------------------G-------EH-LRAEDMHLVD------------------------------------FVLQE-DTTEYF---VH
Hylsar_GT17_MGAT3A          -----TQVLRTPGYYRPPEAAV----------------------QQPRPPPSGH----------------------G-------PG-ISTDELHLKE------------------------------------FVLQE-DTAEYF---VR
Bufbuf_GT17_MGAT3A          -----TQFLRTPGYYRPPEAAI----------------------HQPRPPPSGH----------------------S-------QGVISTDELHLKE------------------------------------LILQE-DTTEHF---VR
Rantem_GT17_MGAT3A          -----AQVLRTPGYYRPPQLSV----------------------QHPKPPPSSR----------------------G-------ES-LSIDDIHLKE------------------------------------FVLQE-DTAEYF---VR
Bombom_GT17_MGAT3A          -----NQILRTPGYYRPPPLHQ----------------------NQPKPLPSRV----------------------G-------EH--RIDDLHVAE------------------------------------FELQE-DTTEYF---VR
Latcha_GT17_MGAT3A          -----PEVIRGPLYSRSPP--L----------------------QLRRRPALH------------------------------------PTELHKME------------------------------------FILQR-DTMEYF---IR
Latcha_GT17_MGAT3A_B        --------------MQSPK--V----------------------RQNATPPLK------------------------------------MGEGHKTE------------------------------------MNLN-------------
Rhityp_GT17_MGAT3A          -------VE-DVNTSPG---------------------------LQVTVKAQNG----------------------A------------RPPLHRAL------------------------------------FELKD-DETKYF---VQ
Stetig_GT17_MGAT3A          -------AV-NVNTSPG---------------------------LPITVKAQNG----------------------A------------RPPLHRAL------------------------------------FELKD-DETKYF---VQ
Leueri_GT17_MGAT3A          -------GE-DASSGLA---------------------------LQASLGGHQG----------------------G------------WPYLHRAQ------------------------------------FQLKD-DETEYF---IR
Pripec_GT17_MGAT3A          -------EE-DASAGLG---------------------------LPTAPNAHQG----------------------G------------WPHLHRAQ------------------------------------FQLKD-DETEYF---IR
Calmil_GT17_MGAT3A          -------ALREAETEPAP--------------------------LSALPPSPPP----------------------G------------SEEPYRAT------------------------------------FHMRD-ERTEYF---VR
Lamflu_GT17_MGAT3A          ------ADGNS--ASSSP---------------------------ASLIELAR--------------------------------------ELGGRV------------------------------------FTMRD-DRAEYF---VR
Letrei_GT17_MGAT3A          ------ADDNSSSASSSP---------------------------ASLIELAR--------------------------------------ELGGRV------------------------------------FTMRD-DRSEYF---VR
Petmar_GT17_MGAT3A          ------ANGNSSSASSSP---------------------------ASLIELAR--------------------------------------ELGGRV------------------------------------LTMRD-DHEEYF---VR
Myxglu_GT17_MGAT3A          --------------SLAP---------------------------TSLEPVDL--------------------------------------YL--RS------------------------------------FHLSD-DMDEYY---YV
Limpol_GT17_MGAT3A          --------------------------------------------------------------------------------------------MYVEN------------------------------------FKLVT-FSRSGF---VT
Dapmag_GT17_MGAT3A_A        -------VYSGGIETSTN----------------------------PSPSVG-------------------------------------FNENLIVS--------------------------------------LAE-DETLHF---QP
Dappul_GT17_MGAT3A_B        -------VQN-ELQTSVN----------------------------SLPMIS-------------------------------------FDENRVVT--------------------------------------LED-DEFSHF---QP
Hyaazt_GT17_MGAT3A          -------LANKNREPSAA----------------------------PASVSENTTDFHLENRSKM-----------------------IFNNEQTYT--------------------------------------LKD-DNNQYF---VT
Homame_GT17_MGAT3A          LLVEENDIYRANIEKKSDDGVK---------------------KYAPETTVTVRKNMTKKKNNTSGMDDK------------------SYTKNAVVNGEPNGVGTTGDGEDSVGVVQTSDFKFRTYNKSNPSEFVLSF-DTRKYF---QD
Dappul_GT17_MGAT3A          ------FYAAGEAEESYN-------------------------EVVTYPIILP-------------------------------------SLPESYT--------------------------------------LVD-DDRPYF---TF
Anasim_GT17_MGAT3A          ---------KTKTESS-----------------------------QAMQTLG--------------------------------------YLMGEQS------------------------------------IILKS-DDRSYF---NS
Dromel_GT17_MGAT3_05573     ------GTHCPHLQENVN------------------------RYDKDFYQMKP-------------------------------------ERLNES--------------------------------------HLPDYNVPAYVDAEMG

Prim.cons.                  LE2RLP2LLRTPLYSHSPEENLRGE2SDER2AEDHLKLRNPSTVLQPLPPSKAPHP2RPVPL2EK2222VDDE2VPGLKAPLPDEPLESTEELHRVDGEPNGVGTTGDGEDSVGVVQTSDFKFRTYNKSNPSEFVLPEPDTTEYFDAEVR
```

```
                                   460       470       480       490       500       510       520       530       540       550       560       570       580       590       600
                                     |         |         |         |         |         |         |         |         |         |         |         |         |         |         |
Nemvec_GT17_MGAT3_05571_A  TNGSRHCLKQGTKQVK---------------------------------------------------------------------------ERECVCNERWFG-KRCHIPESIHYSNQLQTK---TFLKFR------KSARRIVYAAPFN
Exadia_GT17_MGAT3A_B       MEG--ECLNQGTKEIK---------------------------------------------------------------------------EGKCICKDGWFE-KSCNVPESIYFANQIQX----SLLQLR------YSARRIVYAVXFN
Exadia_GT17_MGAT3A         RNT--ACLNLGTKYML---------------------------------------------------------------------------YEECECRDGWHG-KSCSIPDCLYYSYRRHSM---TTAEKREIYLRKKTARRIIYAVLFN
Nemvec_GT17_MGAT3A_B       SNHHYTCLDHGTRYKT---------------------------------------------------------------------------FEQCLCREGWYG-DSCDIPDSLYGSIDLMKIRRKTRLYLR-----ANTARNVVYAAPFN
Salsal_GT17_MGAT3A_rBa     TKSGEYCFREGTEITSLKDHLGQKV-------QEP-G-------EGQRKILHAHQE---------EPK----SAQEKTA--------WGKRLVKCMCRPGWHG-PHCGIPTMVQHSNLPT----KARLTLR------KVPRRVINAININ
Oncner_GT17_MGAT3A         TKSGAYCFREGTEMTYLKDHLGQKV-------QEP-G-------EGQRKILHAHQE---------EPK----SAQEKTA--------WGKRLVKCMCRPGWHG-PHCGIPTTVQHSNLPT----KARLTLR------KVPRRVINAININ
Salsal_GT17_MGAT3A_rBb     TKSGAYCFREGTEIAFLKDDSGQRV-------QAA-R-------EGQRKILHAQQE---------EPK----SAQGKSA--------RGKRLVKCMCRQGWHG-PHCGIPTMVQHSNLPN----KGSLMPR------MVPRRVINAININ
Oncner_GT17_MGAT3A_B       TKSGAYCFREGTEMAFLKDDSGQTV-------QAA-R-------EGQRKILHAQQE---------EPK----SAQGKYA--------RGKRLVKCMCRQGWHG-PHCGIPTMVQHSNLPT----KGSLMPR------MVPRRVINAININ
Esoluc_GT17_MGAT3A_rB      TKSGATCFREGTDMAFLK--VGERV-------QQT-G-------VGWHKILHFPQE---------EMK----RDQGQSS--------LGKSLANCTCRPGWHG-PHCGIPTMIQHSNLPS----KWRLMPR------KVPRRVINAININ
Danrer_GT17_MGAT3A_rB      TESGALCFHEGTDLAALA--SQGR-------------------------LLKHYEE---------RTP----LKDDLIK--------SGKKDVRCPCRQGWHG-PQCGIPTVVYHSNLPS----KSKVTPR------KIPRRVINAININ
Eleele_GT17_MGAT3A_rB      TETGDLCFREGMEVASPK--VKERE-------TID-N-------TGLRIILHHQEE---------ENQ----VPMNLT-----------IKVEQCVCRPAWHG-PECGVPTVVQHSNIPT----KSHLSPR------KVPRRIINAININ
Cluhar_GT17_MGAT3A_rB      TKSGRMCFRKGTKTSTKKDNLGESQ-------PKE-S-------YASRRALLAVQS---------GNRS-LGPAWNSSV--------LAKRSVRCRCLQGWHG-YHCGIPTVVQHSNLPT----KSRLAPR------EVPRRVINAINVN
Orylat_GT17_MGAT3A         TKAGALCFKQGTEMASQK----DDAIKMVGAESNMAAEG----AAAQRKPLQLKER---------P---SMSPKTKMRAR-S-----GGKRLVKCVCRPGWHG-PYCGVPTMVYHSNLPT----KERLTPR------ETPRRVINAINVN
Orymel_GT17_MGAT3A         TKAGALCFKQGTEMASQK----DYSVKVV--EPNMAADG----AAVQRKPLELKER---------P---SVSPKTKTRAR-S-----GGKRLVKCVCRPGWHG-PYCGVPTMVYHSNLPT----KERLTPR------ETPRRVINAINVN
Latcal_GT17_MGAT3A         TKAGALCFRQGTEVATPK----EYSGKSGGTAANGAGEGARAGAAGQRKPLEVQQQ---------P---SIAPKAKARAR-G-----NGKRLVKCVCRPGWHG-PYCGVPTMVYHSNLPT----KERLTPR------ETPRRVINAININ
Diclab_GT17_MGAT3A         TKAGALCFRQGTEVATPK----EYSGKSGGAVANGA------GAAGQRKPLEVQQQ---------P---SIAPKAKARAR-G-----NGKRLVKCVCRPGWHG-PYCGVPTMVYHSNLPT----KERLTPR------ETPRRVINAINVN
Epimoa_GT17_MGAT3A         TKAGALCFRQGTEVATPK----EYSGKSGGSVANGAGDG--ARAAGQRKPLEVQQQ---------P---SVAPKAKTRVR-G-----NGKRLVKCVCRPGWHG-PYCGVPTMVYHSNLPT----KERLTPR------ETPRRVINAININ
Ampoce_GT17_MGAT3A         TKAGALCFRQGTEVATPK----EYSGKPGGAVANGAGGAGRAGAAGQRKPLELQQQ---------S---SIAPKAKVRAR-G-----NGKRLVKCVCRPGWHG-PYCGVPTMVYHSNLPT----KERLTPR------EAPRRVINAINVN
Lepocu_GT17_MGAT3A         TKAGALCFRQGTEMAVPK----SA-----DDRAKG----------SARKILEQPEL----Q-GGGGGGGGGGGGAKPKR---------GKRLVKCVCRPGWHG-PYCGVPTMVYHSNLPT----KERLAPR------EVPRRVINAINVN
Sclfor_GT17_MGAT3A         TKSGALCFRQGTEMAVPR----EH-----PGKSGATV-------ASQRRILQHQEA----S-S--------TSHFKAKR---------GKRLVKCVCRQGWHG-PYCGVPTMVYHSNLPT----KKRLTPR------EVPRRIINAININ
Angang_GT17_MGAT3A         TKAGALCFRQGTEMATPK----DS-----VVREGARL-------AGHRRILQHQNT----A-H--------SPRARPQR---------GRRLVRCVCRQGWHG-PYCGIPTMVQHSNLPT----KDRLTPR------EVPRRVINAINVN
Salsal_GT17_MGAT3A_rAa     TKAGALCFRQGTEVATPR----DDAARPGGPTVTGTEVGSRAGSPGQRKPLAVLNQSHQQPLHPSSSGTSMPPKAKSRGR-G------GKRLVKCVCRPGWHG-PYCGVPTMVYHSNLPT----KERLTPR------ERPRRVINAINVN
Salsal_GT17_MGAT3A_rAb     TKAGALCFRQGTEVATPR----DDAARPGGPTVTGTEVGSRAGSPGQRKPLAVLNQSHQQPLHPSSSGTSMPPKAKSRGR-G------GKRLVKCVCRPGWHG-PYCGVPTMVYHSNLPT----KERLTPR------ERPRRVINAINVN
Esoluc_GT17_MGAT3A_rA      TKAGALCFRQGTEAAAPR----EDAARPGGATSTGTGVGPRPGSAGQRKPLEAPNR---HPLQPLSSGTAAPPKAKPRGR-G------GKRLVKCVCRPGWHG-PYCGVPTMVYHSNLPT----KERLTPR------EKPRRVINAINVN
Danrer_GT17_MGAT3A_rA      TKAGALCFRQGTEVAPPK----DE---TLKVKPSAAG-------VIQRRILQKPDE---SKTSGKSVEESRLSRGKPKR---------GKRLVKCVCRAGWHG-PYCGVPTMVYHSNLPT----KERLMPR------ETPRRVINAINVN
Cluhar_GT17_MGAT3A_rA      TKAGALCYRKGTEVAPPP----GQ---AKAVNPTAAAN------HGQRQLLQQPIQ---PQQQ-HPEEQAGTLHAKPKR---------GKRLVKCVCRPGWHG-PYCGVPTMVYHSNLPT----KERLTPR------ETPRRIINAINVN
Eleele_GT17_MGAT3A_rA      TKAGALCFRQGTEAAHPR----DDGGGAGKGRPTASA-------SLQRKILQAGEE---PGTGGRPVEGSSPMRSKPWK---------GKRLVKCVCRAGWHG-PYCGVPTLVQHSNLPT----RERLAPR------DRPRRVINAINVN
Amioce_GT17_MGAT3A         TRAGALCFREGTEMAPPR----GG---AGGTGEAGEG-----AVLGVRKILQEEGS----------RAGGGVAGTRPRGR--------GRRLVKCVCRPGWHG-PYCGVPTMVQHSNLPT----KERLAPR------ERPRRVLNAINVN
Acirut_GT17_MGAT3A         TKAGARCFRQGTETAERE----GH-------SPVAKP--------AQRQLLFKEEE--------QEGATTGPRLPKTKR---------GRRLVRCVCRPGWHG-PYCGVPTVVQHSNLPT----KDRLTPR------EKPRRTINAINVN
Acirut_GT17_MGAT3A_B       TKAGARCFRQGTETAERE----GH-------SPVAKP--------VQRQLLFKEEE--------QEGATTGPRLPKTKR---------GRRLVRCVCRPGWHG-PYCGVPTVVQHSNLPT----KDRLTPR------EKPRRTINAINVN
Polspa_GT17_MGAT3A         TKAGAHCFRQGTETAERE----GQ-------TPPAKP--------ARRQLLFKQQE--------EEEEVAGPRLSRMKR---------GRRLVKCVCRPGWHG-PSCGVPTVVQHSNLPT----KERLAPR------DRPRRVINAINVN
Galgal_GT17_MGAT3A         TKAGGVCFKPGTKVL-------EKP-------PAGGR-------PEERVDGVASGR---------PAR----KPLSAG-------GTKRRKWVECVCLPGWHG-PSCGVPTVVQYSNLPT----KDRLVPR------EIPRRVINAININ
Melgal_GT17_MGAT3A         TKAGGVCFKPGTKVL-------EKP-------PVGGR-------PEERADGAASGR---------PAR----KPLSAG-------GTKRRKWVECVCLPGWHG-PSCGVPTVVQYSNLPT----KDRLVPR------EIPRRVINAININ
Cotcotjap_GT17_MGAT3A      TKAGGVCFKPGTKVL-------EKP-------PVGGQ-------LEERADGVASGR---------PAR----KPLSAS-------GTKRRKWVECVCLPGWHG-PSCGVPTVVQYSNLPT----KDRLVPR------EIPRRVINAININ
Aquchrchr_GT17_MGAT3A      TKAGGVCFKPGTKVL-------EKP-------PVGGR-------PEERADGAASGR---------SAR----KPLSAG-------GTKRRKWVECVCLPGWHG-PSCGVPTVVQYSNLPT----KDRLVPR------EIPRRVINAINVN
Strcamaus_GT17_MGAT3A      TKAGGVCFKPGTKVL-------EKP-------PVGGR-------PEERADGAASGR---------PAR----KPLSAS-------GAKRRKWVECVCLPGWHG-PSCGVPTVVQYSNLPT----KDRLVPR------EIPRRVINAINVN
Pogvit_GT17_MGAT3A         TKAGGVCFKPGTKVL-------EKP-------PAG-K-------LDEKTEGAVSGQ---------VGR----KPLSAS-------GTKRRKWVECVCLPGWHG-PSCGVPTVVQYSNLPT----KDRLTPR------EVPRRVINAINVN
Anocar_GT17_MGAT3A         TKAGGVCFKPGTKVL-------EKP-------PPG-R-------PDEKIEGAASGR---------ISR----KPLSAN-------GTKRRKWVECVCLPGWHG-PSCGVPTVVQYSNLPT----KDRLTPR------EVPRRVINAINVN
Gekjap_GT17_MGAT3A         TKAGGVCFKPGTKVL-------EKP-------LVG-R-------LEEKLEGAASER---------AGR----KPLSTN-------GTKRRKWVECVCLPGWHG-PSCGVPTVVQYSNLPT----KDRLTPR------EVPRRVINAINVN
Pytbiv_GT17_MGAT3A         TKAGGVCFKPGTKIL-------EKP-------LAG-R-------PDEKIEGVISGR---------TGR----KPLSAT-------GTKRRKWVECMCLTGWHG-PSCGVPTVVQYSNLPT----KDRLMPR------EVPRRVINAINVN
Thasirsir_GT17_MGAT3A      TKAGGVCFKPGTKIL-------EKP-------LAG-R-------PEEKIEGVISGR---------SGR----KPLSAS-------GTKRRKWVECMCLTGWHG-PSCGVPTVVQYSNLPT----KDRLTPR------EVPRRVINAINVN
Musmus_GT17_MGAT3A         TKAGGVCFKPGTRML-------EKP-------SPG-R-------TEEKPEVSEGSS------ARGPARRPMRHVLSTRERLGS-RG-TRRKWVECVCLPGWHG-PSCGVPTVVQYSNLPT----KERLVPR------EVPRRVINAININ
Muspah_GT17_MGAT3A         TKAGGVCFKPGTRML-------EKP-------LPG-R-------TEEKPEVAEGSS------ARGPARRPMRHVLSTRERLGS-RG-TRRKWVECVCLPGWHG-PSCGVPTVVQYSNLPT----KERLVPR------EVPRRVINAININ
Ratnor_GT17_MGAT3A         TKAGGVCFKPGTRML-------EKP-------SPG-R-------TEEKTKVAEGSS------VRGPARRPMRHVLSARERLGG-RG-TRRKWVECVCLPGWHG-PSCGVPTVVQYSNLPT----KERLVPR------EVPRRVINAININ
Mesaur_GT17_MGAT3A         TKAGGVCFKPGTKML-------EKP-------S-G-R-------REEKPEVAEGSS------ARGPARKPMRHVLSTPERLGS-RG-TRRKWVECVCLPGWHG-PSCGVPTVVQYSNLPT----KERLVPR------EVPRRVINAININ
Nangal_GT17_MGAT3A         TKAGGVCFKQGTKIL-------EKP-------S-W-G-------RPEKPEVAEGSS------ARGPPRRPTRHVLSARERPGG-RS-PRRKWVECVCLPGWHG-PSCGVPTVVQYSNLPT----KERLVPR------EVPRRVINAININ
Hetgla_GT17_MGAT3A         TKAGGVCFKPGTKML-------EKP-------PSG-R-------PEEKPEGADGSSG-----ARGPARRPPRHVLSAREHAGSSRG-ARRKWVECVCLPGWHG-PSCGVPTVVQYSNLPT----KERLVPR------EVPRRVINAININ
Canlup_GT17_MGAT3A         TKAGGVCFKPGTKML-------ERP-------LSG-R-------PEEKAEGADGSP------ARGPAR----HLLSTRERPGA-RG-ARRKWVECVCLPGWHG-PSCGVPTVVQYSNLPT----KERLVPR------EVPRRVINAININ
Vullag_GT17_MGAT3A         TKAGGVCFKPGTKML-------ERP-------LSG-R-------PEEKAEGADGSP------ARGPAR----HLLSTRERPGA-RG-ARRKWVECVCLPGWHG-PSCGVPTVVQYSNLPT----KERLVPR------EVPRRVINAININ
Lyncan_GT17_MGAT3A         TKAGGVCFKPGTKML-------EKP-------PSG-R-------PDEKAEGADGSS------ARGPAR----HLLSTRERPGA-RG-ARRKWVECVCLPGWHG-PSCGVPTVVQYSNLPT----KERLVPR------EVPRRVINAINVN
Felcat_GT17_MGAT3A         TKAGGVCFKPGTKML-------EKP-------PSG-R-------PDEKAEGADGSS------ARGPAR----HLLSTRERPGP-RG-ARRKWVECVCLPGWHG-PSCGVPTVVQYSNLPT----KERLVPR------EVPRRVINAINVN
Pantigalt_GT17_MGAT3A      TKAGGVCFKPGTKML-------EKP-------PSG-R-------PDEKAEGADGSS------ARGPAR----HLLSTRERPGA-RG-ARRKWVECVCLPGWHG-PSCGVPTVVQYSNLPT----KERLVPR------EVPRRVINAINVN
Hipampkib_GT17_MGAT3A      TKAGGVCFKPGTKML-------EKP-------PSG-R-------PEEKAEAGDGTS------ARGPGR----QLLSTRERPGG-RS-ARRKWVECVCLPGWHG-PSCGVPTVVQYSNLPT----KERLVPR------EVPRRVINAININ
Susscr_GT17_MGAT3A         TKAGGVCFKPGTKTL-------EKP-------PSG-R-------PEEKAEVGDGSS------ARGPAR----HLLSTRERPGA-RS-ARRKWVECVCLPGWHG-PSCGVPTVVQYSNLPT----KERLVPR------EVPRRIINAININ
Bostau_GT17_MGAT3A         TKAGGVCFKPGTKML-------EKP-------PSG-R-------PEEKVEAGDGASSS----ARGPGR----QLLSTRERAGS-RGGARRKWVECVCLPGWHG-PSCGVPTVVQYSNLPT----KERLVPR------EVPRRVINAININ
Vicpac_GT17_MGAT3A         TKAGGVCFKPGTKML-------EKP-------PSG-R-------PEDKAEAGEGSS------ARGPAR----HLLSTRERSGG-RG-GRRKWVECVCLPGWHG-PSCGVPTVVQYSNLPT----KERLVPR------EVPRRVINAININ
Panpan_GT17_MGAT3A         TKAGGVCFKPGTKML-------ERP-------PPG-R-------PEEKPEGANGSS------ARRPPR----YLLSARERTGG-RG-ARRKWVECVCLPGWHG-PSCGVPTVVQYSNLPT----KERLVPR------EVPRRVINAINVN
Pantro_GT17_MGAT3A         TKAGGVCFKPGTKML-------ERP-------PPG-R-------PEEKPEGANGSS------ARRPPR----YLLSARERTGG-RG-ARRKWVECVCLPGWHG-PSCGVPTVVQYSNLPT----KERLVPR------EVPRRVINAINVN
Homsap_GT17_MGAT3A         TKAGGVCFKPGTKML-------ERP-------PPG-R-------PEEKPEGANGSS------ARRPPR----YLLSARERTGG-RG-ARRKWVECVCLPGWHG-PSCGVPTVVQYSNLPT----KERLVPR------EVPRRVINAINVN
Gorgor_GT17_MGAT3A         TKAGGVCFKPGTKML-------ERP-------PPG-R-------PEEKPEGANGSS------ARRPPR----YLLSARERTGG-RG-ARRKWVECVCLPGWHG-PSCGVPTVVQYSNLPT----KERLVPR------EVPRRVINAINVN
Ponpyg_GT17_MGAT3A         TKAGGVCFKPGTKML-------ERP-------PPG-R-------PEEKPEGANGSS------ARRPPR----YLLSARERTGG-RG-ARRKWVECVCLPGWHG-PSCGVPTVVQYSNLPT----KERLVPR------EVPRRVINAINVN
Nomleu_GT17_MGAT3A         TKAGGVCFKPGTKML-------ERP-------PPG-R-------PEEKPEGANGSS------ARRPPR----YLLSARERTGG-RG-ARRKWVECVCLPGWHG-PSCGVPTVVQYSNLPT----KERLVPR------EVPRRIINAINVN
```

```
Hylmol_GT17_MGAT3A        TKAGGVCFKPGTKML-------ERP-------PPG-R-------PEEKPEGANGSS------ARRPPR----YLLSARERTGG-RG-ARRKWVECVCLPGWHG-PSCGVPTVVQYSNLPT----KERLVPR------EVPRRIINAINVN
Papanu_GT17_MGAT3A        TKAGGVCFKPGTKML-------ERP-------PPG-R-------PEEKPEGANGSS------ARRPPR----YLLSARERTGG-RG-ARRKWVECVCLPGWHG-PSCGVPTVVQYSNLPT----KERLVPR------EVPRRVINAINVN
Macmul_GT17_MGAT3A        TKAGGVCFKPGTKML-------ERP-------PPG-R-------PEEKPEGANGSS------ARRPPR----YLLSARERTGG-RG-ARRKWVECVCLPGWHG-PSCGVPTVVQYSNLPT----KERLVPR------EVPRRVINAINVN
Tacacu_GT17_MGAT3A        TKAGGVCFRPGTRAP-------ERP-------PAG-R-------ADDRGAGASSGR---------PGR----RPLSGG---GG-GPRRRRKWVECVCLPGWHG-PSCGVPTVVQYSNLPT----KDRLVPR------ARPRRVINAINVN
Ornana_GT17_MGAT3A        TKAGGVCFKPGTRAP-------ERP-------PPG-R-------ADDRGAGAASGR---------PGR----RPLSGG---GG-GPKRRRKWVECVCLPGWHG-PSCGVPTVVQYSNLPT----KDRLVPR------ARPRRVINAINVN
Geoser_GT17_MGAT3A        TKSGGMCFKQGTKTF-------ERV-------TTG-H-------AEEKQEHASGHH---------PGR----KPLNVK-------GPKRRKWVECVCLPGWHG-PSCGVPTMVQHSSLPT----KDRLMPR------EVPRRVINAININ
Micuni_GT17_MGAT3A        TKSGGLCFKQGTKKF-------ERV-------PTG-H-------TEEKQEHASGHH---------PGR----KPLNAV-------GPKRRKWVECVCLPGWHG-PSCGVPTVVQYSNLPT----KDRLVPR------EVPRRVINAININ
Rhibiv_GT17_MGAT3A        TKAGGMCFKQGTKML-------EKV-------PTG-Y-------AEERQEHASGRH---------LGR----KPLHMG-------APKRRKWVECVCLPGWHG-PSCGVPTVVQYSNLPT----KDRLVPR------EVPRRIINAININ
Plewal_GT17_MGAT3A        TKAGGLCFKPGTQTL-------DRL-------PTGVH-------GEDRNERPSGRQ---------PAR----RQLSGS------RT-KRKTHVECPCLPGWHG-PSCGVPTVVQYSNLPT----KDRLTPR------AHPRRVINAINVN
Lishel_GT17_MGAT3A        TKAGGLCFKPGTQTL-------DRL-------PAGVH-------GEDRNERPSGRQ---------PTR----KQLSGT------RS-KRKTHVECPCLPGWHG-PSCGVPTVVQYSNLPT----KDRLTPR------AHPRRVINAINVN
Ambmex_GT17_MGAT3A        TKAGGICFKPGTQTI-------DRL-------TAGGH-------GEDRAERPSGHQ---------SPR----KPLGST------RA-KRKTRVECPCLPGWHG-PSCGVPTVVQYSNLPT----KDRLVPR------ARPRRVINAINVN
Xenlae_GT17_MGAT3A_rA     TKSGGVCYKQGTKSL-------DRT-------PSE-K-------PERSFHRKQAAG---------APR--------PQ---------QRRKWVECVCLPGWYG-PSCGVPTVVQHSNLPT----KDRLTPR------LKPRRVINAININ
Xenlae_GT17_MGAT3A_rB     TKSGGVCYKQGTKSL-------DRT-------P-E-K-------PERSSQRRQAPG---------AARQ---QQ--TQ---------QRRKWVECVCLPGWYG-PSCGVPTVVQHSNLPT----KDRLTPR------LKPRRVINAININ
Xentro_GT17_MGAT3A        TKSGGVCYKQGTKSL-------DRT-------PSE-K-------PERSSHRRQAPG---------APRQ---QQ--TQ---------QRRKWVECVCLPGWYG-PSCGVPTVVQHSNLPT----KDRLTPR------SKPRRVINAININ
Hylsar_GT17_MGAT3A        TKSGGVCFKPGTKSL-------DRL-------PSE-K-------PERSLPRKVSPI---------ISRQ---PQQPTQ---------KRRKWVECVCLPGWHG-PSCGVPTVVQHSNLPT----KDRLTPR------LKPRRVINAININ
Bufbuf_GT17_MGAT3A        TKSGGLCFKSGTKSL-------DRL-------PSE-K-------PERSSQRKVSPS---------ISRQ---SQQQIQ---------KRRKWVECVCLPGWHG-PSCGVPTVVQHSNLPT----KDRLTPR------LKPRRVVNAININ
Rantem_GT17_MGAT3A        TKSGGVCFKPGTKAL-------DRP-------LSE-K-------PERPSPRKASSG---------VSRQ---QQPQTQ---------RRRKWVECVCLPGWHG-PSCGVPTVVQHSNLPT----KDRLTPR------LKPRRVINAININ
Bombom_GT17_MGAT3A        TKSGGVCFKPGTKSL-------DRP-------PSE-K-------SERSPPRKQSPG---------TVRQ---QQQPTQ---------KRRKWVDCVCLPGWHG-PSCGVPTVVQHSNLPT----KDRLTPR------LKPRRVINAININ
Latcha_GT17_MGAT3A        TKAGAECFKQGTQTQ-------TEG-------RLR-------------VLQGKQPT-----------R----KLLSGSQ---------RGRKWVECPCRPGWHG-PSCGVPTVVQHSNLPT----KDRLRPR------ARPRSVINAINMN
Latcha_GT17_MGAT3A_B      ------WIWHGRRLN-------RE---------------------------G-------------------YLPFPS---------RGNTCFSIPCSQTSGGQQSLRHLRWYNTPNLPT----KDRLRPR------ARLHSVINAINVN
Rhityp_GT17_MGAT3A        TKAGAICFKQGTKMA-------AGS-------VEGAV-------DWDHNAVDKQLAQK----M--ANRG-----FSGRP---------RRRWVGCPCLPGWHG-PYCGVPTMVQYSNLPT----KERLSPR------PMPRRVINAVNIN
Stetig_GT17_MGAT3A        TKAGAICFKQGTKMA-------AAS-------VDGAV-------DWDHNAMDKQLAQK-------ANRG-----FSGRP---------RRRWVGCPCLPGWHG-PYCGVPTMVQYSNLPT----KERLSPR------PMPRRVINAVNIN
Leueri_GT17_MGAT3A        TKAGAICFKQGTKTENRM----AGS-------MEGGV-------AWARGSLDKPLTHK----A--VGLG-----VGGRP---------RRRWVGCPCLPGWHG-PYCGVPTMVQHSNLPT----KERLTPR------AVPRRVINAINVN
Pripec_GT17_MGAT3A        TKAGAICFKQGTKTENKM----VGS-------MEGAG-------ALGRNSMDKPLTHK----A--VGLG-----VSGRP---------RRRWVGCPCLPGWHG-PYCGVPTMVQYSNLPT----KERLSPR------VVPHRVINAININ
Calmil_GT17_MGAT3A        TRAGAVCFKHGTKRE-------VRS-------MTGSA-------EEGRGPSNRQPTRR----A--QAR-------QGR----------RRRWVGCVCRPGWHG-PSCGVPTLVQYSNLPT----KDRLSPR------RVPRRLINAININ
Lamflu_GT17_MGAT3A        TPAGALCFREGSLRP-------EEP--------S-----------SLPVLVGSVGS---------VG----LEPNASSGP-------SVPPAAGCACRAGWQG-RHCSVPSVVHHSNLGARH--RAALAPR------AVPRRVVNAVNVN
Letrei_GT17_MGAT3A        TPAGALCFREGSLRP-------EEP--------S-----------SLPVPVGSVGG---------VG----LEPNASSGP-------SVPPAAGCACRAGWHG-RHCSVPSVVHHSNLGARH--RAALAPR------AVPRRVVNAVNVN
Petmar_GT17_MGAT3A        TPAGALCFREGSLHP-------EAP--------S-----------SLPAPVGAVGG---------VG----LEPNASSGP-------SAPPAAGCACRAGWHG-RHCSVPSVVHYSNLGARH--RAALAPR------AAPRRVVNAVNVN
Myxglu_GT17_MGAT3A        TLSGLRCLKEGSRR--------EPL--------S-----------SLQSTLTSRAG---------V-----YELAGAKG-------------EHCFCRPGWHG-PHCSIPTVAQHSNLPS----RGDLRLR------TSPRRVLNVVNVN
Limpol_GT17_MGAT3A        ISPNLTCLISGTNLT------------------------------KTTLEGDGR-----------------------------------SCVCLQGWYG-KDCGLPQSFSASTFPW-I--RNQLKRR------TRPRRVINAININ
Dapmag_GT17_MGAT3A_A      VGIAPFCYVEGTDVEA-----------------------------TLS---------------------------------------TNSSKCVCRTNYFG-HECGIPASAWHRTISKKYH-RWPLKPR------KVPRRIIHGLNIN
Dappul_GT17_MGAT3A_B      VGLAPLCYLEGTSVSK-----------------------------TLS---------------------------------------SNSSKCLCRANYFG-HECGIPAAVWHRTISKKYH-RWPLKPR------KMPRRIIHGLNIN
Hyaazt_GT17_MGAT3A        MSPKLICYGEGTDLEK-----------------------------MKTI--------------------------------------VNASSCLCKQRYYG-ADCGLPDAIWNSPYGQTNN-HDNLKRR------QVPRRVINGLPLL
Homame_GT17_MGAT3A        IGENRTCFIGGTDIER-----------------------------TEQ---------------------------------------SPEGGCRCLDRYFG-MDCGIPEAAWYGRYKHHFP-GTLLRRR------EVPRRIINGFPVN
Dappul_GT17_MGAT3A        LPNDLSCFTTGTDLMK-----------------------------SSSS--------------------------------------PPGSSCTCLAGYYG-PDCGIPGPVWH-ACQTLNN-CSDLKVR------KTPRRLIHGLNIN
Anasim_GT17_MGAT3A        VN-GTDCFVLGTDLEY-----------------------------MKRTEDG-----------------------------------FATDIKCACKQGWHG-RDCGQPEVIWRAFIAS----RQKIFPRK----RRQPRHLVLAFSVI
Dromel_GT17_MGAT3_05573   LTPNLWCYREGTINE------------------------------SQRLNDVDY---------------------------------LMAPPQCRCESGWHG-RDCGQPEIIWRALMTS-----NRASKRG----GSTPLQLVEASSSS
                               *                                                                                 *                                *          :
Prim_cons                 TKAGGVCFKPGTKMLTPKD22GERPGKPGGATPSGARVGSRAGAPEERKELAAGSSSKQ2PLARGPAR2SMPKLLSARERTGGSRGTKRRKWVECVCLPGWHGQPSCGVPTVVQYSNLPT2H2RKERLVPR3IYLR4EVPRRVINAINVN
```

```
                                610       620       630       640       650       660       670       680       690       700       710       720       730       740       750
                                  |         |         |         |         |         |         |         |         |         |         |         |         |         |         |
Nemvec_GT17_MGAT3_05571_A -HEFVMLEAILHDLYDLVDVFIFVESVYTAYGTRKPLRLLQRLHR--GYLKDFHPKIIYLSIDHFPTGGRAKGFIADSYLRSYLGKFGIPHIKYLKDDDLFLVFDLDEIPSRDSLIFLKVHDGFPEPFGFRMRWSAFGFFWKHRH-----
Exadia_GT17_MGAT3A_B      -HEFAMLEAILHDLYDLIDVFIIMESPYTAFGVRKPIRLLPRLHR--GYLKXFHPKMMYLYLDHFPTGGKKHGHFADSYLRSYLGKRGMPFIRNLADDDLFLVFDIDEIPSRDSLIFLKVHNRYPEPFGFRFRWSAFGYFWKHRH-----
Exadia_GT17_MGAT3A        -HEFDMLEALLNEHFDLVDVFIILESSYTAFGTRKPLRLLPRLHR--GYLRKFHSKLMYLYCDHFPTGGRKKGWKADSYYRSLLGKKGLTNIHNLKDDDLFIVMDIDEIPSYDSLVFLKYYNRYPEPFGVRLRYSVFGYFWKNNQ-----
Nemvec_GT17_MGAT3A_B      -HEFDMLEALMYELRDLVEVFILVESLYTAFGSRKPLRLLPRLHR--GYLREFHSKILYLSIDHFPNRGRKNGWISDAYLRSYLGKHGVANIRNLRNDDVIIVCDLDEIPSRDSIAFLKFHDGFPEPFGFRLRWSAFGFFWKNSR-----
Salsal_GT17_MGAT3A_rBa    -HEFDLLHARFHELADAVDLFLVCESNFTAYGNSKPLQFLHHLLN--GTYNYVHHKILYVFLDHFPKGGQQNGWIADDYLRTFLSRDGMSRVQGARPDDVFLINDADEIPAREGILFLKLYDGWTEPVSIHMRKSLYGFFWRQPG-----
Oncner_GT17_MGAT3A        -HEFDLLHARFHELADTVDLFLVCESNFTAYGNSKPLHFLRLLLN--GTYNYVHHKILYVFLDHFPKGGQQNGWIADDYLRTFLSRDGMSRVQGARPDDVFLINDADEIPAREGILFLKLYDGWTEPVSIHMRKSLYGFFWRQPG-----
Salsal_GT17_MGAT3A_rBb    -HEFDLLHARFHELADTVDLFLVCESNFTAYGNSKQLHFLRLLLN--GTYNYIRHKILYVFLDHFPKGGQQNGWIADDYLRTFLSYDGMSRVQGTRPDDVFLINDADEIPAREGILFLKLYNGWTEPVSIHMRKSLYGFFWRQPG-----
Oncner_GT17_MGAT3A_B      -HEFDLLHARFHELADTVDLFLVCESNFTAYGNSKPLHFLRLLLN--GTYNYIRHKILYVFLNHFPKGGQQNGWIADDYLRTFLSYDGMSRVQGARPDDVFLINDADEIPAREGILFLKLYDGWTEPVSIHMRKSLYGFFWRQPG-----
Esoluc_GT17_MGAT3A_rB     -HEFDLLHARFHELADAVDVFLVCESNFTAYGSPKPLHFLHFLLN--GTYNYMRHKILYIFLDHFPKGGQQNGWIADDYLRTFLSYDGMSRVKGVRPDDVFLINDADEIPAREGILFLKLYDGWTEPVSIHMRKSLYGFFWKQPG-----
Danrer_GT17_MGAT3A_rB     -HEFDLLHTRFHELADVVDTFLVCESNFTAYGDARPLYLKHLILN--GTFDYIKHKILYIFLDHFPDGGRMDGWIADDYLRTYLTKNGMARIQGLKPDDVFILNDADEIPSRDGILFLKLYDGWTEPVGIHLRKSLYGFFWRQYG-----
Eleele_GT17_MGAT3A_rB     -HEFDLLHTRFHELADAVDVFLVCESNFTAYGKSKPLTFLQLLLN--GTFNYVKHKILYIFLDHFPRGGRINGWIADDYLRTFLTKNGLSRIWGLRPDDVFLLNDADEIPLREGIIFLKLYDGWTEPFGIHLRKSLYGFYWKQLG-----
Cluhar_GT17_MGAT3A_rB     -HELDLLHARFHELADVVDLFLICESNFTAYGARRRLIFFEQLMN--GTFDYVRHKILYVFLDHFPEGGKQNGWIADDYLRTFLSKNGLSRVHGARPDDVFIINDADEIPVWEGILFLKLFDGWTEPFGLHLRKSIYGFFWRQLG-----
Orylat_GT17_MGAT3A        -HEFDLLHARFHELHQAVDLFLICESNFTAYGEKRPLSFLRLLLN--GTYNYIRHKILYVFLDHFPDGGRQDGWIADDYLRTFLTRNGMSRVVGLRPDDVFIINDADEIPAHEGLLFLKLFDGWTEPFAIHMRKSLYGFFWKQFG-----
Orymel_GT17_MGAT3A        -HEFDLLHARFHELHQAVDLFLICESNFTAYGEKRPLSFLRLLLN--GTYNYIRHKILYVFLDHFPDGGRQDGWIADDYLRTFLTRNGMSRVVGLRPDDVFVINDADEIPAHEGLLFLKLFDGWTEPFAIHMRKSLYGFFWKQFG-----
```

```
Latcal_GT17_MGAT3A          -HEFDLLHVRFHELAQAVDLFLVCESNFTAYGERRPLSFLRLLLN--GTYDYIRHKILYVFLDHFPEGGRQDGWIADDYLRTFLTRNGMSRVIGARSDDVFVINDADEIPAHEGLLFLKLFDGWTEPFAIHMRKSLYGFFWKQFG-----
Diclab_GT17_MGAT3A          -HEFDLLHVRFRELAQAVDLFLVCESNFTAYGEKRPLTFLRLLLN--GTYDYIRHKILYVFLDHFPEGGRQDGWIADDYLRTFLTHNGMSRVVGARSDDVFVINDADEIPAHEGLLFLKLFDGWTEPFAIHMRKSLYGFFWKQFG-----
Epimoa_GT17_MGAT3A          -HEFDLLHVRFHELAQAVDVFLICESNFTAYGDKRPLSFLRLLLN--GTYDYIRHKILYVFLDHFPEGGRQDGWIADDYLRTYLTRNGMSRLIGIRPDDVFVINDADEIPAHEGLVFLKLFDGWTEPFAIHMRKSLYGFFWKQFG-----
Ampoce_GT17_MGAT3A          -HEFDLLHVRFHELAQAVDLFLVCESNFTAYGEKRPLSFLRLLLN--GTYDYIRHKILYVFLDHFPEGGRQDGWIADDYLRTFLTRNGMSRVVGARSDDVFVINDADEIPAHEGLLFLKLFDGWTEPFAIHMRKSLYGFFWKQFG-----
Lepocu_GT17_MGAT3A          -HEFDLLQVRFHELAEAVDLFMVCESNFTAYGESRPLHFLRHLLN--GTYDYVRHKILYIFLDHFPDGGRQDGWIADDYLRTYLTRDGMSRVAGVRPDDVFVINDADEIPAKEGVLFLKLFDGWTEPFAIHMRKSLYGFFWKQLG-----
Sclfor_GT17_MGAT3A          -HEFDLLHARFHELAQAVDLFLVCESNFTAYGEERTLHFLRLLLN--GTYDYVRHKILYIFLDHFPEGGRQDGWIADDYLRTFLTRNGMSRIKDLRPDDVFIINDADEIPAQEGVLFLKLFDGWTEPFAIHMRKSLYGFFWKQLG-----
Angang_GT17_MGAT3A          -HEFDLLHARFHELAQAVDLFLVCESNFTAYGESRSLQFLQLLLN--GTYDYVQHKILYVLLDHFPEGGRQDGWIADDYLRTFLTRNGMSRARGVRPDDIFIINDADEIPAQEGVLFLKLFNGWTEPFAIHMRKSLYGFFWKQLG-----
Salsal_GT17_MGAT3A_rAa      -HEFDLLHVRFHELAHAVDLFLVCESNFTAYGERRPLRFLRLLLN--GTYDYVRHKILYVFLDHFPDGGRQDGWIADDYLRTYLTRDGMARVTGSRADDVFVINDADEIPAREGLLFLKLFDGWTEPFAIHMRKSLYGFFWKQLG-----
Salsal_GT17_MGAT3A_rAb      -HEFDLLHVRFHELAHAVDLFLVCESNFTAYGERRPLRFLRLLLN--GTYDYVRHKILYVFLDHFPDGGRQDGWIADDYLRTYLTRDGMARVTGSRADDVFVINDADEIPAREGLLFLKLFDGWTEPFAIHMRKSLYGFFWKQLG-----
Esoluc_GT17_MGAT3A_rA       -HEFDLLHVRFHELAQAVDLFLVCESNFTAYGERRPLRFLRLLLN--GTYDYVRHKILYVFLDHFPEGGRQDGWIADDYLRTYLTRDGMARVTGSRPDDVFVINDADEIPASEGLLFLKLFDGWTEPFAIHMRKSLYGFFWKQLG-----
Danrer_GT17_MGAT3A_rA       -HEFDLLHVRFRELLQAVDVFLVCESNFTAYGERRPLRFLNLLLN--GTYDYVRHKILYVFLDHFPDGGRQDGWIADDYLRTFLTRNGISRVAGMRPDDVFLINDADEIPAQEGILFLKLFDGWTEPFAIHMRKSLYGFFWKQLG-----
Cluhar_GT17_MGAT3A_rA       -HEFDLLHVRFHELVDTVDLFLVCESNFTAYGDRRPLRFLRRLLN--GTYDYIRHKILYVFLDHFPDGGRQDGWIADDYLRTFLTRNGMSRVAGIRPDDVFLINDADEIPAREGILFLKLFDGWTEPFAIHMRKSLYGFFWKQLG-----
Eleele_GT17_MGAT3A_rA       -HEFDLLHVRFRELADAVDLFLVCESNFTAYGERRSLGFLRLLLN--GTYNYVRHKILYVFLDHFPDGGRQDGWIADDYLRTFLTRNGMTRLRGARSDDVFLINDADEIPAREGILFLKLFDGWTEPFAIHMRKSLYGFYWKQLG-----
Amioce_GT17_MGAT3A          -HEFDLLHVRFHELGPAVDLFLVCESNFTAYGEARPLHFLQKLLD--GTFDYVRHKILYVFLDHFPDGGRQDGWIADDYLRTYLTRDGLARVGGARPDDVFIINDADEIPAREGVLFLKLFDGWTEPFAIHMRKSLYGFFWKQLG-----
Acirut_GT17_MGAT3A          -HEFDLLHVRFHELADAVDVFVVCESNFTAYGDRRPLHFLKQLLN--GTYDYVRHKILYVFLDHFPDGGRQDGWIADDYLRTFLTRDSVQRIQGLRPDDVFIINDADEIPAYEGVLFLKLFDGWTEPFAIHMRKSLYGFFWKQPG-----
Acirut_GT17_MGAT3A_B        -HEFDLLHVRFHELADAVDVFVVCESNFTAYGDRRPLHFLKQLLN--GTYDYVRHKILYVFLDHFPDGGRQDGWIADDYLRTFLTRDSVRRIQGLRPDDVFIINDADEIPAYEGVLFLKLFDGWTEPFAIHMRKSLYGFFWKQPG-----
Polspa_GT17_MGAT3A          -HEFDLLHARFHELADAVDVFAVCESNFTAYGERRPLHFLRQLLN--GTYDYIRHKILYVFLDHFPEGGRQDGWVADDYLRTFLTRDSVRRIQGLRGDDVFIINDADEVPAREGVLFLKLFDGWTEPFAIHMRKSLYGFFWKQPG-----
Galgal_GT17_MGAT3A          -HEFDLLDVRFHELGDVVDVFVVCESNFTAYGEPRPLKFREMLLN--GSYEYIRHKVLYVFLDHFPAGGRQDGWIADDYLRTFLTRDGVSRLRNLRPDDVFIIDDADEIPARDGVLFLKLYDGWTEPFAFHMRKSLYGFFWKQPG-----
Melgal_GT17_MGAT3A          -HEFDLLDVRFHELGDVVDVFVVCESNFTAYGEPRPLKFREMLLN--GSYEYIRHKVLYVFLDHFPAGGRQDGWIADDYLRTFLTRDGVSRLRNLRPDDVFIIDDADEIPARDGVLFLKLYDGWTEPFAFHMRKSLYGFFWKQPG-----
Cotcotjap_GT17_MGAT3A       -HEFDLLDVRFHELGDVVDVFVVCESNFTAYGEPRPLKFREMLLN--GSYEYIRHKVLYVFLDHFPAGGRQDGWIADDYLRTFLTRDGVSRLRNLRPDDVFIIDDADEIPARDGVLFLKLYDGWTEPFAFHMRKSLYGFFWKQPG-----
Aquchrchr_GT17_MGAT3A       -HEFDLLDVRFHELGDVVDAFVVCESNFTAYGEPRPLKFREMLLN--GSFDYIRHKVLYVFLDHFPPGGRQDGWIADDYLRTFLTRDGVSRLRNLRPDDVFIIDDADEIPARDGVLFLKLYDGWTEPFAFHMRKSLYGFFWKQPG-----
Strcamaus_GT17_MGAT3A       -HEFDLLDVRFHELGDVVDAFVVCESNFTAYGEPRPLKFREMLLN--GSFDYIRHKVLYVFLDHFPPGGRQDGWIADDYLRTFLTRDGVSRLRNLRPDDVFIIDDADEIPARDGVLFLKLYDGWTEPFAFHMRKSLYGFFWKQPG-----
Pogvit_GT17_MGAT3A          -HEFDLLDVRFHELGDVVDVFVVCESNFTAYGEPRPLKFREMLLN--SSYDYIRHKVLYVFLDHFPPGGRQDGWIADDYLRTFLTRDGISRLRNLRPDDVFIIDDADEIPARDGVLFLKLYDGWTEPFAFHMRKSLYGFFWKQPG-----
Anocar_GT17_MGAT3A          -HEFDLLDVRFHELGDVVDVFVVCESNFTAYGEPRPLKFREMLLN--GSYDYIRHKVLYVFLDHFPPGGRQDGWIADDYLRTFLTRDGISRLRNLRPDDVFIIDDADEIPARDGVLFLKLYDGWTEPFAFHMRKSLYGFFWKQPG-----
Gekjap_GT17_MGAT3A          -HEFDLLDVRFHELGDVVDAFVVCESNFTAYGEPRPLKFREMLLN--GSFDYIRHKVLYVFLDHFPPGGRQDGWIADDYLRTFLTRDGVSRLRNLRPDDVFIIDDADEIPARDGVLFLKLYDGWTEPFAFHMRKSLYGFFWKQPG-----
Pytbiv_GT17_MGAT3A          -HEFDLLDVRFHELGDVVDAFVVCESNFTAYGEPRPLKFREMLLN--GSFDYIRHKVLYVFLDHFPPGGRQDGWIADDYLRTFLTRDGISRLRNLRPDDVFIIDDADEIPARDGVLFLKLYDGWTEPFAFHMRKSLYGFFWKQPG-----
Thasirsir_GT17_MGAT3A       -HEFDLLDVRFHELGDVVDAFVVCESNFTAYGEPRPLKFREMLLN--GSFDYIRHKVLYVFLDHFPPGGRQDGWIADDYLRTFLTRDGISRFRNLRPDDVFIIDDADEIPARDGVLFLKLYDGWTEPFAFHMRKSLYGFFWKQPG-----
Musmus_GT17_MGAT3A          -HEFDLLDVRFHELGDVVDAFVVCESNFTAYGEPRPLKFREMLTN--GTFEYIRHKVLYVFLDHFPPGGRQDGWIADDYLRTFLTQDGVSRLRNLRPDDVFIIDDADEIPARDGVLFLKLYDGWTEPFAFHMRKSLYGFFWKQPG-----
Muspah_GT17_MGAT3A          -HEFDLLDVRFHELGDVVDAFVVCESNFTAYGEPRPLKFREMLTN--GTFEYIRHKVLYVFLDHFPPGGRQDGWIADDYLRTFLTQDGVSRLRNLRPDDVFIIDDADEIPARDGVLFLKLYDGWTEPFAFHMRKSLYGFFWKQPG-----
Ratnor_GT17_MGAT3A          -HEFDLLDVRFHELGDVVDAFVVCESNFTAYGEPRPLKFREMLTN--GTFEYIRHKVLYVFLDHFPPGGRQDGWIADDYLRTFLTQDGVSRLRNLRPDDVFIIDDADEIPARDGVLFLKLYDGWTEPFAFHMRKSLYGFFWKQPG-----
Mesaur_GT17_MGAT3A          -HEFDLLDVRFHELGDVVDAFVVCESNFTAYGEPRPLKFREMLTN--GTFEYIRHKVLYVFLDHFPPGGRQDGWIADDYLRTFLTQDGVSRLRNLRPDDVFIIDDADEIPARDGVLFLKLYDGWTEPFAFHMRKSLYGFFWKQPG-----
Nangal_GT17_MGAT3A          -HEFDLLDVRFHELGDVVDAFVVCESNFTAYGEPRPLKFREMLTN--GTFEYIRHKVLYVFLDHFPLGGRQDGWIADDYLRTFLTQDGVSRLRNLRPDDVFIIDDADEIPARDGVLFLKLYDGWTEPFAFHMRKSLYGFFWKQPG-----
Hetgla_GT17_MGAT3A          -HEFDLLDVRFHELGDVVDAFVVCESNFTAYGEPRPLKFREMLTN--GTFEYIRHKVLYVFLDHFPPGGRQDGWIADDYLRTFLTQDGVSRLRNLRPDDVFIIDDADEIPARDGVLFLKLYDGWTEPFAFHMRKSLYGFFWKQPG-----
Canlup_GT17_MGAT3A          -HEFDLLDVRFHELGDVVDAFVVCESNFTAYGEPRPLKFREMLTN--GTFEYIRHKVLYVFLDHFPLGGRQDGWIADDYLRTFLTQDGVSRLRNLRPDDVFIIDDADEIPARDGVLFLKLYDGWTEPFAFHMRKSLYGFFWKQPG-----
Vullag_GT17_MGAT3A          -HEFDLLDVRFHELGDVVDAFVVCESNFTAYGEPRPLKFREMLTN--GTFEYIRHKVLYVFLDHFPLGGRQDGWIADDYLRTFLTQDGVSRLRNLRPDDVFIIDDADEIPARDGVLFLKLYDGWTEPFAFHMRKSLYGFFWKQPG-----
Lyncan_GT17_MGAT3A          -HEFDLLDVRFHELGDVVDAFVVCESNFTAYGEPRPLKFREMLTN--GTFEYIRHKVLYVFLDHFPPGGRQDGWIADDYLRTFLTQDGVSRLRNLRPDDVFIIDDADEIPARDGVLFLKLYDGWTEPFAFHMRKSLYGFFWKQPG-----
Felcat_GT17_MGAT3A          -HEFDLLDVRFHELGDVVDAFVVCESNFTAYGEPRPLKFREMLTN--GTFEYIRHKVLYVFLDHFPPGGRQDGWIADDYLRTFLTQDGVSRLRNLRPDDVFIIDDADEIPARDGVLFLKLYDGWTEPFAFHMRKSLYGFFWKQPG-----
Pantigalt_GT17_MGAT3A       -HEFDLLDVRFHELGDVVDAFVVCESNFTAYGEPRPLKFREMLTN--GTFEYIRHKVLYVFLDHFPLGGRQDGWIADDYLRTFLTQDGVSRLRNLRPDDVFIIDDADEIPARDGVLFLKLYDGWTEPFAFHMRKSLYGFFWKQPG-----
Hipampkib_GT17_MGAT3A       -HEFDLLDVRFHELGDVVDAFVVCESNFTAYGEPRPLKFREMLTN--GTFEYIRHKVLYVFLDHFPLGGRQDGWIADDYLRTFLTQDGVSRLRNLRPDDVFIIDDADEIPARDGVLFLKLYDGWTEPFAFHMRKSLYGFFWKQPG-----
Susscr_GT17_MGAT3A          -HEFDLLDVRFHELGDVVDAFVVCESNFTAYGEPRPLKFREMLTN--GTFEYIRHKVLYVFLDHFPLGGRQDGWIADDYLRTFLTQDGVSRLRNLRPDDVFIIDDADEIPARDGVLFLKLYDGWTEPFAFHMRKSLYGFFWKQPG-----
Bostau_GT17_MGAT3A          -HEFDLLDVRFHELGDVVDAFVVCESNFTAYGEPRPLKFREMLTN--GTFEYIRHKVLYVFLDHFPLGGRQDGWIADDYLRTFLTQDGVSRLRNLRPDDVFIIDDADEIPARDGVLFLKLYDGWTEPFAFHMRKSLYGFFWKQPG-----
Vicpac_GT17_MGAT3A          -HEFDLLDVRFHELGDVVDAFVVCESNFTAYGEPRPLKFREMLTN--GTFEYIRHKVLYVFLDHFPLGGRQDGWIADDYLRTFLTQDGVSRLRNLRPDDVFIIDDADEIPARDGVLFLKLYDGWTEPFAFHMRKSLYGFFWKQPG-----
Panpan_GT17_MGAT3A          -HEFDLLDVRFHELGDVVDAFVVCESNFTAYGEPRPLKFREMLTN--GTFEYIRHKVLYVFLDHFPPGGRQDGWIADDYLRTFLTQDGVSRLRNLRPDDVFIIDDADEIPARDGVLFLKLYDGWTEPFAFHMRKSLYGFFWKQPG-----
Pantro_GT17_MGAT3A          -HEFDLLDVRFHELGDVVDAFVVCESNFTAYGEPRPLKFREMLTN--GTFEYIRHKVLYVFLDHFPPGGRQDGWIADDYLRTFLTQDGVSRLRNLRPDDVFIIDDADEIPARDGVLFLKLYDGWTEPFAFHMRKSLYGFFWKQPG-----
Homsap_GT17_MGAT3A          -HEFDLLDVRFHELGDVVDAFVVCESNFTAYGEPRPLKFREMLTN--GTFEYIRHKVLYVFLDHFPPGGRQDGWIADDYLRTFLTQDGVSRLRNLRPDDVFIIDDADEIPARDGVLFLKLYDGWTEPFAFHMRKSLYGFFWKQPG-----
Gorgor_GT17_MGAT3A          -HEFDLLDVRFHELGDVVDAFVVCESNFTAYGEPRPLKFREMLTN--GTFEYIRHKVLYVFLDHFPPGGRQDGWIADDYLRTFLTQDGVSRLRNLRPDDVFIIDDADEIPARDGVLFLKLYDGWTEPFAFHMRKSLYGFFWKQPG-----
Ponpyg_GT17_MGAT3A          -HEFDLLDVRFHELGDVVDAFVVCESNFTAYGEPRPLKFREMLTN--GTFEYIRHKVLYVFLDHFPPGGRQDGWIADDYLRTFLTQDGVSRLRNLRPDDVFIIDDADEIPARDGVLFLKLYDGWTEPFAFHMRKSLYGFFWKQPG-----
Nomleu_GT17_MGAT3A          -HEFDLLDVRFHELGDVVDAFVVCESNFTAYGEPRPLKFREMLTN--GTFEYIRHKVLYVFLDHFPPGGRQDGWIADDYLRTFLTQDGVSRLRNLRPDDVFIIDDADEIPARDGVLFLKLYDGWTEPFAFHMRKSLYGFFWKQPG-----
Hylmol_GT17_MGAT3A          -HEFDLLDVRFHELGDVVDAFVVCESNFTAYGEPRPLKFREMLTN--GTFEYIRHKVLYVFLDHFPPGGRQDGWIADDYLRTFLTQDGVSRLRNLRPDDVFIIDDADEIPARDGVLFLKLYDGWTEPFAFHMRKSLYGFFWKQPG-----
Papanu_GT17_MGAT3A          -HEFDLLDVRFHELGDVVDAFVVCESNFTAYGEPRPLKFREMLTN--GTFEYIRHKVLYVFLDHFPPGGRQDGWIADDYLRTFLTQDGVSRLRNLRPDDVFIIDDADEIPARDGVLFLKLYDGWTEPFAFHMRKSLYGFFWKQPG-----
Macmul_GT17_MGAT3A          -HEFDLLDVRFHELGDVVDAFVVCESNFTAYGEPRPLKFREMLTN--GTFEYIRHKVLYVFLDHFPPGGRQDGWIADDYLRTFLTQDGVSRLRNLRPDDVFIIDDADEIPARDGVLFLKLYDGWTEPFAFHMRKSLYGFFWKQPG-----
Tacacu_GT17_MGAT3A          -HEFDLLEVRFHELGDVVDAFVVCESNFTAYGEPRPLRFRQLLRN--GSFDYIRHKVLYVFLDHFPPGGRQDGWIADDYLRTFLTRDGVARLRNLRPDDVFIIDDADEIPARDGVLFLKLFDGWTEPFAFHLRKSLYGFFWKQPG-----
Ornana_GT17_MGAT3A          -HEFDLLEVRFHELGDVVDAFVVCESNFTAYGEPRPLRFRQLLRN--GSFDYIRHKVLYVFLDHFPPGGRQDGWIADDYLRTFLTRDGVARLRNLRPDDVFIIDDADEIPARDGVLFLKLFDGWTEPFAFHLRKSLYGFFWKQPG-----
Geoser_GT17_MGAT3A          -HEFDLMDVRFHELGSVVDVFIICESNFTAYGDPRPLKLREMLRN--GSYDYIRHKIVYVFLSHFPEGGRQDGWIADDYLRTFLTHDGVSRLINMRDDDIFIINDADEIPARDGVLFLKLYDGWTEPFAFHMRKSLYGFFWKQPG-----
Micuni_GT17_MGAT3A          -HEFDLLDVRFHELGNVVDIFIICESNFTAYGDPRPLKLREMLRN--GSYDYIRHKIVYVFLDHFPEGGRQDGWIADDYLRTFLTHDGVSRLINLRDDDIFIINDADEIPARDGILFLKLYDGWTEPFAFHMRKSLYGFFWKQPG-----
Rhibiv_GT17_MGAT3A          -HEFDLLDVRFHELGSVVDVFVICESNFTAYGDPRPLKLREMLHN--GSYDYIRHKIVYVFLDHFPEGGRQDGWIADDYLRTFLTRDGMGRLLNLREDDIFIINDADEIPARDGILFLKLYDGWTEPFAFHMRKSLYGFFWKQPG-----
Plewal_GT17_MGAT3A          -HEFDLLAARFHELADVVDAFLVCESNFTAYGEPRPLRFREMLLN--GSFDYIRRKVVYIFLDHFPDGGRQDGWIADDYLRTFLTHNGMSRLLNLRDDDIFIIDDADEIPARDGVLFLKLYDGWTEPFAFHMRKSLYGFFWKQPG-----
Lishel_GT17_MGAT3A          -HEFDLIAARFHELVDVVDAFLVCESNFTAYGEARPLRFREMLLN--GSFDYVRRKVVYIFLDHFPDGGRQDGWIADDYLRTFLTRNGMNRLLNLRDDDIFIIDDADEIPARDGVLFLKLYDGWTEPFAFHMRKSLYGFFWKQPG-----
Ambmex_GT17_MGAT3A          -HEFDLLAARFHELADTVDAFLVCESNFTAYGEPRPLRFREMLLN--GSFDYVRRKMVYVFLDHFPEGGRQDGWIADDYLRTFLTRNGMGRLLNLRDDDIFIIDDADEIPAREGVLFLKLYDGWTEPFAFHMRKSLYGFFWKQPG-----
Xenlae_GT17_MGAT3A_rA       -HEFDLLEARFNELKGIVDVFLVCESNFTAYGEQRPLLFRQMLLN--GTFDYIKSKVVYVFLDHFPEGGKQDGWIADDYLRTFLTRNGVGRLRNLRDDDLFIINDADEIPSREGLLFLKLYDGWTEPVAFHMRKSLYGFFWKQPG-----
Xenlae_GT17_MGAT3A_rB       -HEFDLLEARFHELNGIVDVFLVCESNFTAYGEQRSLLFRQMLLN--GTFDYIKSKVVYVFLDHFPEGGKQDGWIADDYLRTFLTHNGVGRLRNLRDDDLFIINDADEIPSREGLLFLKLYDGWTEPVAFHMRKSLYGFFWKQPG-----
Xentro_GT17_MGAT3A          -HEFDLLEARFHELNGVVDVFLVCESNFTAYGEARPLLFRQMLLN--GTFDYIKSKVVYVYLNHFPEGGKQDGWIADDYLRTFLTRNGVGRLRNLRDDDLFIIDDADEIPSREGLLFLKLYDGWTEPVAFHMRKSLYGFFWKQPG-----
Hylsar_GT17_MGAT3A          -HEFDLLEARFRELEGAVDVFLICESNFTAYGEARALLFRQMLLN--GTFEYIKSKVVYVFLDHFPEGGKQDGWIADDYLRTFLTRNGIGRLRNLRDDDLFIIDDADEIPSREGVLFLKLYDGWTEPIAFHMRKSLYGFFWKQPG-----
Bufbuf_GT17_MGAT3A          -HEFDLLEARFRELEGAVDVFLICESNFTAYGEPRPLIFRQMLLN--GTFDYIKSKVVYVFLDHFPEGGKQDGWIADDYLRTFLTHNGISRLRNLRDDDLFIIDDADEIPSREGVLFLKLYDGWTEPIAFHMRKSLYGFFWKQPG-----
Rantem_GT17_MGAT3A          -HEFDLLDARFHELEGAVDVFLICESNFTAYGEARSLLFRQMLLN--GTFDYIKSKVLYVFLNHFPEGGKQDGWIADDYLRTFLTHNGISRLRNLRDDDLFIIDDADEIPSKEGVLFLKLYDGWTEPIAFHMRKSLYGFFWKQPG-----
```

```
Bombom_GT17_MGAT3A           -HEFDLLDVRFNELEGAVDVFLVCESNFTAYGEQRPLLFRQMLLN--GTFDYIKSKIIYVFLDHFPEGGRQDGWIADDYLRTFLTRNGVSRLRNLRDDDLFIIDDADEIPSREGVLFLKLYDGWTEPFAFHMRKSLYGFFWKQPG-----
Latcha_GT17_MGAT3A           -HEFDLLDVRFHELGDVVDVFIICESNFTAYGEPRPLKFQEMLLN--GTYDYVKHKILYVFLDHFPSGGRQDGWIADDYLRTFLTHNGLERIPNVRDDDVFIIDDADEIPARDGVLFLKLFDGWTEPFAFHMRKSLYGFFWKQPG-----
Latcha_GT17_MGAT3A_B         -HEFDLLDVWFHELGDMMDVFIICESNFTASGKPHPLKFQEMLLN--GTYDYIKHKILYVFLDHFPSGRGQDGWIADDYLRTFLTHNGLERIPNVQDDNVFIIDDADEIPARDGVLFLKLFDGWTKPFTFHMRKSLYGFFWKQPS-----
Rhityp_GT17_MGAT3A           -HEFDLLDVRFHELGDVVDVFLICESNFTAFGEPKPLKFIEMLQN--GTYSYVGNKILYVFLDHFPSGGRQDGWIADDYLRTFLTQNGMSRIINLRDDDIFLINDADEIPAHDGVLFLKLFDGWTEPFAFHLRKSLYGFFWKQPG-----
Stetig_GT17_MGAT3A           -HEFDLLDVRFHELGDVVDIFIICESNFTAFGEPKPLKFIEMLQN--GTYSYMGNKILYVFLDHFPSGGRQDGWIADDYLRTFLTQNGMSRIINLRDDDIFLINDADEIPAHDGVLFLKLFDGWTEPFAFHLRKSLYGFFWKQPG-----
Leueri_GT17_MGAT3A           -HEFDLLDVRFHELGSVVDVFIICESNFTAFGDPKPLRFAEMLLN--GTYGYVASKVLYVFLDHFPSGGRQDGWIADDYLRTFLTQNGMSRIANLRDDDIFLIDDADEIPARDGVLFLKLFDGWTEPLAFHLRKSLYGFFWKQPG-----
Pripec_GT17_MGAT3A           -HEFDLLEVRFHELGNVVDVFIICESNFTAFGDPKPLKFIEMLLN--GTYSYIAHKILYVFLDHFPSGGRQDGWIADDYLRTFLTQNGMSRIVNLRDDDIFLIDDADEIPARDGVLFLKLFDGWTEPFAFHLRKSLYGFFWKQPG-----
Calmil_GT17_MGAT3A           -HEFDLLNVRFHELGDVVDVFMVCESNFTAYGDPKPLLFLEMLRN--GTYDQIRHKVLYVFLDHFPSGGRQDGWIADDYLRTFLTRDGLRRMVGVRADDIFVINDADEIPARDGLLFLKLFDGWTEPFGFHLRKSLYGFFWKQPG-----
Lamflu_GT17_MGAT3A           -HEMELLEARLHELNDTVDVFLVCESNFTAFGEARRLHFLSALLS--GAFDFVRHKIHYVFLDHFPSGGRADGWIADEYLRAFATRDGLARLSGLRDDDVFVINDADELPSREALLFARLHDGWPEPFALHLRRSLYGFFWKQPG-----
Letrei_GT17_MGAT3A           -HEMELLEARLHELNDTVDVFLVCESNFTAFGEARRLHFLSALLS--GAFDFVRHKIHYVFLDHFPSGGRADGWIADEYLRAFATRDGLARLSGLRDDDVFVINDADELPSREALLFARLHDGWPEPFALHLRRSLYGFFWKQPG-----
Petmar_GT17_MGAT3A           -HEMELLEARLHELNDTVDVFLVCESNFTAFGEVRRLHFLSALLS--GAFDFVRHKIHYVFLDHFPSGGRADGWIADEYLRTFATRDGLARLSGLRDDDVFVINDADELPSREALLFARLHDGWPEPFALHLRRSLYGFFWKQPG-----
Myxglu_GT17_MGAT3A           -HELDLLEARFNELRGTVDVFVVCESNYTAYGDPRTLLFQRALRN--GSFAAIHGHLLYVFLDHFPAGGRQNGWVADDYLRTYASQQGLARIWGLRPDDLFVINDADELPLREGILFLKLHDGWPEPVGIHLRPSLYGFFWRQPG-----
Limpol_GT17_MGAT3A           -NELELFEIRVWELHHVVDIFLVCESNYTAAGEEKPLRLFKNLQN--GFLQEFQKKIVYVFLDHFPEDGRLNGWAADNYLRTFLGQQGLSQLSSLNDDDLFVLNDADEIPKAEVLEFLRQYDGYPEPISLKMKWYLYGFFWQELKK----
Dapmag_GT17_MGAT3A_A         -HETDFFRVRLEELKDAVDVYIVCESNYTAHGDAKPLYLMEKLRS--GFVENFHSKIVHVSLHKFPPEGRKNGWYIDMYLRTYMGTHGLNRIRGVRSDDLFVLLDADEIPTREVLMFLKLYDGYPEPVRLAMRWSVFGFFWKRKKEKQTG
Dappul_GT17_MGAT3A_B         -HEIEFFRVRLEELKDAVDVYIVCESNYTAHGDAKPLHLMDKLRS--GFMGAYHSKIVHVPLYKFPPEGRENGWFIDMYLRTYMGIHGLKRVHGVRSDDLFVLLDADEIPTREVLMFLKLYDGYPEPVRLAMRWSVFGFFWKRKRGKQTG
Hyaazt_GT17_MGAT3A           -NEVALLEARVNDLAGAVDLFLLAESNYSAHGDPKKLYIKQKLED--GFLREFHHQILHVFVGFFPTRGKTDNWYADQYLRVYMGKMGIPRISGIRDDDLFILNDADEMPSREVVMFLKLYDGYTEPVALALRWSIYGFFFKNLAAEN--
Homame_GT17_MGAT3A           -HEFALFETRMHELYDVVDVFIVGESNYTAHGDPKDLKFLSRFQQ--GYLKEYQEKIVYVSLEFFSLMSQKSGWVADSYLRVYLGEKGLSLLKGVRDDDLFVLSDADELPTREILTFLKVYDGYPEPVTFALRWNVFGFFWRVSKENT--
Dappul_GT17_MGAT3A           -HELEFFQVRLEEVGDVLDVVIVGESNLTAGGDVSPLYLLPELRK--GFMSGFQHKIIHIFIDHFPPEGLTDGWFADTFIRDYMGQEGLKRIKGIRDDDLFLLLDADEIPDRNVLLFLKLYDGYPEPIALTLRWSIYGFFWKRSVSDS--
Anasim_GT17_MGAT3A           NLEIMMAEIMIHELSSIVDLFIIGESNYTETGKPKSQYFLNKLQK--GFLKEMQHKILYVPLTSYDSDKNS----HLQNLRQHMWHKGRQVIKNLQSDDILIMVNAYEIPNIKALLFFKLYNGWPQPVAFRLKWTVYGFFWQHPKK----
Dromel_GT17_MGAT3_05573      LHRLFYMLELGAWDHLSLELLELQIRALIEVVDYFLIYYVSNGSKERSLESMLGSQTSYTLLRCSSESNCTSSMAYSHFRRQLWQQCG----VQMQAQDLLLHGDSGTVYAPAALKFLKYYAKDVLPLKFRLKYNVYGFYWQHPK-----
                              .              ::   .                 .      :  :                     *       .          :::::  :    :     : * :  .      *.  .  ::    :*::::
Prim.cons.                   2HEFDLLDVRFHELGDVVDVFVVCESNFTAYGEPRPLKFREMLLNERGTFDYIRHKILYVFLDHFPPGGRQDGWIADDYLRTFLTRDGVSRLRNLRPDDVFIIDDADEIPARDGVLFLKLYDGWTEPFAFHMRKSLYGFFWKQPG2KQTG

                                        760       770       780       790       800       810       820       830       840       850       860       870       880       890       900
                                          |         |         |         |         |         |         |         |         |         |         |         |         |         |         |
Nemvec_GT17_MGAT3_05571_A    --------------------FSQIPAGCTIGMLRKVYHDDSNIVRDV---HHGVTKNKGNDFQEYRKQYLVSMWYLGEDGGHFAGWHCSSCFHPEGIRTKFLSAQNADWPR-WG-SIKE-KMDLNFIKSMIKQGKWFDGSPLNNRQVNEM
Exadia_GT17_MGAT3A_B         --------------------YSQVPAGCTVGMLRKVYHNDSNMVRDV---HHGVTKNRRQEFIEYRQTTLVRSWFIGEVG-HFAGWHCSSCFKLEGIRIKFMSAQNADWPR-WG-SIPE-KMDLKFLRKMVRDGKWFDGSPLNNRAVAKM
Exadia_GT17_MGAT3A           --------------------YTQVTIGCTIGMLKTVYSNDSNAIRDI---EHGLNAALYPALKKYTENHNVRAWFLGDHT-YYAGWHCSGCFKPEGIRTKFMSAQNADYPR-WG-SIPE-KMDLGYIKTLVKEGKWFDGGEVPS--S---
Nemvec_GT17_MGAT3A_B         --------------------YTQIPAGCTIGLLRTVYNYDTNLVRNI---EDGLNESR-SDFQVYKRAHLIHTWFIGVDN-HYAGWHCSGCFDLQGIRIKFQSAQNGDFPR-WG-AMHE-KMDYEYIKGLVRNGVWFDGSPISDLPIA--
Salsal_GT17_MGAT3A_rBa       --------------------TLDVLSVCTVAMLFTVYNGDGILLRRR---EY-FSMPGFRNYQRKSGHI-LVQWSVGSPV-HYAGWHCSWCFRPEGISQKLISAQNGDFPR-WG-DYME-KRDLNYIKGLIRTGGWFDGS-VGEYPPT--
Oncner_GT17_MGAT3A           --------------------TLEVLSVCTVAMLFTVYNGDGILLRRR---EY-YSMPGFRNYQRESGHI-LVQWSIGSPV-HYAGWHCSWCFRPEGISQKLISAQNGDFPR-WG-DYVE-KRDLNYIKGLIRTGGWFDGS-VGEYPPT--
Salsal_GT17_MGAT3A_rBb       --------------------TLDVHSACTVAMLFAVYNGDGISLRRR---DY-FSMPGFRNYQRESGRI-LVQWSIGSPV-HYGGWHCSWCFRPEGISHKLISAQNGDFPR-WG-DYVE-KRDLNYIKGLIQTGGWFDGS-VGEYPPT--
Oncner_GT17_MGAT3A_B         --------------------TLEVHSACTVAMLFAVYNGDGISLRRR---DY-YFMPGFRNYQRESGHI-LVQWSIGSPV-HYAGWHCSWCFRPEGISHKLISAQNGDFPR-WG-DYVE-KRDLNYIKSLIQTGGWFDGS-VGEYPST--
Esoluc_GT17_MGAT3A_rB        --------------------TLDILSACTVAMLFSVYNRDGISLRRR---EY-FTMPGFRAYEKKNGQI-LLKWSIGSPI-HYAGWHCSWCFSPEGIYYKLISAQNGDFPR-WG-DYAE-KRDLNYIKNLIRTGGWFDGS-VGQYPHT--
Danrer_GT17_MGAT3A_rB        --------------------TLNVLSACTAAMLFKVYKGDGIYLRRR---SY-YTMSGFREYENSTGRI-LIPWAIGSPV-HYAGWHCSWCFKPEGIYYKLVSAQNGDFPR-WG-DYGE-KRKLSYIKDLIRTGGWFDGS-EPDYPPT--
Eleele_GT17_MGAT3A_rB        --------------------TLDVLSGCTVAMLIMVYRWDGILLRRR---DY-YTMPGFRQYENASGSI-LMPWSIGSPV-HYAGWHCSWCFKPEGIHYKLISAQNGDFPR-WG-DFEE-KRKLSYIRGLIRTGGWFDGS-KPQYPPT--
Cluhar_GT17_MGAT3A_rB        --------------------TLDVLSGCTVGMLFAVYKGNGILLRRR---EY-YSMPDFRSYESNTGHI-LIPWSIGSPV-HEAGWHCSWCFKPEGIYYKLISAQNGDFPR-WG-DFEE-KRKLSYIRELIRTGGWFDGS-VSEYPPT--
Orylat_GT17_MGAT3A           --------------------SLEVISGCTVGMLRQVYEGDGIKLRRR---DY-YTMPGFRKYENDTGHI-LVQWSVGSPF-HYAGWHCSWCFKPEGIYFKLVSAQNGDFPR-WG-DYED-KRDLDYIRNLIKTGGWFDGS-MQEYPPA--
Orymel_GT17_MGAT3A           --------------------SLEVISGCTVGMLRNVYDGDGIKLRRR---EY-YTMPGFRKYENDTGHI-LVQWSVGSPF-HYAGWHCSWCFTPEGIYFKLVSAQNGDFPR-WG-DYED-KRDLDYIRNLIKTGGWFDGS-MQEYPPA--
Latcal_GT17_MGAT3A           --------------------SLEVVSGCTVGMLRDVYDGDGIKLRRR---EY-YTMPGFRKYENDTGHI-LVQWSVGSPF-HFAGWHCSWCFTPEGIYFKLVSAQNGDFPR-WG-DYED-KRDLNYIRDLIRTGGWFDGS-LQEYPPV--
Diclab_GT17_MGAT3A           --------------------SLEVVSGCTVGMFRDVYDGDGIKLRRR---EY-YTMPGFRKYENDTGHI-LVQWSVGSPF-HFAGWHCSWCFTPEGIYFKLVSAQNGDFPR-WG-DYED-KRDLNYIRDLIRTGGWFDGS-LQEYPPV--
Epimoa_GT17_MGAT3A           --------------------SLEVISGCTVEMLREVYDGDGIKLRRR---EY-YTMPGFRKYENDTGHI-LVQWSVGSPF-HFAGWHCSWCFTPQGIYFKLVSAQNGDFPR-WG-DYED-KRDLNYIRDLIRTGGWFDGS-LQEYPPA--
Ampoce_GT17_MGAT3A           --------------------SLEVVSGCTLGMLREVYDGDGIKLRRR---EY-YTMPGFRKYENDTGHI-LVQWSVGSPF-HFAGWHCSWCFTPEGIYFKLVSAQNGDFPR-WG-DYED-KRDLNYIRDLIRTGGWFDGS-LQEYPPA--
Lepocu_GT17_MGAT3A           --------------------SLEVVSGCTTGMLRAVYDNDGIKLRRR---EY-YTMPGFRRYENETGHI-LVQWSIGSPF-HFAGWHCSWCFTPEGIYFKLVSAQNGDFPR-WG-DYED-KRDLNYIRELIRTGGWFDGS-VQEYPPS--
Sclfor_GT17_MGAT3A           --------------------SLEVVSGCTVGMLAAVYDCDGIKLRRR---EY-YSMPGFRKYENDTGHI-LVQWSIGSPV-HFAGWHCSWCFPPEGIYYKLISAQNGDFPR-WG-DYED-KRDLKYIRELIQTGGWFDGT-VQEYPPS--
Angang_GT17_MGAT3A           --------------------SLEVVSGCTVGMLNAVYENDGIKLRRR---EY-YTMPGFRRYENDTGHI-LVQWSIGSPF-HFAGWHCSWCFRPEGIHLKLISAQNGDFPR-WG-DYED-KRDINYIKQLIRTGGWFDGS-VQEYPPS--
Salsal_GT17_MGAT3A_rAa       --------------------SLEVVSGCTVGMLRDVYDADGIRLRRR---EY-YTMPGFRKYENDTGHI-LVQWAVGSPF-HFAGWHCSWCFSPEGIHFKLISAQNGDFPR-WG-DYED-KRDLNYIRDLIRTGGWFDGS-VQEYPPS--
Salsal_GT17_MGAT3A_rAb       --------------------SLEVVSGCTVGMLRDVYDADGIRLRRR---EY-YTMPGFRKYENDTGHI-LVQWAVGSPF-HFAGWHCSWCFSPEGIHFKLISAQNGDFPR-WG-DYED-KRDLNYIRDLIRTGGWFDGS-VQEYPPS--
Esoluc_GT17_MGAT3A_rA        --------------------SLEVVSGCTVGMLRDVYDSDGIRLRRR---EY-YTMPGFRKYENNTGHI-LVQWAVGSPF-HFAGWHCSWCFSPEGIHFKLISAQNGDFPR-WG-DYED-KRDLNYIRDLIRTGGWFDGS-VQEYPPS--
Danrer_GT17_MGAT3A_rA        --------------------SLEVVSGCTVGMLRDVYDNDGIRLRRR---EY-YTMPGFRKYENDTGHI-LVQWSIGSPF-HFAGWHCSWCFTPEGIHFKLISAQNGDFPR-WG-DYED-KRDLNYIRELIRTGGWFDGS-VQEYPPS--
Cluhar_GT17_MGAT3A_rA        --------------------SLEVVSGCTVGMLRDVYDNDGIRLRRR---EY-YTMPGFRKYENDTGHI-LVQWSLGSPF-HFAGWHCSWCFSPEGIHFKLISAQNGDFPR-WG-DYED-KRDLKYIRDLIRTGGWFDGS-VQEYPPS--
Eleele_GT17_MGAT3A_rA        --------------------SLEVVSGCTLGMLRNVYDNDGIRLRRR---EY-YTMPGFRKYENDTGHI-LVQWALGSPI-HFAGWHCSWCFTPEGIHLKLVSAQNGDFPR-WG-DYED-KRDLNYIRELIRTGGWFDGT-VQEYPPS--
Amioce_GT17_MGAT3A           --------------------SLEVVAGCTLGMLRAVYDSDGIKLRRR---EY-YTLPGFRRYENITGHI-LVQWSLGSPF-HFAGWHCSWCFTPEGIHFKLVSAQNGDFPR-WG-DYED-KRDLGYIRELIRTGGWFDGS-VQEYPPS--
Acirut_GT17_MGAT3A           --------------------SLEVVSGCTVAMLRAVYETDGIKLRRR---EY-YTMPGFRAYENETGHI-LVQWSLGSPV-HFAGWHCSWCFRPEGIYFKLVSAQNGDFPR-WG-DYEE-KKDLNYIRALIRTGGWFDGT-VQEYPLA--
Acirut_GT17_MGAT3A_B         --------------------SLEVVSGCTVAMLRAVYETDGIKLRRR---EY-YTMPGFRAYENETGHI-LVQWSLGSPV-HFAGWHCSWCFRPEGIYFKLVSAQNGDFPR-WG-DYEE-KKDLNYIRALIRTGGWFDGT-VQEYPLA--
Polspa_GT17_MGAT3A           --------------------SLEVVSGCTVAMLRAVYETDGIKLRRR---EY-YTLPAFRAYENDTGHI-LVQWSLGSPF-HFAGWHCSWCFRPEGIYLKLVSAQNGDFPR-WG-DYEE-KKDLNYIQQLIRTGGWFDGS-VQEYPPA--
Galgal_GT17_MGAT3A           --------------------TLEVVSGCTMGMLQAVYATDGIRLRRR---EY-YTMPGFRQYENSTGHI-LVQWSLGSPL-HFAGWHCSWCFTPEGIYFKLVSAQNGDFPR-WG-DYED-KRDLNYIRELIRTGGWFDGT-TQEYPPA--
Melgal_GT17_MGAT3A           --------------------TLEVVSGCTMGMLQAVYATDGIRLRRR---EY-YTMPGFRQYENSTGHI-LVQWSLGSPL-HFAGWHCSWCFTPEGIYFKLVSAQNGDFPR-WG-DYED-KRDLNYIRELIRTGGWFDGT-TQEYPPA--
Cotcotjap_GT17_MGAT3A        --------------------TLEVVSGCTMGMLQAVYATDGIRLRRR---EY-YTMPGFRQYENSTGHI-LVQWSLGSPL-HFAGWHCSWCFTPEGIYFKLVSAQNGDFPR-WG-DYED-KRDLNYIRELIRTGGWFDGT-TQEYPPA--
Aquchrchr_GT17_MGAT3A        --------------------TLEVVSGCTMGMLQAVYATDGIRLRRR---EY-YTMPGFRQYENSTGHI-LVQWSLGSPL-HFAGWHCSWCFTPEGIYFKLVSAQNGDFPR-WG-DYED-KRDLNYIRELIRTGGWFDGT-TQEYPPA--
Strcamaus_GT17_MGAT3A        --------------------TLEVVSGCTMGMLQAVYATDGIRLRRR---EY-YTMPGFRQYENSTGHI-LVQWSLGSPL-HFAGWHCSWCFTPEGIYFKLVSAQNGDFPR-WG-DYED-KRDLNYIRELIRTGGWFDGS-TQEYPPA--
Pogvit_GT17_MGAT3A           --------------------TLEVVSGCTVGMLQTVYATDGIRLRRR---EY-YTMPGFRQYENSTGHI-LVQWSLGSPL-HFAGWHCSWCFTPEGIYFKLVSAQNGDFPR-WG-DYED-KRDLNYIRELIRTGGWFDGT-TQEYPPA--
Anocar_GT17_MGAT3A           --------------------TLEVVSGCTVGMLQAVYATDGIRLRRR---EY-YTMPGFRQYENNTGHI-LVQWSLGSPL-HFAGWHCSWCFTPEGIYFKLVSAQNGDFPR-WG-DYED-KRDLNYIRELIRTGGWFDGT-TQEYPAA--
Gekjap_GT17_MGAT3A           --------------------TLEVVSGCTIGMLQTVYATDGIRLRRR---EY-YTMPGFRQYENSTGHI-LVQWSLGSPL-HFAGWHCSWCFTPEGIYFKLVSAQNGDFPR-WG-DYED-KRDLNYIRELIRTGGWFDGT-IQEYPPA--
Pytbiv_GT17_MGAT3A           --------------------TLEVVSGCTVGMLQAVYATDGIRLRRR---EY-YTMPGFRQYENSTGHI-LVQWSLGSPL-HFAGWHCSWCFTPEGIYFKLVSAQNGDFPR-WG-DYED-KRDLNYIRELIRTGGWFDGT-TQEYPPA--
```

```
Thasirsir_GT17_MGAT3A     --------------------TLEVVSGCTVGMLQAVYATDGIRLRRR---EY-YTMSGFRQYENSTGHI-LVQWSLGSPL-HFAGWHCSWCFTPEGIYFKLVSAQNGDFPR-WG-DYED-KRDLNYIRELIRTGGWFDGT-TQEYPPA--
Musmus_GT17_MGAT3A        --------------------TLEVVSGCTMDMLQAVYGLDGIRLRRR---QY-YTMPNFRQYENRTGHI-LVQWSLGSPL-HFAGWHCSWCFTPEGIYFKLVSAQNGDFPR-WG-DYED-KRDLNYIRSLIRTGGWFDGT-QQEYPPA--
Muspah_GT17_MGAT3A        --------------------TLEVVSGCTMDMLQSVYGLDGIRLRRR---QY-YTMPNFRQYENRTGHI-LVQWSLGSPL-HFAGWHCSWCFTPEGIYFKLVSAQNGDFPR-WG-DYED-KRDLNYIRSLIRTGGWFDGT-QQEYPPA--
Ratnor_GT17_MGAT3A        --------------------TLEVVSGCTIDMLQAVYGLDGIRLRRR---QY-YTMPNFRQYENRTGHI-LVQWSLGSPL-HFAGWHCSWCFTPEGIYFKLVSAQNGDFPR-WG-DYED-KRDLNYIRSLIRTGGWFDGT-QQEYPPA--
Mesaur_GT17_MGAT3A        --------------------TLEVVSGCTVDMLQAVYELDGIRLRRR---QY-YTMPNFRQYENRTGHI-LVQWSLGSPL-HFAGWHCSWCFTPEGIYFKLVSAQNGDFPR-WG-DYED-KRDLNYIRSLIRTGGWFDGT-QQEYPPA--
Nangal_GT17_MGAT3A        --------------------TLEVVSGCTVDMLQAVYGLDGIRLRRR---QY-YTMPNFRQYENRTGHI-LVQWSLGSPL-HFAGWHCSWCFTPEGIYFKLVSAQNGDFPR-WG-DYED-KRDLNYIRNLIRTGGWFDGT-QQEYPPA--
Hetgla_GT17_MGAT3A        --------------------TLEVVSGCTVDMLQAVYGLDGIRLRRR---QY-YTMPNFRQYENRTGHI-LVQWSLGSPL-HFAGWHCSWCFTPEGIYFKLVSAQNGDFPR-WG-DYED-KRDLNYIRSLIRTGGWFDGT-QQEYPPA--
Canlup_GT17_MGAT3A        --------------------TLEVVSGCTVDMLQTVYGLDGIRLRRR---QY-YTMPNFRQYENRTGHI-LVQWSLGSPL-HFAGWHCSWCFTPEGIYFKLVSAQNGDFPR-WG-DYED-KRDLNYIRSLIRTGGWFDGT-QQEYPPA--
Vullag_GT17_MGAT3A        --------------------TLEVVSGCTVDMLQTVYGLDGIRLRRR---QY-YTMPNFRQYENRTGHI-LVQWSLGSPL-HFAGWHCSWCFTPEGIYFKLVSAQNGDFPR-WG-DYED-KRDLNYIRSLIRTGGWFDGT-QQEYPPA--
Lyncan_GT17_MGAT3A        --------------------TLEVVSGCTVDMLQTVYGLDGIRLRRR---QY-YTMPNFRQYENRTGHI-LVQWSLGSPL-HFAGWHCSWCFTPEGIYFKLVSAQNGDFPR-WG-DYED-KRDLNYIRSLIRTGGWFDGT-RQEYPPA--
Felcat_GT17_MGAT3A        --------------------TLEVVSGCTVDMLQTVYGLDGIRLRRR---QY-YTMPNFRQYENRTGHI-LVQWSLGSPL-HFAGWHCSWCFTPEGIYFKLVSAQNGDFPR-WG-DYED-KRDLNYIRSLIRTGGWFDGT-RQEYPPA--
Pantigalt_GT17_MGAT3A     --------------------TLEVVSGCTVDMLQTVYGLDGIRLRRR---QY-YTMPNFRQYENRTGHI-LVQWSLGSPL-HFAGWHCSWCFTPEGIYFKLVSAQNGDFPR-WG-DYED-KRDLNYIRSLIRTGGWFDGT-RQEYPPA--
Hipampkib_GT17_MGAT3A     --------------------TLEVVSGCTVDMLQTVYGLDGIRLRRR---QY-YTMPNFRQYENRTGHI-LVQWSLGSPL-HFAGWHCSWCFTPEGIYFKLVSAQNGDFPR-WG-DYED-KRDLNYIRSLIRTGGWFDGT-QQEYPPA--
Susscr_GT17_MGAT3A        --------------------TLEVVSGCTVDMLQTVYGLDGIRLRRR---QY-YTMPNFRQYENRTGHI-LVQWSLGSPL-HFAGWHCSWCFTPEGIYFKLVSAQNGDFPR-WG-DYED-KRDLNYIRSLIRTGGWFDGT-QQEYPPA--
Bostau_GT17_MGAT3A        --------------------TLEVVSGCTVDMLQTVYGLDGIRLRRR---QY-YTMPNFRQYENRTGHI-LVQWSLGSPL-HFAGWHCSWCFTPEGIYFKLVSAQNGDFPR-WG-DYED-KRDLNYIRSLIRTGGWFDGT-QQEYPPA--
Vicpac_GT17_MGAT3A        --------------------TLEVVSGCTVDMLQTVYGLDGIRLRRR---QY-YTMPNFRQYENRTGHI-LVQWSLGSPL-HFAGWHCSWCFTPEGIYFKLVSAQNGDFPR-WG-DYED-KRDLNYIRGLIRTGGWFDGT-QQEYPPA--
Panpan_GT17_MGAT3A        --------------------TLEVVSGCTVDMLQAVYGLDGIRLRRR---QY-YTMPNFRQYENRTGHI-LVQWSLGSPL-HFAGWHCSWCFTPEGIYFKLVSAQNGDFPR-WG-DYED-KRDLNYIRGLIRTGGWFDGT-QQEYPPA--
Pantro_GT17_MGAT3A        --------------------TLEVVSGCTVDMLQAVYGLDGIRLRRR---QY-YTMPNFRQYENRTGHI-LVQWSLGSPL-HFAGWHCSWCFTPEGIYFKLVSAQNGDFPR-WG-DYED-KRDLNYIRGLIRTGGWFDGT-QQEYPPA--
Homsap_GT17_MGAT3A        --------------------TLEVVSGCTVDMLQAVYGLDGIRLRRR---QY-YTMPNFRQYENRTGHI-LVQWSLGSPL-HFAGWHCSWCFTPEGIYFKLVSAQNGDFPR-WG-DYED-KRDLNYIRGLIRTGGWFDGT-QQEYPPA--
Gorgor_GT17_MGAT3A        --------------------TLEVVSGCTVDMLQAVYGLDGIRLRRR---QY-YTMPNFRQYENRTGHI-LVQWSLGSPL-HFAGWHCSWCFTPEGIYFKLVSAQNGDFPR-WG-DYED-KRDLNYIRGLIRTGGWFDGT-QQEYPPA--
Ponpyg_GT17_MGAT3A        --------------------TLEVVSGCTVDMLQAVYGLDGIRLRRR---QY-YTMPNFRQYENRTGHI-LVQWSLGSPL-HFAGWHCSWCFTPEGIYFKLVSAQNGDFPR-WG-DYED-KRDLNYIRGLIRTGGWFDGT-QQEYPPA--
Nomleu_GT17_MGAT3A        --------------------TLEVVSGCTVDMLQAVYGLDGIRLRRR---QY-YTMPNFRQYENRTGHI-LVQWSLGSPL-HFAGWHCSWCFTPEGIYFKLVSAQNGDFPR-WG-DYED-KRDLNYIRGLIRTGGWFDGT-QQEYPPA--
Hylmol_GT17_MGAT3A        --------------------TLEVVSGCTVDMLQAVYGLDGIRLRRR---QY-YTMPNFRQYENRTGHI-LVQWSLGSPL-HFAGWHCSWCFTPEGIYFKLVSAQNGDFPR-WG-DYED-KRDLNYIRGLIRTGGWFDGT-QQEYPPA--
Papanu_GT17_MGAT3A        --------------------TLEVVSGCTVDMLQAVYGLDGIRLRRR---QY-YTMPNFRQYENRTGHI-LVQWSLGSPL-HFAGWHCSWCFTPEGIYFKLVSAQNGDFPR-WG-DYED-KRDLNYIRGLIRTGGWFDGT-QQEYPPA--
Macmul_GT17_MGAT3A        --------------------TLEVVSGCTVDMLQAVYGLDGIRLRRR---QY-YTMPNFRQYENRTGHI-LVQWSLGSPL-HFAGWHCSWCFTPEGIYFKLVSAQNGDFPR-WG-DYED-KRDLNYIRGLIRTGGWFDGT-QQEYPPA--
Tacacu_GT17_MGAT3A        --------------------TLEVVSGCTVDMLRAVYATDGIRLRRR---DY-YTMPGFRRYENRTGHI-LVQWSLGSPL-HFAGWHCSWCFPPEGIYLKLVSAQNGDFPR-WG-DYED-KRDLNYIRELIRTGGWFDGT-TQEYPPA--
Ornana_GT17_MGAT3A        --------------------TLEVVSGCTVDMLRAVYATDGIRLRRR---DY-YTMPGFRRYENRTGHI-LVQWSLGSPL-HFAGWHCSWCFPPEGIYLKLVSAQNGDFPR-WG-DYED-KRDLNYIRELIRTGGWFDGT-AQEYPPA--
Geoser_GT17_MGAT3A        --------------------TLEVVSGCTMGMLQMVYGTDGIRLRRR---EY-YTMPNFRQYENHTGHI-LVQWSLGSPL-HFAGWHCSWCFTPEGIYFKLISAQNGDFPR-WG-DYED-KRDLNYIRELIRTGGWFDGN-PQQYPPA--
Micuni_GT17_MGAT3A        --------------------TLEVVSGCTVGMLRMVYGTDGIRLRRR---EY-YTMPNFRQYENYTGHI-LVQWSLGSPL-HFAGWHCSWCFTPEGIYFKLVSAQNGDFPR-WG-DYED-KRDLNYIRELIRTGGWFDGN-SQQYPPA--
Rhibiv_GT17_MGAT3A        --------------------TLEVVSGCTVGMLRMVYGMDGIRLRRR---EY-YTMPNFRQYENHTGHI-LVQWSLGSPL-HFAGWHCSWCFTPEGIYFKLVSAQNGDFPR-WG-DYED-KRDLNYIRELIRTGGWFDGN-TQQYPPA--
Plewal_GT17_MGAT3A        --------------------TLEVVSGCTVAMLREVYATDGIKLRRR---EY-YTMPGFRQYENRTGHI-LVQWSLGSAL-HFAGWHCSWCFTPEGIYFKLISAQNGDFPR-WG-DYAD-KRDLGYIRELIRTGGWFDGT-AQEYPPA--
Lishel_GT17_MGAT3A        --------------------TLEVVSGCTVAMLREVYATDGIRLRRR---EY-YTMPGFRQYENRTGHI-LVQWSLGSAL-HFAGWHCSWCFTPEGIYFKLISAQNGDFPR-WG-DYAD-KRDLGYIRDLIRTGGWFDGT-AQEYPPA--
Ambmex_GT17_MGAT3A        --------------------TLEVVSGCTVAMLREVYATDGIRLRRR---EY-YTMPGFRQYENHTGHI-LVQWSLGSAL-HFAGWHCSWCFTPEGIFFKLVSAQNGDFPR-WG-DYAD-KRDLGYIRDLIRTGGWFDGT-AQEYPPA--
Xenlae_GT17_MGAT3A_rA     --------------------TLDVISACTIGMLHYVYGMDGIRLRRR---EY-YTMPNFRQYENKTGHI-LVQWSVGSPL-HFAGWHCSWCFTPEGIYYKLISAQNGDFPR-WG-DYED-KRDLNYIRWLIKTGGWFDGT-KQEYPPA--
Xenlae_GT17_MGAT3A_rB     --------------------TLDVISACTIGMLSNVYGMDGIRLRRR---EY-YTMPNFRQYENKTGHI-LVQWSVGSPL-HFAGWHCSWCFPPEGIYYKLISAQNGDFPR-WG-DYED-KRDLNYIRWLIKTGGWFDGT-KQEYPPA--
Xentro_GT17_MGAT3A        --------------------TLDVISACTVGMLRYVYGMDGIRLRRR---EY-YTMPNFRQYENKTGHI-LVQWSVGSPL-HFAGWHCSWCFPPEGIYYKLISAQNGDFPR-WG-DYED-KRDLNYIRWLIKTGGWFDGT-KQEYPPA--
Hylsar_GT17_MGAT3A        --------------------TLEVISGCTIGMLRTVYGTDGIRLRRR---EY-YTMPNFRQYENKTGHI-LVQWSLGSPL-HFAGWHCSWCFPPEGIHFKLISAQNGDFPR-WG-DYED-KRDLNYIRWLIKTGGWFDGT-KQEYPAA--
Bufbuf_GT17_MGAT3A        --------------------TLEVISGCTIGMLRTVYGTDGIRLRRR---EY-YTMPNFRQYENKTGHI-LVQWSVGSPL-HFAGWHCSWCFPPEGINFKLISAQNGDFPR-WG-DYED-KRDLNYIRWLIKTGGWFDGT-KQEYPEA--
Rantem_GT17_MGAT3A        --------------------TLEVISGCTIGMLRLVYGTDGIRLRRR---EY-YTMPNFRQYENKTGHI-LVQWSLGSPL-HFSGWHCSWCFPPEGIHFKLISAQNGDFPR-WG-DYED-KRDLNYIRWLIKTGGWFDGT-KQEYPPA--
Bombom_GT17_MGAT3A        --------------------TLEVISGCTIGMLRIVYGTDGIRLRRR---EY-YTMPNFRQYENKTGHI-LVQWSLGSPL-HFAGWHCSWCFPPEGINFKLISAQNGDFPR-WG-DYED-KRDLNYIRWLIKTGGWFDGT-KQEYPPA--
Latcha_GT17_MGAT3A        --------------------SLEVVSGCTVGMLRSVYDTDGIKLRRR---EY-YIMPNFRQYENRTGHI-LVQWSLGSPL-HFAGWHCSWCFRPEGIYYKLISAQNGDFPR-WG-DYEE-KRDLNYIRGLIRTGGWFDGT-VQEYPPA--
Latcha_GT17_MGAT3A_B      --------------------SLEVVLGYTVGMLRSIYDTDGIKLRRQ---EY-YIMPNFRQYENRTGHI-LVQWSLGSPL-HFAGWHCSWCFCPKGIYYKLISAQNGDFPH-WG-DYEE-KRDLNYIHGLIRTGGWFDGT-VQDYPPA--
Rhityp_GT17_MGAT3A        --------------------SLEVISGCTVAMLFMVYNGDGIKLRRR---EY-YLMPNFRLYENSTGHI-LVPWAVGSPL-HFAGWHCSWCFKPEGIRYKLLSAQNGDFPR-WG-NYQE-KRDLNYIRELIQTGGWFDGT-EQDYPPA--
Stetig_GT17_MGAT3A        --------------------SLEVISGCTVAMLFMVYNGDGIKLRRR---EY-YLMPNFRLYENSTGHI-LVPWAVGSPL-HFAGWHCSWCFKPEGIRYKLLSAQNGDFPR-WG-NYQE-KRDLNYIRELIQTGGWFDGT-EQDYPPA--
Leueri_GT17_MGAT3A        --------------------SLEVVSGCTVAMLFMVYNGDGIKLRRR---EY-YLMPNFRKYENSTGHI-LVPWSVGSPL-HFAGWHCSWCFRPEGIRFKLLSAQNGDFPR-WG-NYQE-KRDLSYIRELIQTGGWFDGT-EQEYPPA--
Pripec_GT17_MGAT3A        --------------------SLEVVSGCTVAMLFMVYNGDGIKLRRR---EY-YLMPNFRQYENSTGHI-LVPWSLGSPL-HFAGWHCSWCFKPEGIRYKLLSAQNGDFPR-WG-NYQE-KRDLNYIRELIQTGGWFDGT-EQEYPPA--
Calmil_GT17_MGAT3A        --------------------TLEVVAGCTVGMLRSVYEGDGIKLRRR---EY-YLMPSFRQYENATGHI-LVQWSLGSAL-HFAGWHCSWCFSPEGIRHKLVSAQNGDFPR-WG-NYQE-KRELSYIRELIQTGGWFDGT-LQEYPPA--
Lamflu_GT17_MGAT3A        --------------------SLDVLAGSTVGALREVYGGNGIMLRRR---HY-YDMPRFRDYERRSGTR-MERWSVGTAS-RHAGWHCSWCFPPEGVRTKLVSAQNGDFPR-WG-DYPE-KLDMDYIKNLIRTGGWFDGTGAGILPLA--
Letrei_GT17_MGAT3A        --------------------SLDVLAGSTVGALREVYGGNGIMLRRR---HY-YDMPRFRDYERRSGTR-MERWSVGTAS-RHAGWHCSWCFPPEGVRTKLVSAQNGDFPR-WG-DYPE-KLDVDYIKNLIRTGGWFDGTGAGILPLA--
Petmar_GT17_MGAT3A        --------------------SLDVLAGSTVGALREVYGGDGIMLRRR---HY-YDMPRFRDYERRSGTR-MEQWSVGTAS-RHAGWHCSWCFPPEGVRTKLVSAQNGDFPR-WG-DYPE-KLDLDFIKNLIRTGGWFDGTGAGMLPIA--
Myxglu_GT17_MGAT3A        --------------------SLQVLTASTVRTLTEIYNSNGILLRRR---HY-YDMPGFRDYERRSKKR-LAEWALGSSL-HLAGWHCSWCFSPEGILHKLISAQNGDFPR-WG-DYPE-KRDLVYIRELIRTGGWFDGT-LRDLPLA--
Limpol_GT17_MGAT3A        --------------------TIRIRAVSTIKVLKEVWKNDAIELRRE------NMLETYRRTSNNNYSVSLNPWFLGSNE-FHAGWHCSWCFSPAGIQIKLRSAQEDDGIR-WG-DFPE-KYNLVYIKTLIRDGLWFDNT--KTVVKR--
Dapmag_GT17_MGAT3A_A      FILDWLRDAMKEDSENDNEDLLQVTAVSTMNLVWKVCRNNTFLIRKD-MTNYADFQERLSQY-RIEGNR-ITPWTAGTKH-HYAGYHCSWCYSPEGIRTKLLSAHADDKPR-WG-DFAE-KLDIAYISRLIREGEWFDAT--HPFLMAD-
Dappul_GT17_MGAT3A_B      SIFNWIMDVVKED-DNNAEDLLEVTAVSTMNLVWKVCQNNTFLIRKD-MTNNADFQERLSQY-RTEGNR-VSPWTSGTKQ-AYAGHHCSWCYSPEGIRTKLLSAHADDKPR-WG-DFAE-KLDLRYISRLIREGEWFDSS--RPFVMAD-
Hyaazt_GT17_MGAT3A        --------AVTRLLNNRKELTTTVSAVATMGLVTKVLENDPFMIRGR-LTN--AKSELLAAYNRQYPGL-VNLWTAGGVD-HYCGWHCSWCFSPEGIVLKLDSAQAGDKPR-WG-DYPE-KHDLAYLADLIKYGGWFDGS--HNTKLVP-
Homame_GT17_MGAT3A        ---------WLNVINGNKEEVTHVSSVATIGMLRKVLLGNVYNIRKQGMWKNLKMAHSVRSY-RDEGHI-VGDWEAGTVG-HYAGWHCSWCFSPENIVQKLNSAQADDKPR-WG-DYPE-KKNLTYVATRIWRGLWFDDK--THFLPIN-
Dappul_GT17_MGAT3A        ---------------PHVEDVTRIVAISTMRMIRQVYDGKVMSIRRNYLTT-EPLLSRVETY-KKSGHP-VKEWNIGEVG-AYAGFHCSWCYSPEGIKLKLMSAQKDDKPR-WG-DFPD-KLDLGYISGLIQNGRWFDNS--QPFFLVK-
Anasim_GT17_MGAT3A        --------------------TITSPGAWKVQLLDDAYHNNIAKLRTD---------P-----VTDGEETLTYGLVVGDLNHYGGWCCSWCLDPKDIVLTLQEMPNSRSPVNWD-KIKNRKIDPSYIEDLIGAGLWLDSTTELARKSQ--
Dromel_GT17_MGAT3_05573   ----------------------KTLLNGVISSLGHLHSAQLDAHRLH-------R--------------LASSTLGDLN-HYGGWNCELCLPPEQIVLLLQSSSPRKLPVKLPNDTRNAHIDANYMQQLIANGVHIDGT-TQLHRLR--
                                                          .      .    *                        :              * *. *     :   : .              : : .  ::   :  *  :*
Prim_cons                 2I22W22DAVKED3NNNKEDTLEVVSGCTVGMLRAVYGTDGIRLRRR2MTEYGYTMPGFRQYENRTGHILLVQWSLGSPL2HFAGWHCSWCFTPEGIYFKLVSAQNGDFPR2WGNDYED2KRDLNYIRELIRTGGWFDGT2VQEYPPA2M
```

```
                                    910       920       930       940       950       960       970
                                      |         |         |         |         |         |         |
Nemvec_GT17_MGAT3_05571_A   VSAETDSFFAPPYLMKNYDKFLYLMENPYADDEDVDGEDDDGENSAVAFADSCLFIRTF--CVVGIFSFIA----
Exadia_GT17_MGAT3A_B        AEPDIDDFYAPPYILKNPEKFNYLLRNIXLDDEEIEGDEEDDIKVLYSLGQSISVAENFGLWTIGIIVLELYVLN
Exadia_GT17_MGAT3A          -DLQSDHFFAPXYFLRNQDKYRHLLFNPYLESSDR----------------------------------------
Nemvec_GT17_MGAT3A_B        -DPETDRFYAPRYLLQNFERYKDILVNPYRNKTTNLSPYGKIKHNQWL---------------------------
Salsal_GT17_MGAT3A_rBa      --DPKEPMYAPKYLLKYFQLYRYVLDNPYA---------------------------------------------
Oncner_GT17_MGAT3A          --DPKEPMYAPKYLLKYFPLYRYILDNPYA---------------------------------------------
Salsal_GT17_MGAT3A_rBb      --DPKEPMYAPKYLLKNFQLYSYVLENPYA---------------------------------------------
Oncner_GT17_MGAT3A_B        --DPKEPMYAPKYLLKNFPLYRYILENPYV---------------------------------------------
Esoluc_GT17_MGAT3A_rB       --EHKEHMYAPKYMLNNPQRYKYMLENPYAKKH------------------------------------------
Danrer_GT17_MGAT3A_rB       --DPKEHMYAPKYLLDNYDKYHYLLENPYAKGKR-----------------------------------------
Eleele_GT17_MGAT3A_rB       --HPKEHMFAPKYLLDNYERYHYLLENPYAKP-------------------------------------------
Cluhar_GT17_MGAT3A_rB       --DPREHMYAPKYFLDNYDQYEYLLRNLYTEKKDWMVTH------------------------------------
Orylat_GT17_MGAT3A          --DSKEHMYAPKYMLEHYDQYSYLLKNPYSDPKK-----------------------------------------
Orymel_GT17_MGAT3A          --DSKEHMYAPKYMIENHERYSYLLENPYLDPKK-----------------------------------------
Latcal_GT17_MGAT3A          --DPKEHMYAPKYMLEHYDRYRYLLENPYNKGTE-----------------------------------------
Diclab_GT17_MGAT3A          --DPKEHMYAPKYMLEHYDRYRYLLENPYNKASRLSEG-------------------------------------
Epimoa_GT17_MGAT3A          --DPKEHMYAPKYMLEHYDRYRYLLENPYSKMSRLSEG-------------------------------------
Ampoce_GT17_MGAT3A          --DPKEHMYAPKYMLEHYDRYHYLLENPYNKASRLNER-------------------------------------
Lepocu_GT17_MGAT3A          --DPKEHMYAPKYLLENYDRFRYMLENPYAKQGKLSGG-------------------------------------
Sclfor_GT17_MGAT3A          --DPKELMYAPKYLLENYDRYRYMLENPYARKGK-----------------------------------------
Angang_GT17_MGAT3A          --DPKEHMYAPKYLLDNFERYCYMLENPYS---------------------------------------------
Salsal_GT17_MGAT3A_rAa      --DPKEHMYAPKYMLENYEKYLYLLENPYVKHDKMIEG-------------------------------------
Salsal_GT17_MGAT3A_rAb      --DPKEHMYAPKYMLENYEKYLYLLENPYVKHDKMIEG-------------------------------------
Esoluc_GT17_MGAT3A_rA       --DPKEHMYAPKYMLEHYDRYRYLLENPYVKHDALTEG-------------------------------------
Danrer_GT17_MGAT3A_rA       --DPKEHMYAPKYMLEHYDLYRYLLENPYAKQFKPGGA-------------------------------------
Cluhar_GT17_MGAT3A_rA       --DPKEHMYAPKYMLDHYEQYRYLLENPYISPTNLNGG-------------------------------------
Eleele_GT17_MGAT3A_rA       --NPKEHMYAPRHMLDNYNRYRYLLENPYVKQLTLGGG-------------------------------------
Amioce_GT17_MGAT3A          --DPREHMYAPQYLLDNYERYRYMLENPYAKPGDRGWNGGRGGAGGGGAG-------------------------
Acirut_GT17_MGAT3A          --DPKEHMYAPKYLMRNAKKYQYMLRNPYRREGGG----------------------------------------
Acirut_GT17_MGAT3A_B        --DPKEHMYAPKYLMRNSKKYQYMLRNPYRREGGG----------------------------------------
Polspa_GT17_MGAT3A          --DPKEHMYAPRYLLRNPKKYQYMLENPYRREGGG----------------------------------------
Galgal_GT17_MGAT3A          --DPKEHMYAPKYLLKNYQQFHYLLENPYRKAEGAG---------------------------------------
Melgal_GT17_MGAT3A          --DPKEHMYAPKYLLKNYQQFHYLLENPYRKAEGAG---------------------------------------
Cotcotjap_GT17_MGAT3A       --DPKEHMYAPKYLLKNYQQFHYLLENPYRKAEGAG---------------------------------------
Aquchrchr_GT17_MGAT3A       --DPKEQMYAPKYLLKNYQRFRYLLENPYRKAESAG---------------------------------------
Strcamaus_GT17_MGAT3A       --DPKEQMYAPKYLLKNYQRFHYLLENPYRRAEGPG---------------------------------------
Pogvit_GT17_MGAT3A          --DPKEQMYAPKYLLKNYQRFRYLLENPYQKSEGAG---------------------------------------
Anocar_GT17_MGAT3A          --DPKEQMYAPKYLLKNYQRFCYLLENPYQKPEGAG---------------------------------------
Gekjap_GT17_MGAT3A          --DPKEQMYAPKYLLKTYQRFRYLLENPYQKAEGAG---------------------------------------
Pytbiv_GT17_MGAT3A          --DPKEQMYAPKYLLKNYQRFCYLLENPYQKAKGTG---------------------------------------
Thasirsir_GT17_MGAT3A       --DPKEQMYAPKYLLKNYQRFCYLLENPYQKAKGTG---------------------------------------
Musmus_GT17_MGAT3A          --DPSEHMYAPKYLLKNYDQFRYLLENPYREPKSTEEGGRRNQGSDGRPSAVRGKLDTVEG--------------
Muspah_GT17_MGAT3A          --DPSEHMYAPKYLLKNYDQFRYLLENPYLEPKSNVEGGRRNQGSDGRPSAVRGKLDTVEG--------------
Ratnor_GT17_MGAT3A          --DPSEHMYAPKYLLKNYDQFRYLLENPYREPKSTVEGGRRNQGSDGRSSAVRGKLDTTEG--------------
Mesaur_GT17_MGAT3A          --DPSEHMYAPKYLLKNYNKFRYLLDNPYREPKSTAEGGPRNLGSEGKSP-ARGRLETVEG--------------
Nangal_GT17_MGAT3A          --DPSEHMYAPKYLLKHYNQFRYLLDNPYQEPRSTVEGGHRSPGLEGRPPAARGKLDTVEG--------------
Hetgla_GT17_MGAT3A          --DPSEHMYAPKYLLKNYDQFRYLLDNPYQAPKSTAVGGRRNKGTEGRPPAARDKLDTVEG--------------
Canlup_GT17_MGAT3A          --DPSEHMYAPKYLLNNYAQFRYLLDNPYQEPKSTAEGGRRNRGPEGRPP-ARGKLDVVEG--------------
Vullag_GT17_MGAT3A          --DPSEHMYAPKYLLNNYAQFRYLLDNPYQEPKSTAEGGRRNRGPEGRPP-ARGKLDVVEG--------------
Lyncan_GT17_MGAT3A          --DPSEHMYAPKYLLSNYAQFRYLLDNPYREPKGTAEGGRRNRGPEGRPP-ARGRSDVVEG--------------
Felcat_GT17_MGAT3A          --DPSEHMYAPKYLLSNYAQFRYLLDNPYREPKGTAEGGRRNRGPEGRPP-ARGRSDVVEG--------------
Pantigalt_GT17_MGAT3A       --DPSEHMYAPKYLLSNYAQFRYLLDNPYREPKSTAEGGRRNRGPEGRPP-ARGRSDVVEG--------------
Hipampkib_GT17_MGAT3A       --DPSEHMYAPKYLLKNYDRFRYLLDNPYQEPKSTAAGGRRNQGPEGRPP-ARGKLDVVEG--------------
Susscr_GT17_MGAT3A          --DPSEHMYAPKYLLKNYDQFRYLLDNPYQEPKSTADGGRRNKGPQGRPP-ARDKLDVVEG--------------
Bostau_GT17_MGAT3A          --DPSEHMYAPKYLLKNYDQFRYLLDNPYREPKSTAAGGWRNKGSEGRPP-ARGKLDVVEG--------------
Vicpac_GT17_MGAT3A          --NPSEHMYAPKYLLKNYDQFRYLLDNPYQEPKSTAAGGRRNKGPEGRPL-ARGKLDVVEG--------------
Panpan_GT17_MGAT3A          --DPSEHMYAPKYLLKNYDRFHYLLDNPYQEPRSTAAGGRRHRGPEGRPP-ARGKLDETEV--------------
Pantro_GT17_MGAT3A          --DPSEHMYAPKYLLKNYDRFHYLLDNPYQEPRSTAAGGRRHRGPEGRPP-ARGKLDETEV--------------
Homsap_GT17_MGAT3A          --DPSEHMYAPKYLLKNYDRFHYLLDNPYQEPRSTAAGGWRHRGPEGRPP-ARGKLDEAEV--------------
Gorgor_GT17_MGAT3A          --DPSEHMYAPKYLLKNYDRFHYLLDNPYQEPRSTAAGGRRHRGPEGRLP-ARGKLDETEV--------------
Ponpyg_GT17_MGAT3A          --DPSEHMYAPKYLLKNYDRFHYLLDNPYQEPRSTAAGGRRHRGPEGRSP-ARGKLDETEV--------------
Nomleu_GT17_MGAT3A          --DPSEHMYAPKYLLKNYDRFRYLLDNPYQEPRSTAAGGRRHRGPEGRPP-TRGKLDETEV--------------
```

```
Hylmol_GT17_MGAT3A             --DPSEHMYAPKYLLKNYDRFRYLLDNPYQEPRSTAAGGRRHRGPEGRPP-TRGKLDETEV--------------
Papanu_GT17_MGAT3A             --DPSEHMYAPKYLLKNYDQFRYLLDNPYQEPRSTAAGGRRHRGPEGRLP-ARGKLDEAEG--------------
Macmul_GT17_MGAT3A             --DPSEHMYAPKYLLKNYDQFRYLLDNPYQEPRSTAAGGRRHRGPEGRLP-ARGKLDEAEG--------------
Tacacu_GT17_MGAT3A             --DPREHMYAPKYLLQNYRRFRYLLDNPYRRAEGAG---------------------------------------
Ornana_GT17_MGAT3A             --DPKEHMYAPKYLLQNYRRFRYLLDNPYRRAEGAG---------------------------------------
Geoser_GT17_MGAT3A             --DPKEHMYAPKYLLTNYQRFRYLLENPYEKKGGGDKS-------------------------------------
Micuni_GT17_MGAT3A             --DPKEHMYAPKYLLTNYQRFRYLLENPYKKEGGG----------------------------------------
Rhibiv_GT17_MGAT3A             --NPKEHMYAPKYLLKYNQRFRYLLENPYTKKEGGG---------------------------------------
Plewal_GT17_MGAT3A             --DPKEHMFAPKYFLTNFERFRYLLENPYEKAEPGG---------------------------------------
Lishel_GT17_MGAT3A             --DPKEHMFAPKYLLANYERFRYLLENPYEKAESGG---------------------------------------
Ambmex_GT17_MGAT3A             --DPKEHMFAPKYFLANFERFRYLLENPYEKAEPGG---------------------------------------
Xenlae_GT17_MGAT3A_rA          --DPHEHMYAPKYIMENSQRFGYLVKNPYSS--------------------------------------------
Xenlae_GT17_MGAT3A_rB          --DPHEQMYAPKYIMENAQHFGYLVKNPYSS--------------------------------------------
Xentro_GT17_MGAT3A             --DPHEHMYAPKYIMENTQRFSYLVKNPYSS--------------------------------------------
Hylsar_GT17_MGAT3A             --DPHEHMYAPKYIMENLQRFGYLVKNPYS---------------------------------------------
Bufbuf_GT17_MGAT3A             --DPHEHMYAPKCIMENPQRFGYLIKNPYS---------------------------------------------
Rantem_GT17_MGAT3A             --DPHEHMYAPKCVMENPQRFGYLIKNPYS---------------------------------------------
Bombom_GT17_MGAT3A             --DPHEHMYAPKYMMENTKRFGYLIKNPYSS--------------------------------------------
Latcha_GT17_MGAT3A             --DPKEHMYAPKYLMSNMARYHYLLENPYRKTQEEEEEEEEEEE---GKDSSVN---------------------
Latcha_GT17_MGAT3A_B           --DPKEHMYAPKYLMSNMARYHYLLENPGGRGGGGGGGRGGGQRQLCELTPSLH---------------------
Rhityp_GT17_MGAT3A             --DPKEHMYAPKYLLQNFDKYRYILENPYRKENWDQ----ETAMYNQTGKIT-----------------------
Stetig_GT17_MGAT3A             --DPKEHMYAPKYLLQNFDKYRYILENPYRKDNWDQ----EAAMYNQTGKIT-----------------------
Leueri_GT17_MGAT3A             --DPKEHMYAPKYLLKNYGRYKYLLENPYLKESWGQGRRRAVDTYNQTGGIT-----------------------
Pripec_GT17_MGAT3A             --DPKEHMYAPKYLLRNFEKYKYMLENPYRKESWGQGLGGAAVMYNQTGKIT-----------------------
Calmil_GT17_MGAT3A             --DPKEQMYAPKYLLKNFDKYRYILENPYRNEGAES---RDLMASNGSSLLTQLPPQVPMV--------------
Lamflu_GT17_MGAT3A             --NPAEKMYAPWYVLENPKRFGHLLRNPYL---------------------------------------------
Letrei_GT17_MGAT3A             --NPAEKMYAPWYVLENPKKFGHLLRNPYL---------------------------------------------
Petmar_GT17_MGAT3A             --NPAEKMYAPWYVLENPKRFGHLLRNPYL---------------------------------------------
Myxglu_GT17_MGAT3A             --NPSEHLYAPRYLLDNPDKYSYLLQNPYISKASIPPAA------------------------------------
Limpol_GT17_MGAT3A             --NVFSYDDAPYYALKNAERFKHLLQNPFVEEEENAKI-------------------------------------
Dapmag_GT17_MGAT3A_A           --WEKEDQYAPKFMLQHYREFSQLLYPPDMTNVLYSSNSHNRDSQ------------------------------
Dappul_GT17_MGAT3A_B           --LEKEEQYAPKFMLQHPQQFSQLLYPPDMTNELYSSNSHTLDSQ------------------------------
Hyaazt_GT17_MGAT3A             --PD-EVFYAPPFLLKHATRFQSILIHPAQRETFPNSSAFIDDLLTRQ---------------------------
Homame_GT17_MGAT3A             --NTQDRFYAPRYLLDHPEQYRSMLYHPGHPLNLNKTWSPP----------------------------------
Dappul_GT17_MGAT3A             --PEQDPLYAPAYILKHKNRFHYLLDPPSSNKTTSST--------------------------------------
Anasim_GT17_MGAT3A             ---YRDSYFAPEYVLNNTWKYDFLLTNFYMKLDYN----------------------------------------
Dromel_GT17_MGAT3_05573        --EQSEKYFAPEEALQHSSQYGQLLVNLYDVDVLEDLQDED----------------------------------
                                    .   **   :     :  ::

Prim_cons                      2DDPKEHMYAPKYLLKNYDRFRYLLENPYRKPKSTAEGGRRNRGPEGRPPAARGKLDVVEG222GI22222YVLN
```

Figure S13. Multiple sequence alignment of the 99 MGAT3-related amino acid sequences from various Metazoan species: The 99 MGAT3-related sequences (Table S2) were aligned using clustalW.

Figure S14

A)

(((((((((((((((((((Homsap_GT17_MGAT3A:0.00000050,(Panpan_GT17_MGAT3A:0.00000050,(Pantro_GT17_MGAT3A:0.00000050,(Ponpyg_GT17_MGAT3A:
0.00000050,Gorgor_GT17_MGAT3A:0.00000050)0.1400:0.00000000)0.0400:0.00000000)0.1400:0.00000000)0.8000:0.00364021,(Nomleu_GT17_MGAT3A:
0.00000050,Hylmol_GT17_MGAT3A:0.00000050)0.8600:0.00365744)0.5600:0.00308258,(Papanu_GT17_MGAT3A:0.00000050,Macmul_GT17_MGAT3A:0.
00000050)0.7200:0.00057903)0.5800:0.00529447,Hetgla_GT17_MGAT3A:0.00233598)0.2000:0.00024529,(Nangal_GT17_MGAT3A:0.01759790,(Vicpac_G
T17_MGAT3A:0.00640005,(Hipampkib_GT17_MGAT3A:0.00280087,(Susscr_GT17_MGAT3A:0.01178291,(Bostau_GT17_MGAT3A:0.00358530,((Canlup_G
T17_MGAT3A:0.00000050,Vullag_GT17_MGAT3A:0.00000050)0.7800:0.00569063,(Pantigalt_GT17_MGAT3A:0.00246015,(Lyncan_GT17_MGAT3A:0.0000
0050,Felcat_GT17_MGAT3A:0.00000050)0.7000:0.00116505)0.7000:0.00522991)0.3600:0.00193740)0.1000:0.00103645)0.0200:0.00082995)0.0600:0.0009
1517)0.2400:0.00193096)0.1600:0.00244127)0.0200:0.00162253,Mesaur_GT17_MGAT3A:0.00832897)0.2600:0.00132390,((Musmus_GT17_MGAT3A:0.00
173938,Ratnor_GT17_MGAT3A:0.00189776)0.5200:0.00117584,Muspah_GT17_MGAT3A:0.00613509)0.9400:0.01035687)1.0000:0.02629614,(((Melgal_GT
17_MGAT3A:0.00000050,Cotcotjap_GT17_MGAT3A:0.00000050)0.2800:0.00000000,Galgal_GT17_MGAT3A:0.00000050)0.9800:0.02023553,((Aquchrchr_
GT17_MGAT3A:0.00070220,Strcamaus_GT17_MGAT3A:0.00659904)0.9200:0.00933396,((Pytbiv_GT17_MGAT3A:0.00193838,Thasirsir_GT17_MGAT3A:0.
00170793)0.9400:0.01236200,(Gekjap_GT17_MGAT3A:0.00865133,(Pogvit_GT17_MGAT3A:0.00659172,Anocar_GT17_MGAT3A:0.00803940)0.5000:0.002
35393)0.3800:0.00245470)0.7400:0.00350670)0.5600:0.00561144)0.7200:0.00797003)0.5600:0.00467348,(Tacacu_GT17_MGAT3A:0.00367192,Ornana_G
T17_MGAT3A:0.00000000)1.0000:0.05225137)0.8200:0.02266401,(Rhibiv_GT17_MGAT3A:0.03299314,(Geoser_GT17_MGAT3A:0.04027129,Micuni_GT17
_MGAT3A:0.01505837)0.9000:0.01230098)1.0000:0.03549751)0.8200:0.01542140,(((Plewal_GT17_MGAT3A:0.01715641,Lishel_GT17_MGAT3A:0.019295
70)0.5000:0.00522701,Ambmex_GT17_MGAT3A:0.02829137)1.0000:0.05942220,(Bombom_GT17_MGAT3A:0.02873720,(((Xenlae_GT17_MGAT3A-
rA:0.01814745,Xenlae_GT17_MGAT3A-
rB:0.02591635)0.5600:0.00583618,Xentro_GT17_MGAT3A:0.02350120)1.0000:0.03721059,(Rantem_GT17_MGAT3A:0.03451606,(Hylsar_GT17_MGAT3A:
0.01746011,Bufbuf_GT17_MGAT3A:0.02639894)0.5600:0.00466019)0.8400:0.01473311)0.6800:0.00874204)1.0000:0.08930285)0.4800:0.00557446)0.6400:
0.01832444,(Latcha_GT17_MGAT3A:0.00000000,Latcha_GT17_MGAT3A-
B:0.20991064)1.0000:0.08926070)0.3400:0.00585891,(Calmil_GT17_MGAT3A:0.10421818,(Leueri_GT17_MGAT3A:0.05204734,(Pripec_GT17_MGAT3A:0.
03581523,(Rhityp_GT17_MGAT3A:0.00316091,Stetig_GT17_MGAT3A:0.00782278)1.0000:0.04557717)0.9200:0.01746755)1.0000:0.05285403)0.7000:0.02
150636)0.9400:0.03165740,(((Acirut_GT17_MGAT3A:0.00218619,Acirut_GT17_MGAT3A-
B:0.00139945)1.0000:0.04063784,Polspa_GT17_MGAT3A:0.04445289)1.0000:0.04028896,(((Lepocu_GT17_MGAT3A:0.04622812,Amioce_GT17_MGAT3A

:0.05354087)0.6600:0.00638577,(Angang_GT17_MGAT3A:0.07432260,Sclfor_GT17_MGAT3A:0.06271416)0.9600:0.02460281)0.3000:0.00459830,(Eleele_GT17_MGAT3A-rA:0.08242865,((((Salsal_GT17_MGAT3A-rAa:0.00000050,Salsal_GT17_MGAT3A-rAb:0.00000050)1.0000:0.02054395,Esoluc_GT17_MGAT3A-rA:0.01977187)1.0000:0.03264076,(Danrer_GT17_MGAT3A-rA:0.04545740,Cluhar_GT17_MGAT3A-rA:0.04658976)0.4200:0.00280598)0.5000:0.00909105,((Orylat_GT17_MGAT3A:0.01831302,Orymel_GT17_MGAT3A:0.01854146)0.9800:0.03765100,(Epimoa_GT17_MGAT3A:0.03494135,(Latcal_GT17_MGAT3A:0.00849690,(Ampoce_GT17_MGAT3A:0.01230882,Diclab_GT17_MGAT3A:0.02070291)0.4600:0.00237776)0.8600:0.00819399)0.7600:0.01171679)0.9400:0.02102782)0.4600:0.01658085)0.6400:0.00976189)0.8600:0.02273062)0.7400:0.01603862)0.7000:0.03162685,(Cluhar_GT17_MGAT3A-rB:0.14983728,((Danrer_GT17_MGAT3A-rB:0.13906855,Eleele_GT17_MGAT3A-rB:0.10717840)0.9000:0.03106536,(Esoluc_GT17_MGAT3A-rB:0.08393804,((Salsal_GT17_MGAT3A-rBa:0.01993534,Oncner_GT17_MGAT3A:0.01654987)1.0000:0.02573284,(Salsal_GT17_MGAT3A-rBb:0.03414700,Oncner_GT17_MGAT3A-B:0.01309650)1.0000:0.03206721)0.9600:0.03448772)1.0000:0.04762000)0.5600:0.01687721)0.9600:0.02988413)1.0000:0.07435958,Myxglu_GT17_MGAT3A:0.25269357)0.3400:0.01594659,(Petmar_GT17_MGAT3A:0.01196791,(Lamflu_GT17_MGAT3A:0.00661974,Letrei_GT17_MGAT3A:0.00400360)1.0000:0.01482795)1.0000:0.22892126)1.0000:0.17182808,(Limpol_GT17_MGAT3A:0.42408062,((Homame_GT17_MGAT3A:0.39922372,Hyaazt_GT17_MGAT3A:0.35058030)0.9000:0.05619052,(Dappul_GT17_MGAT3A:0.33593063,(Dapmag_GT17_MGAT3A-A:0.05578725,Dappul_GT17_MGAT3A-B:0.08207576)1.0000:0.29811158)0.7800:0.05360487)1.0000:0.11717134)0.7600:0.05532063)0.2600:0.00853080,(Nemvec_GT17_MGAT3A-B:0.26577314,(Exadia_GT17_MGAT3A:0.26171794,(Nemvec_GT17_MGAT3_05571-A:0.13728103,Exadia_GT17_MGAT3A-B:0.19874339)0.9000:0.07179594)0.6000:0.02152872)1.0000:0.29719935)1.0000:0.50978332,Anasim_GT17_MGAT3A:0.45723191,Dromel_GT17_MGAT3_05573:1.17262534);

B)

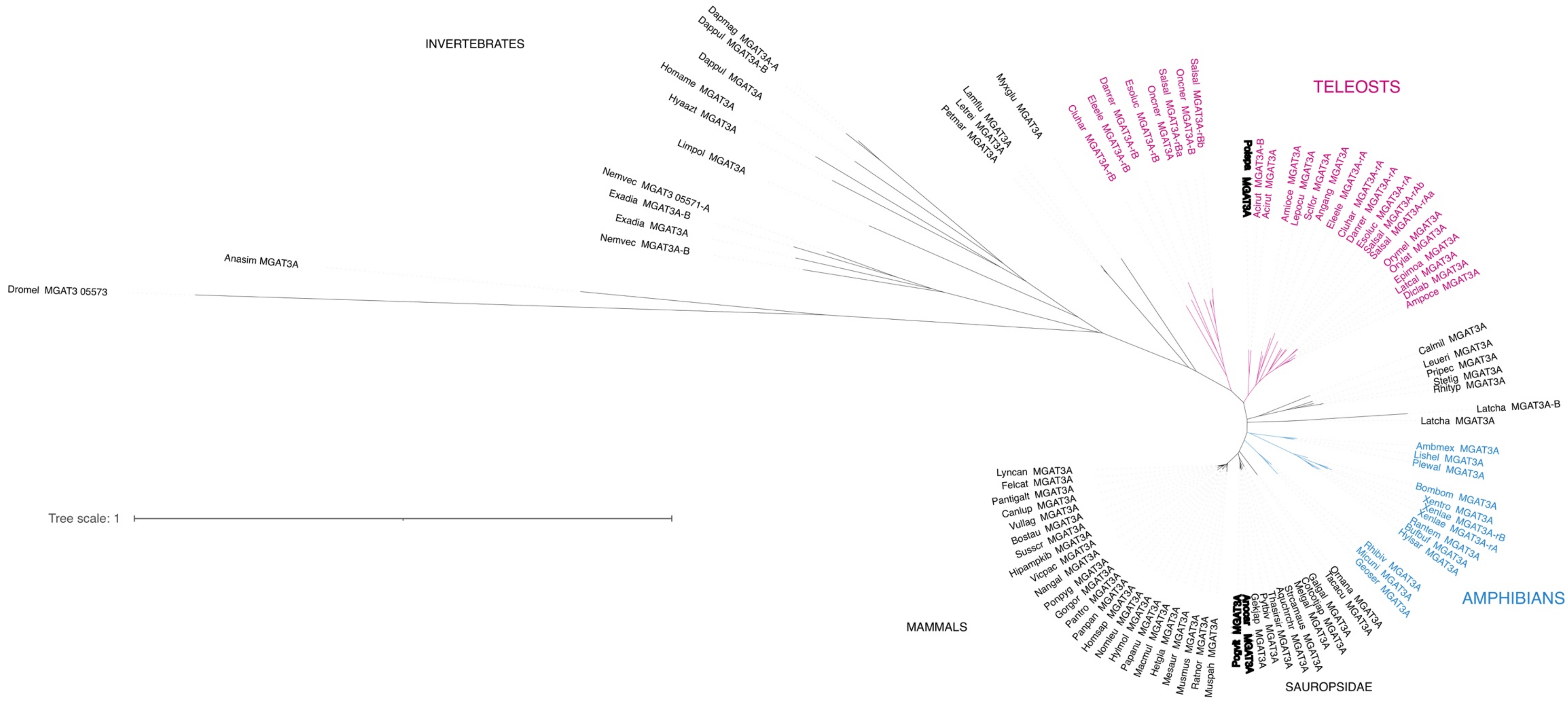

Figure S14. Phylogenetic analysis of MGAT3 sequences. ***A*****,** The Newick tree obtained with the 99 MGAT3-related amino acid sequences from various Metazoan species. ***B***, Phylogenetic tree drawn with iTOL (31), the online tool for phylogenetic tree view.

Figure S15

Mammalian MGAT3 Loop

```
Musmus_GT17_MGAT3A         TKAGGVCFKPGTRML-------EKP-------SPG-R-------TEEKPEVSEGSS------ARGPARRPMRHVLSTRERLGS-RG-TRRKWVECVCLPGWHG-PSCGVPTVVQYSNLPT----KERLVPR------EVPRRVINAININ
Muspah_GT17_MGAT3A         TKAGGVCFKPGTRML-------EKP-------LPG-R-------TEEKPEVAEGSS------ARGPARRPMRHVLSTRERLGS-RG-TRRKWVECVCLPGWHG-PSCGVPTVVQYSNLPT----KERLVPR------EVPRRVINAININ
Ratnor_GT17_MGAT3A         TKAGGVCFKPGTRML-------EKP-------SPG-R-------TEEKTKVAEGSS------VRGPARRPMRHVLSARERLGG-RG-TRRKWVECVCLPGWHG-PSCGVPTVVQYSNLPT----KERLVPR------EVPRRVINAININ
Mesaur_GT17_MGAT3A         TKAGGVCFKPGTKML-------EKP-------S-G-R-------REEKPEVAEGSS------ARGPARKPMRHVLSTPERLGS-RG-TRRKWVECVCLPGWHG-PSCGVPTVVQYSNLPT----KERLVPR------EVPRRVINAININ
Nangal_GT17_MGAT3A         TKAGGVCFKQGTKIL-------EKP-------S-W-G-------RPEKPEVAEGSS------ARGPPRRPTRHVLSARERPGG-RS-PRRKWVECVCLPGWHG-PSCGVPTVVQYSNLPT----KERLVPR------EVPRRVINAININ
Hetgla_GT17_MGAT3A         TKAGGVCFKPGTKML-------EKP-------PSG-R-------PEEKPEGADGSSG-----ARGPARRPPRHVLSAREHAGSSRG-ARRKWVECVCLPGWHG-PSCGVPTVVQYSNLPT----KERLVPR------EVPRRVINAININ
Canlup_GT17_MGAT3A         TKAGGVCFKPGTKML-------ERP-------LSG-R-------PEEKAEGADGSP------ARGPAR----HLLSTRERPGA-RG-ARRKWVECVCLPGWHG-PSCGVPTVVQYSNLPT----KERLVPR------EVPRRVINAININ
Vullag_GT17_MGAT3A         TKAGGVCFKPGTKML-------ERP-------LSG-R-------PEEKAEGADGSP------ARGPAR----HLLSTRERPGA-RG-ARRKWVECVCLPGWHG-PSCGVPTVVQYSNLPT----KERLVPR------EVPRRVINAININ
Lyncan_GT17_MGAT3A         TKAGGVCFKPGTKML-------EKP-------PSG-R-------PDEKAEGADGSS------ARGPAR----HLLSTRERPGA-RG-ARRKWVECVCLPGWHG-PSCGVPTVVQYSNLPT----KERLVPR------EVPRRVINAINVN
Felcat_GT17_MGAT3A         TKAGGVCFKPGTKML-------EKP-------PSG-R-------PDEKAEGADGSS------ARGPAR----HLLSTRERPGP-RG-ARRKWVECVCLPGWHG-PSCGVPTVVQYSNLPT----KERLVPR------EVPRRVINAINVN
Pantigalt_GT17_MGAT3A      TKAGGVCFKPGTKML-------EKP-------PSG-R-------PDEKAEGADGSS------ARGPAR----HLLSTRERPGA-RG-ARRKWVECVCLPGWHG-PSCGVPTVVQYSNLPT----KERLVPR------EVPRRVINAINVN
Hipampkib_GT17_MGAT3A      TKAGGVCFKPGTKML-------EKP-------PSG-R-------PEEKAEAGDGTS------ARGPGR----QLLSTRERPGG-RS-ARRKWVECVCLPGWHG-PSCGVPTVVQYSNLPT----KERLVPR------EVPRRVINAININ
Susscr_GT17_MGAT3A         TKAGGVCFKPGTKTL-------EKP-------PSG-R-------PEEKAEVGDGSS------ARGPAR----HLLSTRERPGA-RS-ARRKWVECVCLPGWHG-PSCGVPTVVQYSNLPT----KERLVPR------EVPRRIINAININ
Bostau_GT17_MGAT3A         TKAGGVCFKPGTKML-------EKP-------PSG-R-------PEEKVEAGDGASSS----ARGPGR----QLLSTRERAGS-RGGARRKWVECVCLPGWHG-PSCGVPTVVQYSNLPT----KERLVPR------EVPRRVINAININ
Vicpac_GT17_MGAT3A         TKAGGVCFKPGTKML-------EKP-------PSG-R-------PEDKAEAGEGSS------ARGPAR----HLLSTRERSGG-RG-GRRKWVECVCLPGWHG-PSCGVPTVVQYSNLPT----KERLVPR------EVPRRVINAININ
Panpan_GT17_MGAT3A         TKAGGVCFKPGTKML-------ERP-------PPG-R-------PEEKPEGANGSS------ARRPPR----YLLSARERTGG-RG-ARRKWVECVCLPGWHG-PSCGVPTVVQYSNLPT----KERLVPR------EVPRRVINAINVN
Pantro_GT17_MGAT3A         TKAGGVCFKPGTKML-------ERP-------PPG-R-------PEEKPEGANGSS------ARRPPR----YLLSARERTGG-RG-ARRKWVECVCLPGWHG-PSCGVPTVVQYSNLPT----KERLVPR------EVPRRVINAINVN
Homsap_GT17_MGAT3A         TKAGGVCFKPGTKML-------ERP-------PPG-R-------PEEKPEGANGSS------ARRPPR----YLLSARERTGG-RG-ARRKWVECVCLPGWHG-PSCGVPTVVQYSNLPT----KERLVPR------EVPRRVINAINVN
Gorgor_GT17_MGAT3A         TKAGGVCFKPGTKML-------ERP-------PPG-R-------PEEKPEGANGSS------ARRPPR----YLLSARERTGG-RG-ARRKWVECVCLPGWHG-PSCGVPTVVQYSNLPT----KERLVPR------EVPRRVINAINVN
Ponpyg_GT17_MGAT3A         TKAGGVCFKPGTKML-------ERP-------PPG-R-------PEEKPEGANGSS------ARRPPR----YLLSARERTGG-RG-ARRKWVECVCLPGWHG-PSCGVPTVVQYSNLPT----KERLVPR------EVPRRVINAINVN
Nomleu_GT17_MGAT3A         TKAGGVCFKPGTKML-------ERP-------PPG-R-------PEEKPEGANGSS------ARRPPR----YLLSARERTGG-RG-ARRKWVECVCLPGWHG-PSCGVPTVVQYSNLPT----KERLVPR------EVPRRIINAINVN
Hylmol_GT17_MGAT3A         TKAGGVCFKPGTKML-------ERP-------PPG-R-------PEEKPEGANGSS------ARRPPR----YLLSARERTGG-RG-ARRKWVECVCLPGWHG-PSCGVPTVVQYSNLPT----KERLVPR------EVPRRIINAINVN
Papanu_GT17_MGAT3A         TKAGGVCFKPGTKML-------ERP-------PPG-R-------PEEKPEGANGSS------ARRPPR----YLLSARERTGG-RG-ARRKWVECVCLPGWHG-PSCGVPTVVQYSNLPT----KERLVPR------EVPRRVINAINVN
Macmul_GT17_MGAT3A         TKAGGVCFKPGTKML-------ERP-------PPG-R-------PEEKPEGANGSS------ARRPPR----YLLSARERTGG-RG-ARRKWVECVCLPGWHG-PSCGVPTVVQYSNLPT----KERLVPR------EVPRRVINAINVN
```

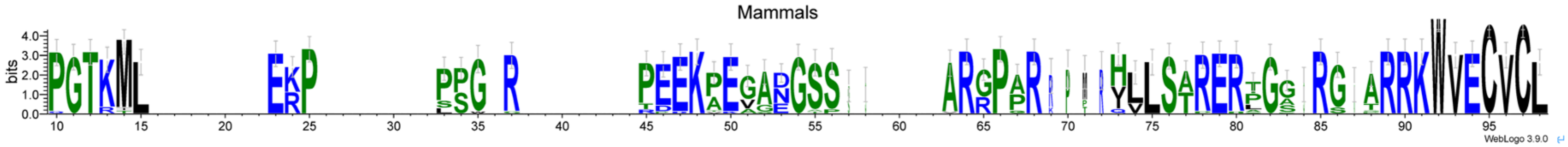

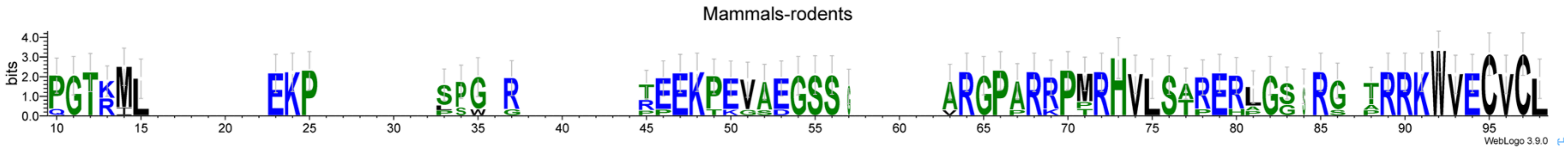

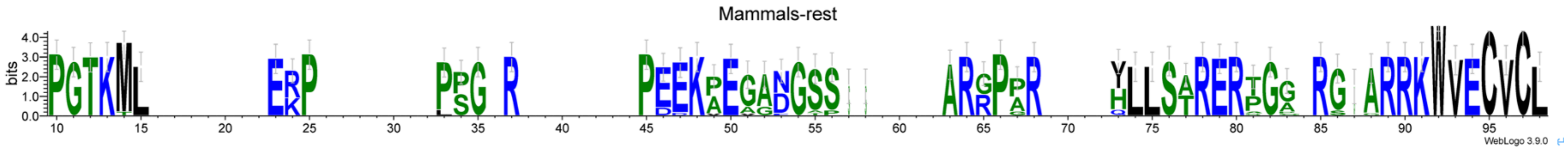

Sauropsidae (Birds and Snakes) MGAT3 Loop

```
Galgal_GT17_MGAT3A        TKAGGVCFKPGTKVL-------EKP-------PAGGR-------PEERVDGVASGR---------PAR----KPLSAG-------GTKRRKWVECVCLPGWHG-PSCGVPTVVQYSNLPT----KDRLVPR------EIPRRVINAININ
Melgal_GT17_MGAT3A        TKAGGVCFKPGTKVL-------EKP-------PVGGR-------PEERADGAASGR---------PAR----KPLSAG-------GTKRRKWVECVCLPGWHG-PSCGVPTVVQYSNLPT----KDRLVPR------EIPRRVINAININ
Cotcotjap_GT17_MGAT3A     TKAGGVCFKPGTKVL-------EKP-------PVGGQ-------LEERADGVASGR---------PAR----KPLSAS-------GTKRRKWVECVCLPGWHG-PSCGVPTVVQYSNLPT----KDRLVPR------EIPRRVINAININ
Aquchrchr_GT17_MGAT3A     TKAGGVCFKPGTKVL-------EKP-------PVGGR-------PEERADGAASGR---------SAR----KPLSAG-------GTKRRKWVECVCLPGWHG-PSCGVPTVVQYSNLPT----KDRLVPR------EIPRRVINAINVN
Strcamaus_GT17_MGAT3A     TKAGGVCFKPGTKVL-------EKP-------PVGGR-------PEERADGAASGR---------PAR----KPLSAS-------GAKRRKWVECVCLPGWHG-PSCGVPTVVQYSNLPT----KDRLVPR------EIPRRVINAINVN
Pogvit_GT17_MGAT3A        TKAGGVCFKPGTKVL-------EKP-------PAG-K-------LDEKTEGAVSGQ---------VGR----KPLSAS-------GTKRRKWVECVCLPGWHG-PSCGVPTVVQYSNLPT----KDRLTPR------EVPRRVINAINVN
Anocar_GT17_MGAT3A        TKAGGVCFKPGTKVL-------EKP-------PPG-R-------PDEKIEGAASGR---------ISR----KPLSAN-------GTKRRKWVECVCLPGWHG-PSCGVPTVVQYSNLPT----KDRLTPR------EVPRRVINAINVN
Gekjap_GT17_MGAT3A        TKAGGVCFKPGTKVL-------EKP-------LVG-R-------LEEKLEGAASER---------AGR----KPLSTN-------GTKRRKWVECVCLPGWHG-PSCGVPTVVQYSNLPT----KDRLTPR------EVPRRVINAINVN
Pytbiv_GT17_MGAT3A        TKAGGVCFKPGTKIL-------EKP-------LAG-R-------PDEKIEGVISGR---------TGR----KPLSAT-------GTKRRKWVECMCLTGWHG-PSCGVPTVVQYSNLPT----KDRLMPR------EVPRRVINAINVN
Thasirsir_GT17_MGAT3A     TKAGGVCFKPGTKIL-------EKP-------LAG-R-------PEEKIEGVISGR---------SGR----KPLSAS-------GTKRRKWVECMCLTGWHG-PSCGVPTVVQYSNLPT----KDRLTPR------EVPRRVINAINVN
```

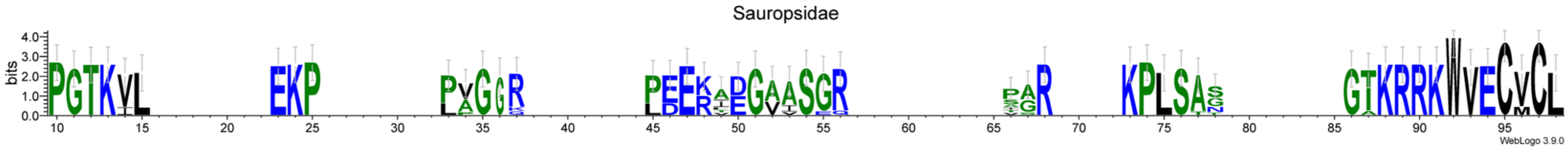

Amphibian MGAT3 Loop

```
Geoser_GT17_MGAT3A        TKSGGMCFKQGTKTF-------ERV-------TTG-H-------AEEKQEHASGHH---------PGR----KPLNVK-------GPKRRKWVECVCLPGWHG-PSCGVPTMVQHSSLPT----KDRLMPR------EVPRRVINAININ
Micuni_GT17_MGAT3A        TKSGGLCFKQGTKKF-------ERV-------PTG-H-------TEEKQEHASGHH---------PGR----KPLNAV-------GPKRRKWVECVCLPGWHG-PSCGVPTVVQYSNLPT----KDRLVPR------EVPRRVINAININ
Rhibiv_GT17_MGAT3A        TKAGGMCFKQGTKML-------EKV-------PTG-Y-------AEERQEHASGRH---------LGR----KPLHMG-------APKRRKWVECVCLPGWHG-PSCGVPTVVQYSNLPT----KDRLVPR------EVPRRIINAININ
Plewal_GT17_MGAT3A        TKAGGLCFKPGTQTL-------DRL-------PTGVH-------GEDRNERPSGRQ---------PAR----RQLSGS------RT-KRKTHVECPCLPGWHG-PSCGVPTVVQYSNLPT----KDRLTPR------AHPRRVINAINVN
Lishel_GT17_MGAT3A        TKAGGLCFKPGTQTL-------DRL-------PAGVH-------GEDRNERPSGRQ---------PTR----KQLSGT------RS-KRKTHVECPCLPGWHG-PSCGVPTVVQYSNLPT----KDRLTPR------AHPRRVINAINVN
Ambmex_GT17_MGAT3A        TKAGGICFKPGTQTI-------DRL-------TAGGH-------GEDRAERPSGHQ---------SPR----KPLGST------RA-KRKTRVECPCLPGWHG-PSCGVPTVVQYSNLPT----KDRLVPR------ARPRRVINAINVN
Xenlae_GT17_MGAT3A_rA     TKSGGVCYKQGTKSL-------DRT-------PSE-K-------PERSFHRKQAAG---------APR--------PQ---------QRRKWVECVCLPGWYG-PSCGVPTVVQHSNLPT----KDRLTPR------LKPRRVINAININ
Xenlae_GT17_MGAT3A_rB     TKSGGVCYKQGTKSL-------DRT-------P-E-K-------PERSSQRRQAPG---------AARQ---QQ--TQ---------QRRKWVECVCLPGWYG-PSCGVPTVVQHSNLPT----KDRLTPR------LKPRRVINAININ
Xentro_GT17_MGAT3A        TKSGGVCYKQGTKSL-------DRT-------PSE-K-------PERSSHRRQAPG---------APRQ---QQ--TQ---------QRRKWVECVCLPGWYG-PSCGVPTVVQHSNLPT----KDRLTPR------SKPRRVINAININ
Hylsar_GT17_MGAT3A        TKSGGVCFKPGTKSL-------DRL-------PSE-K-------PERSLPRKVSPI---------ISRQ---PQQPTQ---------KRRKWVECVCLPGWHG-PSCGVPTVVQHSNLPT----KDRLTPR------LKPRRVINAININ
Bufbuf_GT17_MGAT3A        TKSGGLCFKSGTKSL-------DRL-------PSE-K-------PERSSQRKVSPS---------ISRQ---SQQQIQ---------KRRKWVECVCLPGWHG-PSCGVPTVVQHSNLPT----KDRLTPR------LKPRRVVNAININ
Rantem_GT17_MGAT3A        TKSGGVCFKPGTKAL-------DRP-------LSE-K-------PERPSPRKASSG---------VSRQ---QQPQTQ---------RRRKWVECVCLPGWHG-PSCGVPTVVQHSNLPT----KDRLTPR------LKPRRVINAININ
Bombom_GT17_MGAT3A        TKSGGVCFKPGTKSL-------DRP-------PSE-K-------SERSPPRKQSPG---------TVRQ---QQQPTQ---------KRRKWVDCVCLPGWHG-PSCGVPTVVQHSNLPT----KDRLTPR------LKPRRVINAININ
```

Amphibian group1

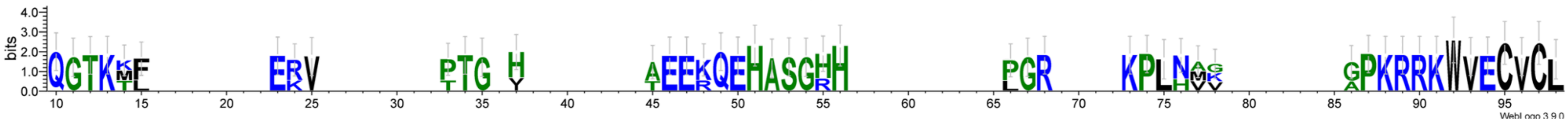

Amphibian group2

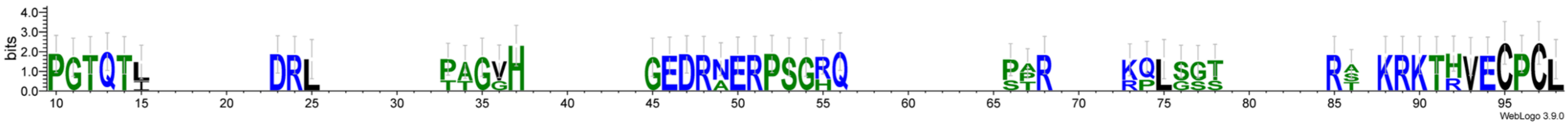

Amphibian group3

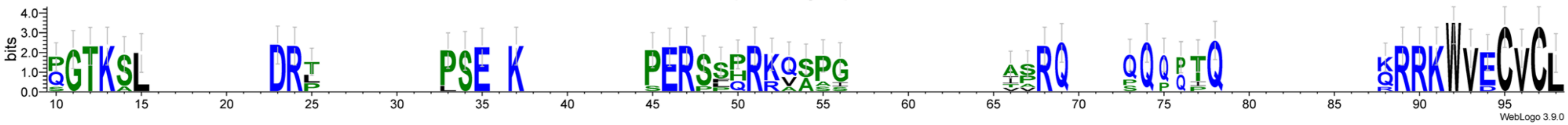

Amphibians

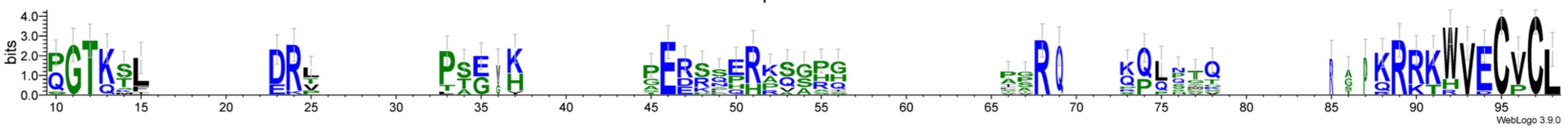

Fish MGAT3 Loop

```
Salsal_GT17_MGAT3A_rBa   TKSGEYCFREGTEITSLKDHLGQKV-------QEP-G-------EGQRKILHAHQE---------EPK----SAQEKTA--------WGKRLVKCMCRPGWHG-PHCGIPTMVQHSNLPT----KARLTLR------KVPRRVINAININ
Oncner_GT17_MGAT3A       TKSGAYCFREGTEMTYLKDHLGQKV-------QEP-G-------EGQRKILHAHQE---------EPK----SAQEKTA--------WGKRLVKCMCRPGWHG-PHCGIPTTVQHSNLPT----KARLTLR------KVPRRVINAININ
Salsal_GT17_MGAT3A_rBb   TKSGAYCFREGTEIAFLKDDSGQRV-------QAA-R-------EGQRKILHAQQE---------EPK----SAQGKSA--------RGKRLVKCMCRQGWHG-PHCGIPTMVQHSNLPN----KGSLMPR------MVPRRVINAININ
Oncner_GT17_MGAT3A_B     TKSGAYCFREGTEMAFLKDDSGQTV-------QAA-R-------EGQRKILHAQQE---------EPK----SAQGKYA--------RGKRLVKCMCRQGWHG-PHCGIPTMVQHSNLPT----KGSLMPR------MVPRRVINAININ
Esoluc_GT17_MGAT3A_rB    TKSGATCFREGTDMAFLK--VGERV-------QQT-G-------VGWHKILHFPQE---------EMK----RDQGQSS--------LGKSLANCTCRPGWHG-PHCGIPTMIQHSNLPS----KWRLMPR------KVPRRVINAININ
Danrer_GT17_MGAT3A_rB    TESGALCFHEGTDLAALA--SQGR-------------------------LLKHYEE---------RTP----LKDDLIK--------SGKKDVRCPCRQGWHG-PQCGIPTVVYHSNLPS----KSKVTPR------KIPRRVINAININ
Eleele_GT17_MGAT3A_rB    TETGDLCFREGMEVASPK--VKERE-------TID-N-------TGLRIILHHQEE---------ENQ----VPMNLT-----------IKVEQCVCRPAWHG-PECGVPTVVQHSNIPT----KSHLSPR------KVPRRIINAININ
Cluhar_GT17_MGAT3A_rB    TKSGRMCFRKGTKTSTKKDNLGESQ-------PKE-S-------YASRRALLAVQS---------GNRS-LGPAWNSSV--------LAKRSVRCRCLQGWHG-YHCGIPTVVQHSNLPT----KSRLAPR------EVPRRVINAINVN
Orylat_GT17_MGAT3A       TKAGALCFKQGTEMASQK----DDAIKMVGAESNMAAEG----AAAQRKPLQLKER---------P---SMSPKTKMRAR-S-----GGKRLVKCVCRPGWHG-PYCGVPTMVYHSNLPT----KERLTPR------ETPRRVINAINVN
Orymel_GT17_MGAT3A       TKAGALCFKQGTEMASQK----DYSVKVV--EPNMAADG----AAVQRKPLELKER---------P---SVSPKTKTRAR-S-----GGKRLVKCVCRPGWHG-PYCGVPTMVYHSNLPT----KERLTPR------ETPRRVINAINVN
Latcal_GT17_MGAT3A       TKAGALCFRQGTEVATPK----EYSGKSGGTAANGAGEGARAGAAGQRKPLEVQQQ---------P---SIAPKAKARAR-G-----NGKRLVKCVCRPGWHG-PYCGVPTMVYHSNLPT----KERLTPR------ETPRRVINAININ
Diclab_GT17_MGAT3A       TKAGALCFRQGTEVATPK----EYSGKSGGAVANGA------GAAGQRKPLEVQQQ---------P---SIAPKAKARAR-G-----NGKRLVKCVCRPGWHG-PYCGVPTMVYHSNLPT----KERLTPR------ETPRRVINAINVN
Epimoa_GT17_MGAT3A       TKAGALCFRQGTEVATPK----EYSGKSGGSVANGAGDG--ARAAGQRKPLEVQQQ---------P---SVAPKAKTRVR-G-----NGKRLVKCVCRPGWHG-PYCGVPTMVYHSNLPT----KERLTPR------ETPRRVINAINVN
Ampoce_GT17_MGAT3A       TKAGALCFRQGTEVATPK----EYSGKPGGAVANGAGGAGRAGAAGQRKPLELQQQ---------S---SIAPKAKVRAR-G-----NGKRLVKCVCRPGWHG-PYCGVPTMVYHSNLPT----KERLTPR------EAPRRVINAINVN
Lepocu_GT17_MGAT3A       TKAGALCFRQGTEMAVPK----SA-----DDRAKG----------SARKILEQPEL----Q-GGGGGGGGGGGAKPKR---------GKRLVKCVCRPGWHG-PYCGVPTMVYHSNLPT----KERLAPR------EVPRRVINAINVN
Sclfor_GT17_MGAT3A       TKSGALCFRQGTEMAVPR----EH-----PGKSGATV-------ASQRRILQHQEA----S-S--------TSHFKAKR---------GKRLVKCVCRQGWHG-PYCGVPTMVYHSNLPT----KKRLTPR------EVPRRIINAININ
Angang_GT17_MGAT3A       TKAGALCFRQGTEMATPK----DS-----VVREGARL-------AGHRRILQHQNT----A-H--------SPRARPQR---------GRRLVRCVCRQGWHG-PYCGIPTMVQHSNLPT----KDRLTPR------EVPRRVINAINVN
Salsal_GT17_MGAT3A_rAa   TKAGALCFRQGTEVATPR----DDAARPGGPTVTGTEVGSRAGSPGQRKPLAVLNQSHQQPLHPSSSGTSMPPKAKSRGR-G------GKRLVKCVCRPGWHG-PYCGVPTMVYHSNLPT----KERLTPR------ERPRRVINAINVN
Salsal_GT17_MGAT3A_rAb   TKAGALCFRQGTEVATPR----DDAARPGGPTVTGTEVGSRAGSPGQRKPLAVLNQSHQQPLHPSSSGTSMPPKAKSRGR-G------GKRLVKCVCRPGWHG-PYCGVPTMVYHSNLPT----KERLTPR------ERPRRVINAINVN
Esoluc_GT17_MGAT3A_rA    TKAGALCFRQGTEAAAPR----EDAARPGGATSTGTGVGPRPGSAGQRKPLEAPNR---HPLQPLSSGTAAPPKAKPRGR-G------GKRLVKCVCRPGWHG-PYCGVPTMVYHSNLPT----KERLTPR------EKPRRVINAINVN
Danrer_GT17_MGAT3A_rA    TKAGALCFRQGTEVAPPK----DE---TLKVKPSAAG-------VIQRRILQKPDE---SKTSGKSVEESRLSRGKPKR---------GKRLVKCVCRAGWHG-PYCGVPTMVYHSNLPT----KERLMPR------ETPRRVINAINVN
Cluhar_GT17_MGAT3A_rA    TKAGALCYRKGTEVAPPP----GQ---AKAVNPTAAAN------HGQRQLLQQPIQ---PQQQ-HPEEQAGTLHAKPKR---------GKRLVKCVCRPGWHG-PYCGVPTMVYHSNLPT----KERLTPR------ETPRRIINAINVN
Eleele_GT17_MGAT3A_rA    TKAGALCFRQGTEAAHPR----DDGGGAGKGRPTASA-------SLQRKILQAGEE---PGTGGRPVEGSSPMRSKPWK---------GKRLVKCVCRAGWHG-PYCGVPTLVQHSNLPT----RERLAPR------DRPRRVINAINVN
Amioce_GT17_MGAT3A       TRAGALCFREGTEMAPPR----GG---AGGTGEAGEG-----AVLGVRKILQEEGS----------RAGGGVAGTRPRGR--------GRRLVKCVCRPGWHG-PYCGVPTMVQHSNLPT----KERLAPR------ERPRRVLNAINVN
```

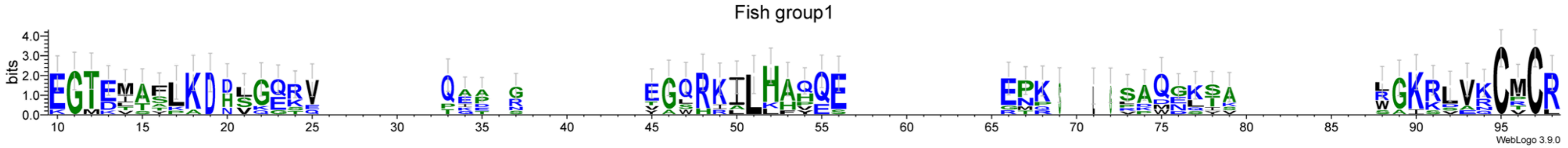

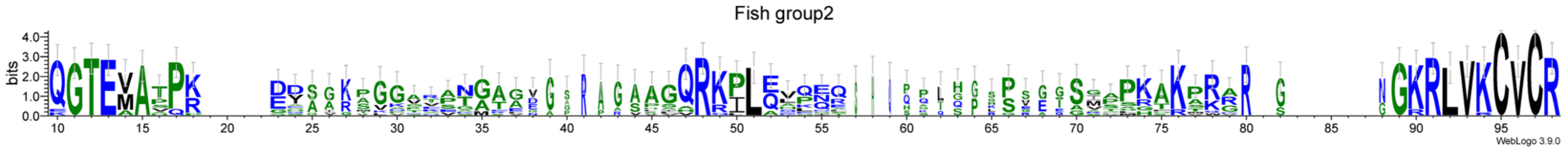

Figure S15. Multiple sequence alignment and sequence logo analysis of MGAT3 orthologs among various species.

Figure S16

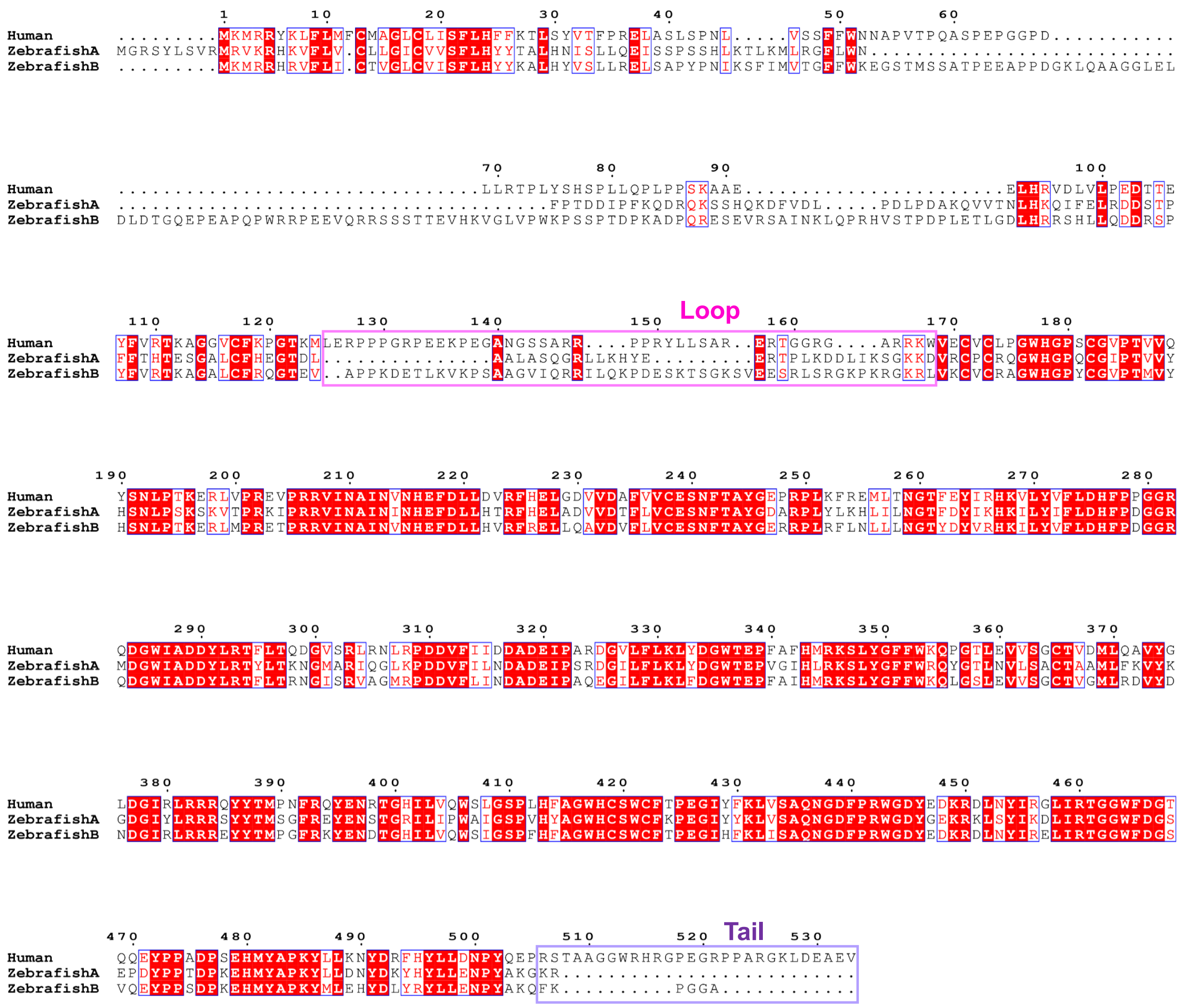

Figure S16. Sequence alignment of MGAT3 between human and zebrafish. The amino acid sequence of human MGAT3 (NCBI accession number NP_001091740) was used as template. Zebrafish-A: *Danio rerio*, NCBI accession number NP_001018485.2; Zebrafish-B: *Danio rerio*, NCBI accession number XP_699442.5. The conserved sites were enclosed in blue, and the conserved residues were shown in red. The Loop was enclosed by magenta rectangle, and the Tail was enclosed by violet rectangle.

Figure S17

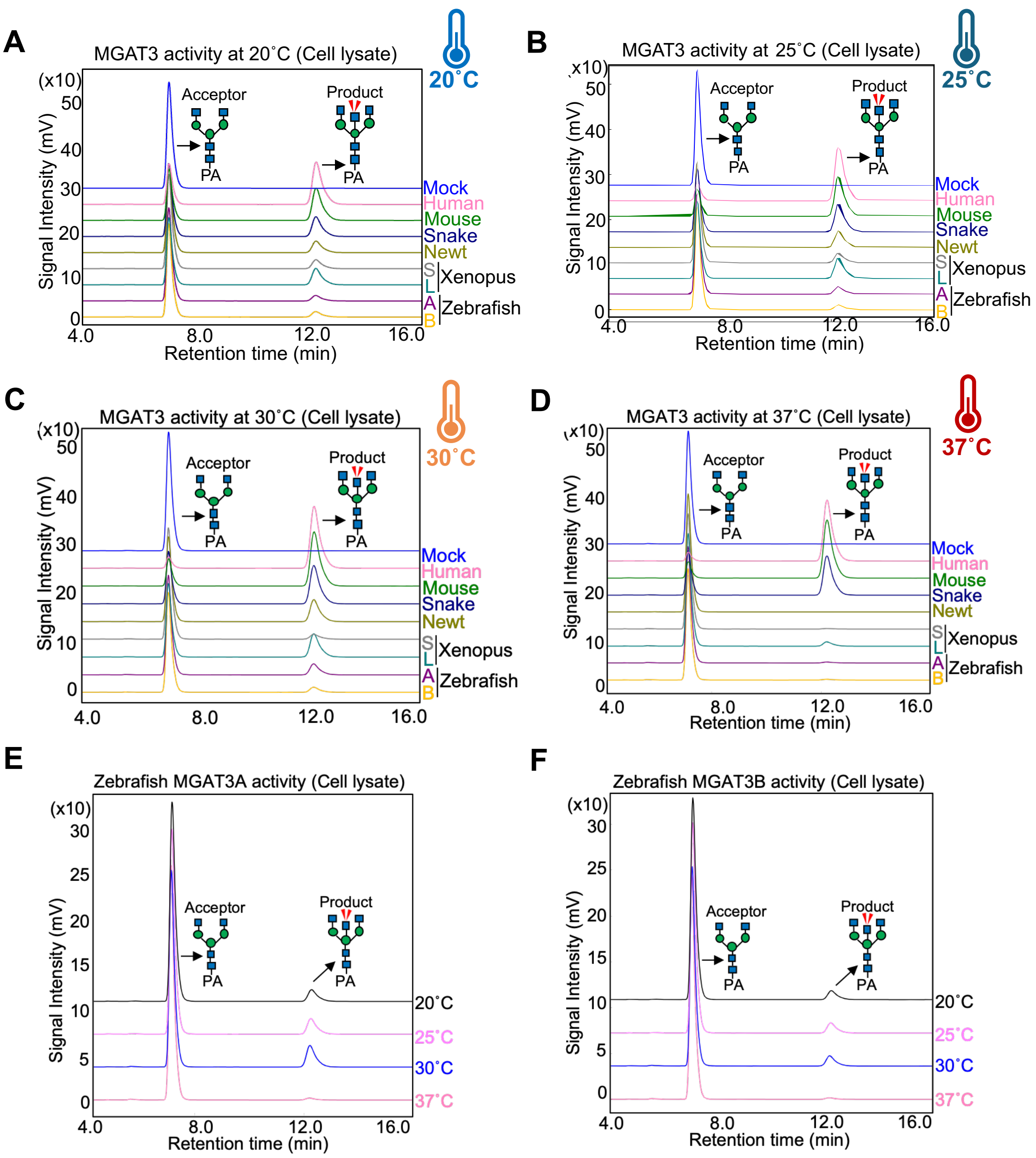

Figure S17. The HPLC-based activity assay of MGAT3 in various vertebrates at different reaction temperatures. The cell lysates of Neuro2A-MGAT3-KO expressing MGAT3 in various vertebrates reacted with the fluorescently labeled oligosaccharide acceptor substrate, GnGnbi-PA at 20˚C (A), 25˚C (B), 30˚C (C) and 37˚C (D). The unreacted substrates and the products with bisecting GlcNAc were quantified by reverse phase HPLC. Activity of zebrafish MGAT3A and MGAT3B at different temperatures are shown in (E) and (F).

Reference

31. Letunic, I., and Bork, P. (2024) Interactive Tree of Life (iTOL) v6: recent updates to the phylogenetic tree display and annotation tool. *Nucleic Acids Res.* **52**, W78–W82
